# Supplementary figures and images for: Adiponectin is essential for lipid homeostasis and survival under insulin deficiency and promotes β-cell regeneration
Source: eLife. 2014 Oct 23;3:e03851. doi: 10.7554/eLife.03851 (PMC4228265; doi:10.7554/eLife.03851)

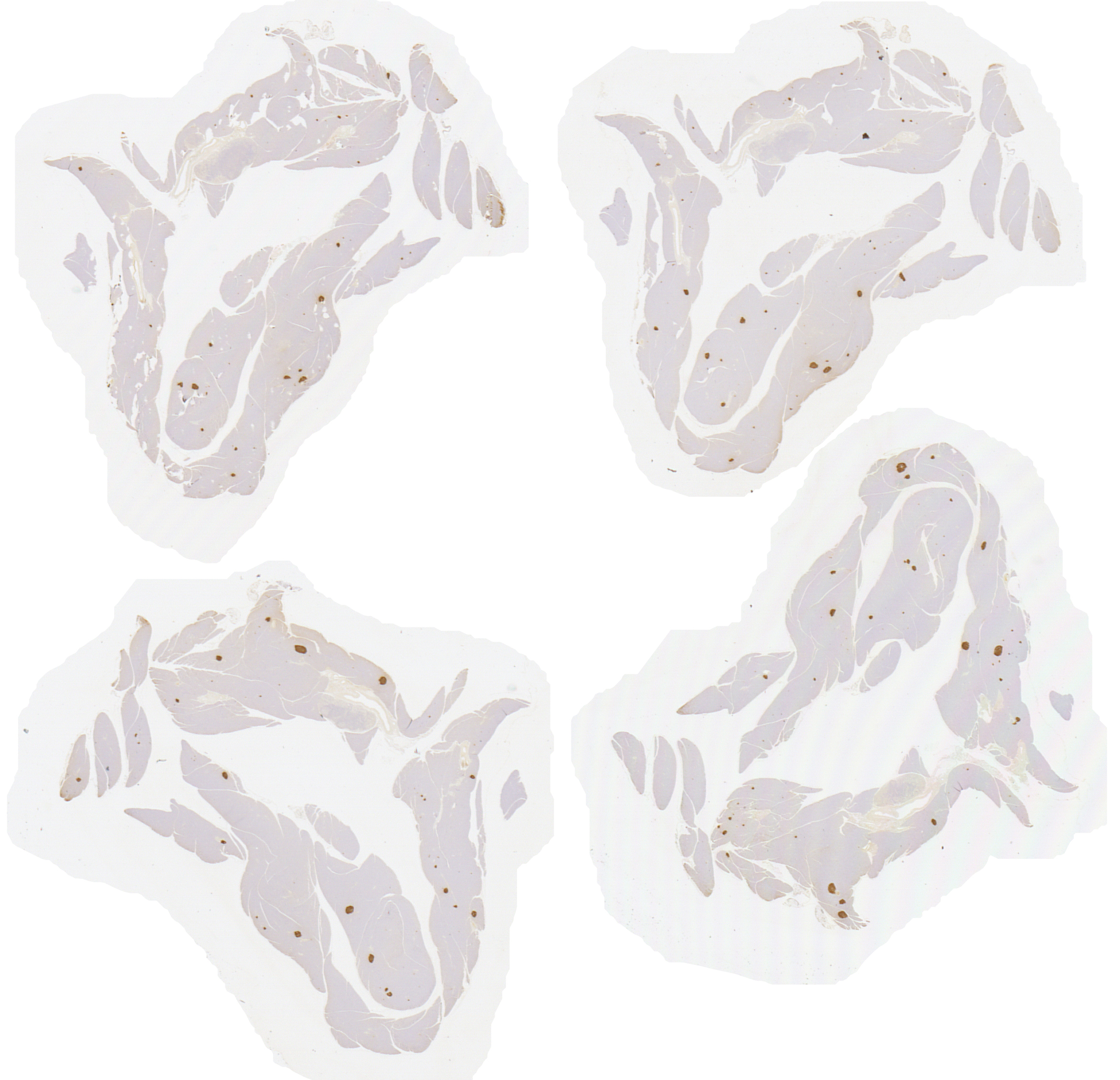

Supplement: Figure 1—source data 1. — The zip file contains all the 2400-dpi scanned images of insulin-immunostained pancreas sections used for quantitation of insulin-positive cell area. Insulin is stained as brown, and the whole section is counterstained as blue with hematoxylin. Folders are named after genotypes (P-Adn++ or P-Adn−−) and subfolders after time points (week 0, 2, 5, or 10) post initial dimerizer administration. Related to Figure 1A. DOI: http://dx.doi.org/10.7554/eLife.03851.004 [file elife03851s001.zip › Figure 1-source data 1/P-Adn--/Week 0/AKO25-6s a.jpg]

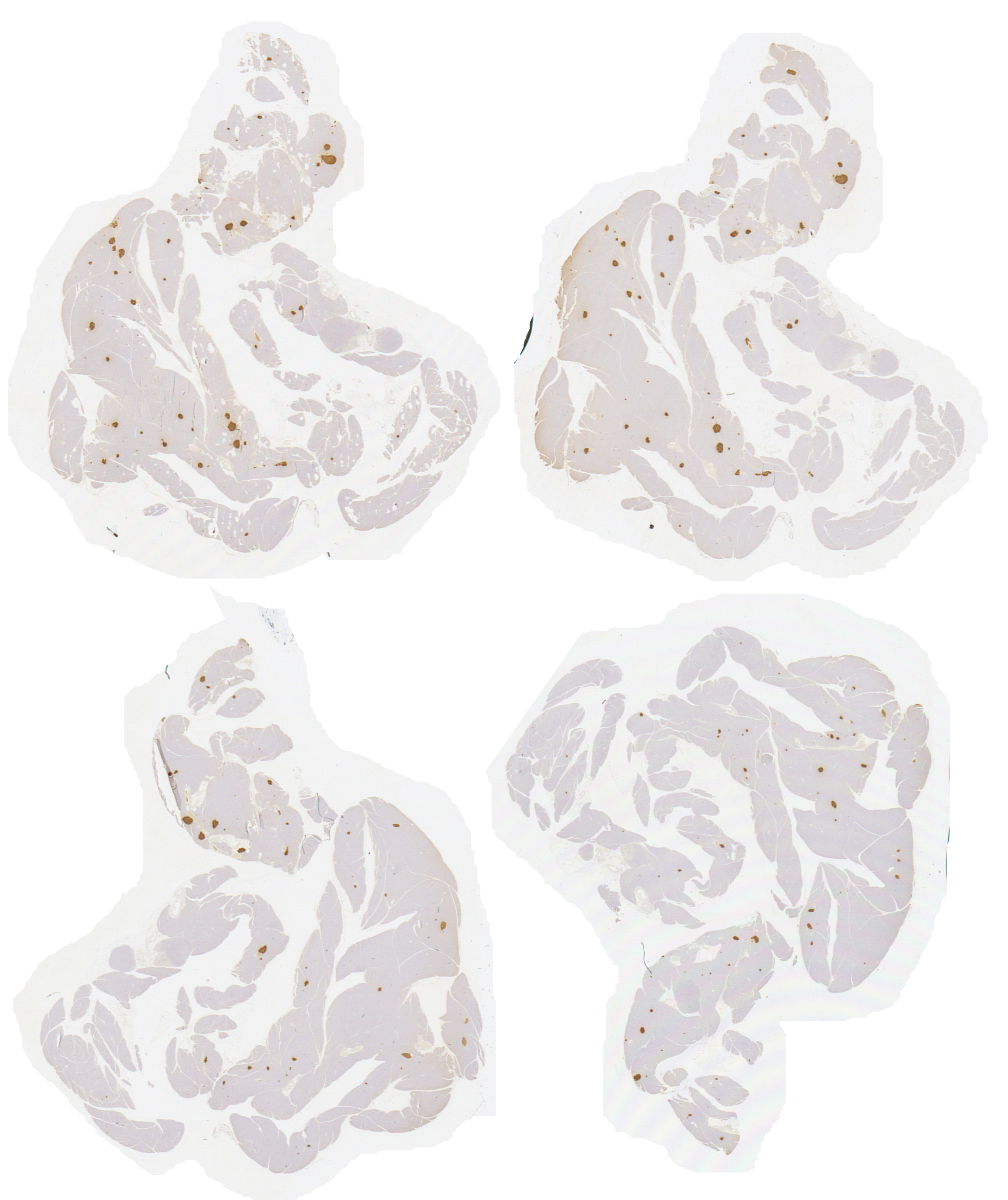

Supplement: Figure 1—source data 1. — The zip file contains all the 2400-dpi scanned images of insulin-immunostained pancreas sections used for quantitation of insulin-positive cell area. Insulin is stained as brown, and the whole section is counterstained as blue with hematoxylin. Folders are named after genotypes (P-Adn++ or P-Adn−−) and subfolders after time points (week 0, 2, 5, or 10) post initial dimerizer administration. Related to Figure 1A. DOI: http://dx.doi.org/10.7554/eLife.03851.004 [file elife03851s001.zip › Figure 1-source data 1/P-Adn--/Week 0/AKO25-6s b.jpg]

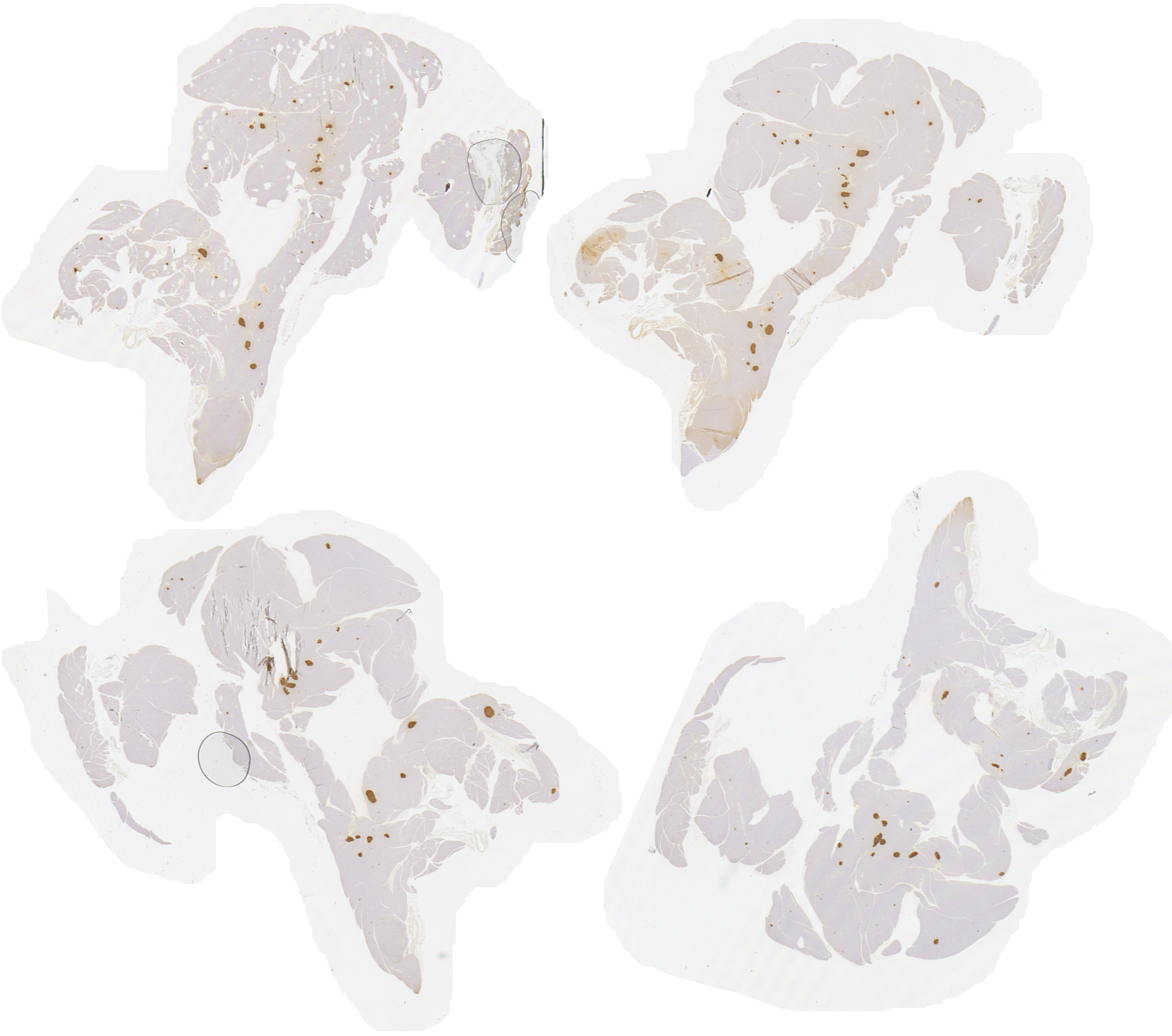

Supplement: Figure 1—source data 1. — The zip file contains all the 2400-dpi scanned images of insulin-immunostained pancreas sections used for quantitation of insulin-positive cell area. Insulin is stained as brown, and the whole section is counterstained as blue with hematoxylin. Folders are named after genotypes (P-Adn++ or P-Adn−−) and subfolders after time points (week 0, 2, 5, or 10) post initial dimerizer administration. Related to Figure 1A. DOI: http://dx.doi.org/10.7554/eLife.03851.004 [file elife03851s001.zip › Figure 1-source data 1/P-Adn--/Week 0/AKO25-6s c.jpg]

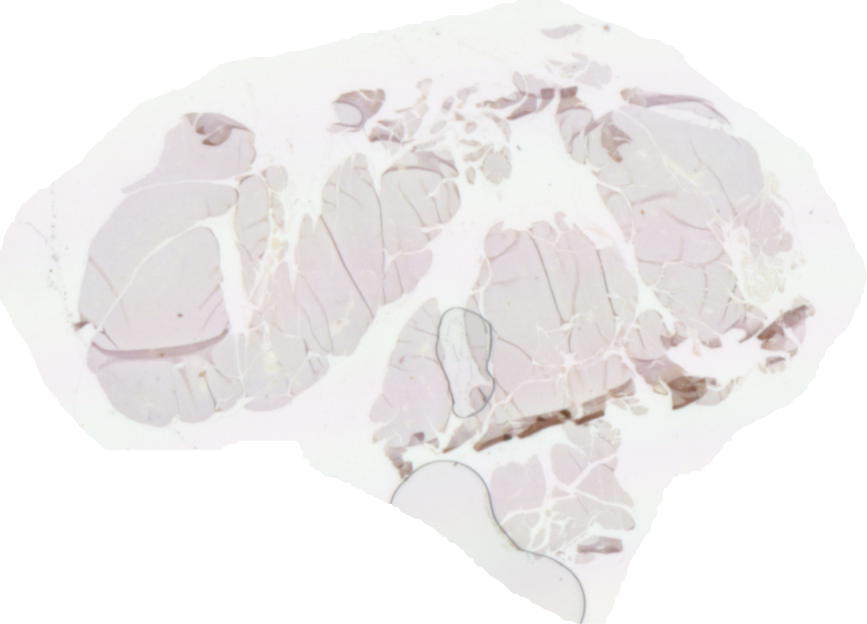

Supplement: Figure 1—source data 1. — The zip file contains all the 2400-dpi scanned images of insulin-immunostained pancreas sections used for quantitation of insulin-positive cell area. Insulin is stained as brown, and the whole section is counterstained as blue with hematoxylin. Folders are named after genotypes (P-Adn++ or P-Adn−−) and subfolders after time points (week 0, 2, 5, or 10) post initial dimerizer administration. Related to Figure 1A. DOI: http://dx.doi.org/10.7554/eLife.03851.004 [file elife03851s001.zip › Figure 1-source data 1/P-Adn--/Week 10/AKO25 a.jpg]

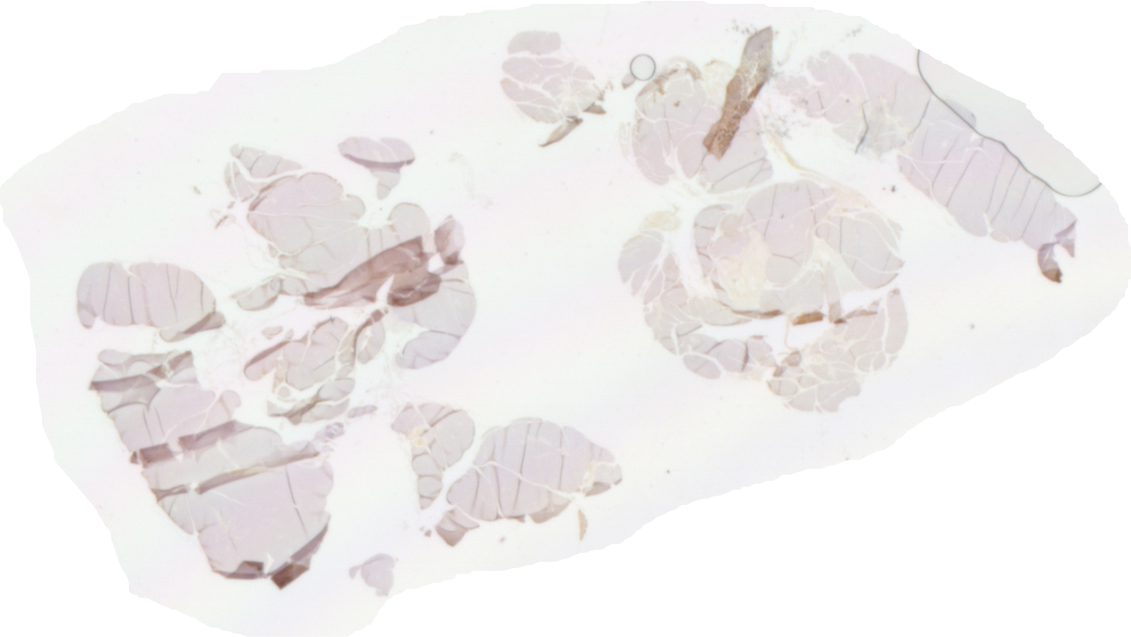

Supplement: Figure 1—source data 1. — The zip file contains all the 2400-dpi scanned images of insulin-immunostained pancreas sections used for quantitation of insulin-positive cell area. Insulin is stained as brown, and the whole section is counterstained as blue with hematoxylin. Folders are named after genotypes (P-Adn++ or P-Adn−−) and subfolders after time points (week 0, 2, 5, or 10) post initial dimerizer administration. Related to Figure 1A. DOI: http://dx.doi.org/10.7554/eLife.03851.004 [file elife03851s001.zip › Figure 1-source data 1/P-Adn--/Week 10/AKO25 b.jpg]

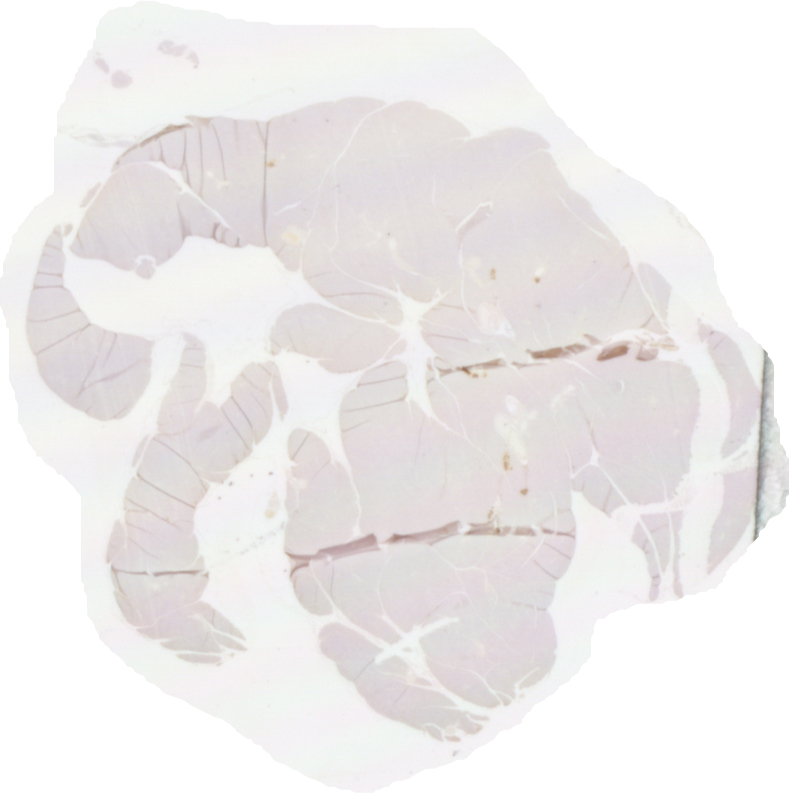

Supplement: Figure 1—source data 1. — The zip file contains all the 2400-dpi scanned images of insulin-immunostained pancreas sections used for quantitation of insulin-positive cell area. Insulin is stained as brown, and the whole section is counterstained as blue with hematoxylin. Folders are named after genotypes (P-Adn++ or P-Adn−−) and subfolders after time points (week 0, 2, 5, or 10) post initial dimerizer administration. Related to Figure 1A. DOI: http://dx.doi.org/10.7554/eLife.03851.004 [file elife03851s001.zip › Figure 1-source data 1/P-Adn--/Week 10/AKO25 c.jpg]

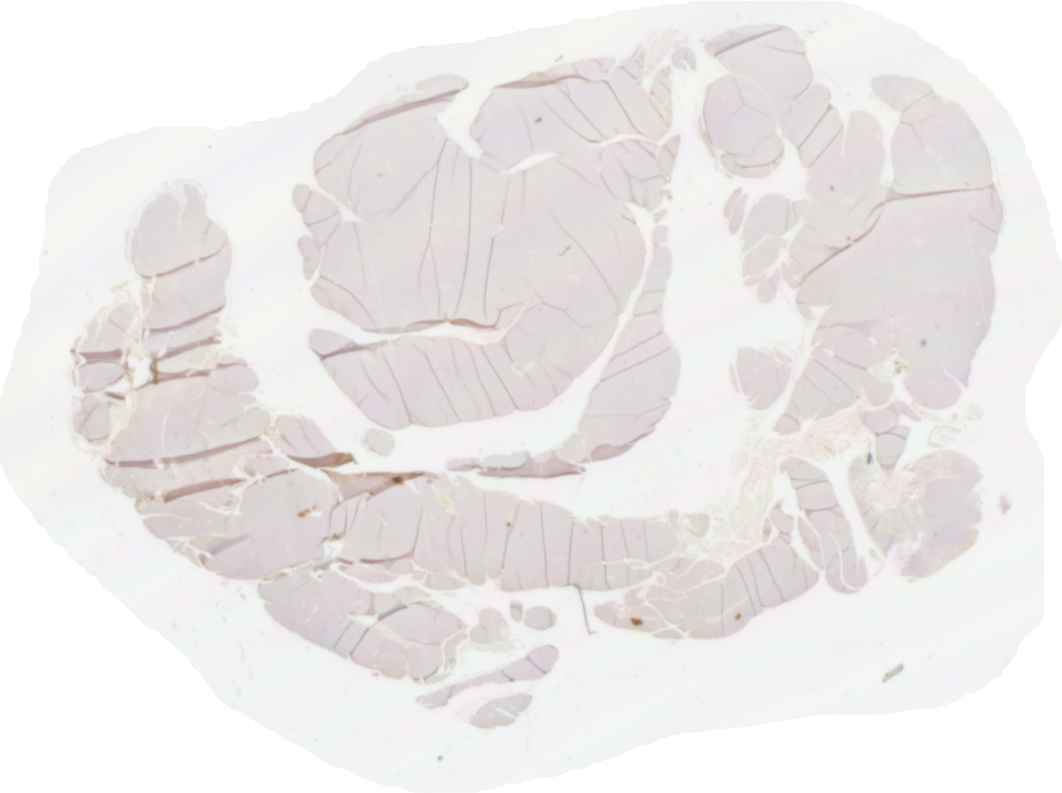

Supplement: Figure 1—source data 1. — The zip file contains all the 2400-dpi scanned images of insulin-immunostained pancreas sections used for quantitation of insulin-positive cell area. Insulin is stained as brown, and the whole section is counterstained as blue with hematoxylin. Folders are named after genotypes (P-Adn++ or P-Adn−−) and subfolders after time points (week 0, 2, 5, or 10) post initial dimerizer administration. Related to Figure 1A. DOI: http://dx.doi.org/10.7554/eLife.03851.004 [file elife03851s001.zip › Figure 1-source data 1/P-Adn--/Week 10/AKO25 d.jpg]

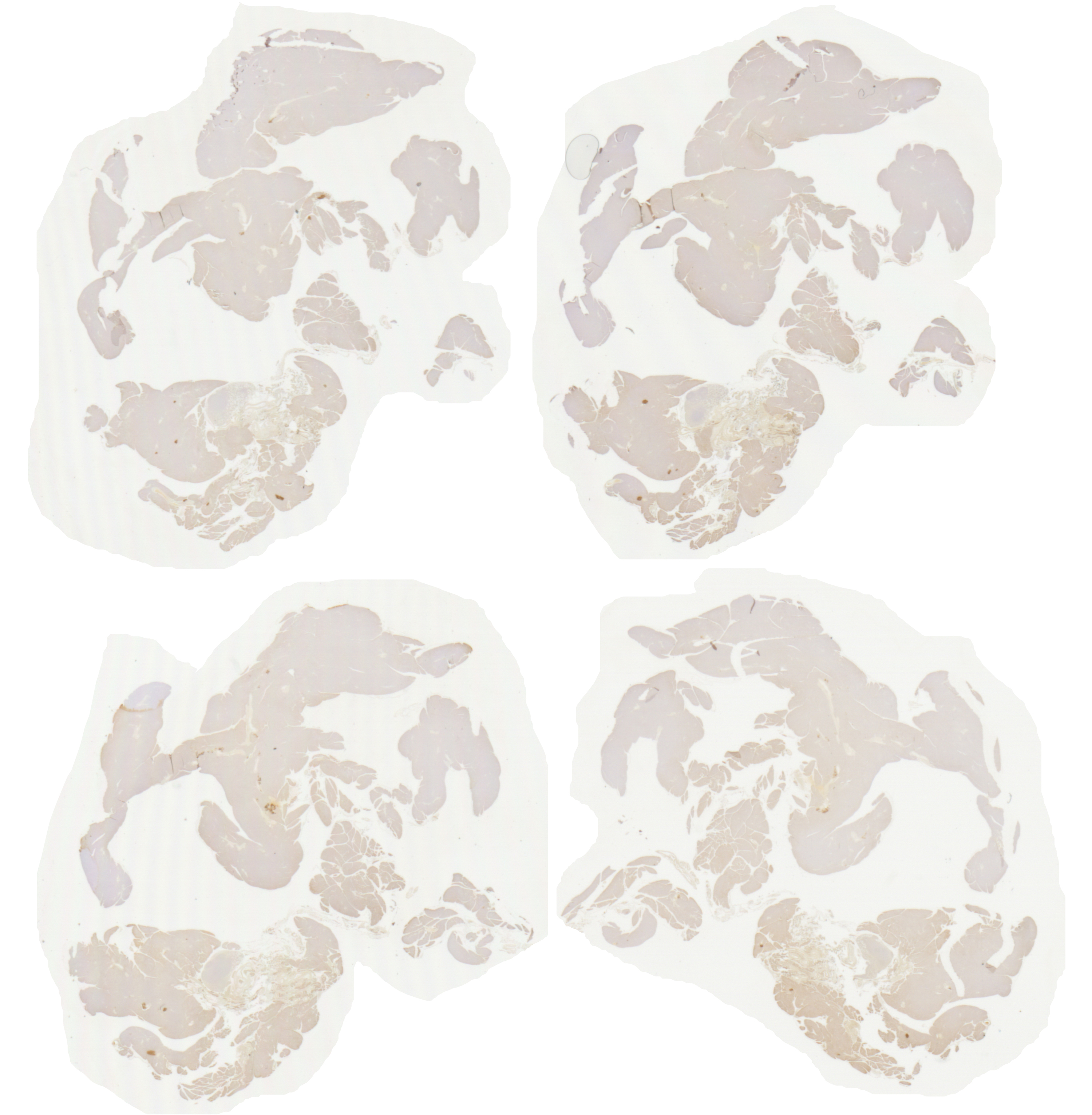

Supplement: Figure 1—source data 1. — The zip file contains all the 2400-dpi scanned images of insulin-immunostained pancreas sections used for quantitation of insulin-positive cell area. Insulin is stained as brown, and the whole section is counterstained as blue with hematoxylin. Folders are named after genotypes (P-Adn++ or P-Adn−−) and subfolders after time points (week 0, 2, 5, or 10) post initial dimerizer administration. Related to Figure 1A. DOI: http://dx.doi.org/10.7554/eLife.03851.004 [file elife03851s001.zip › Figure 1-source data 1/P-Adn--/Week 10/AKO42-5s a.jpg]

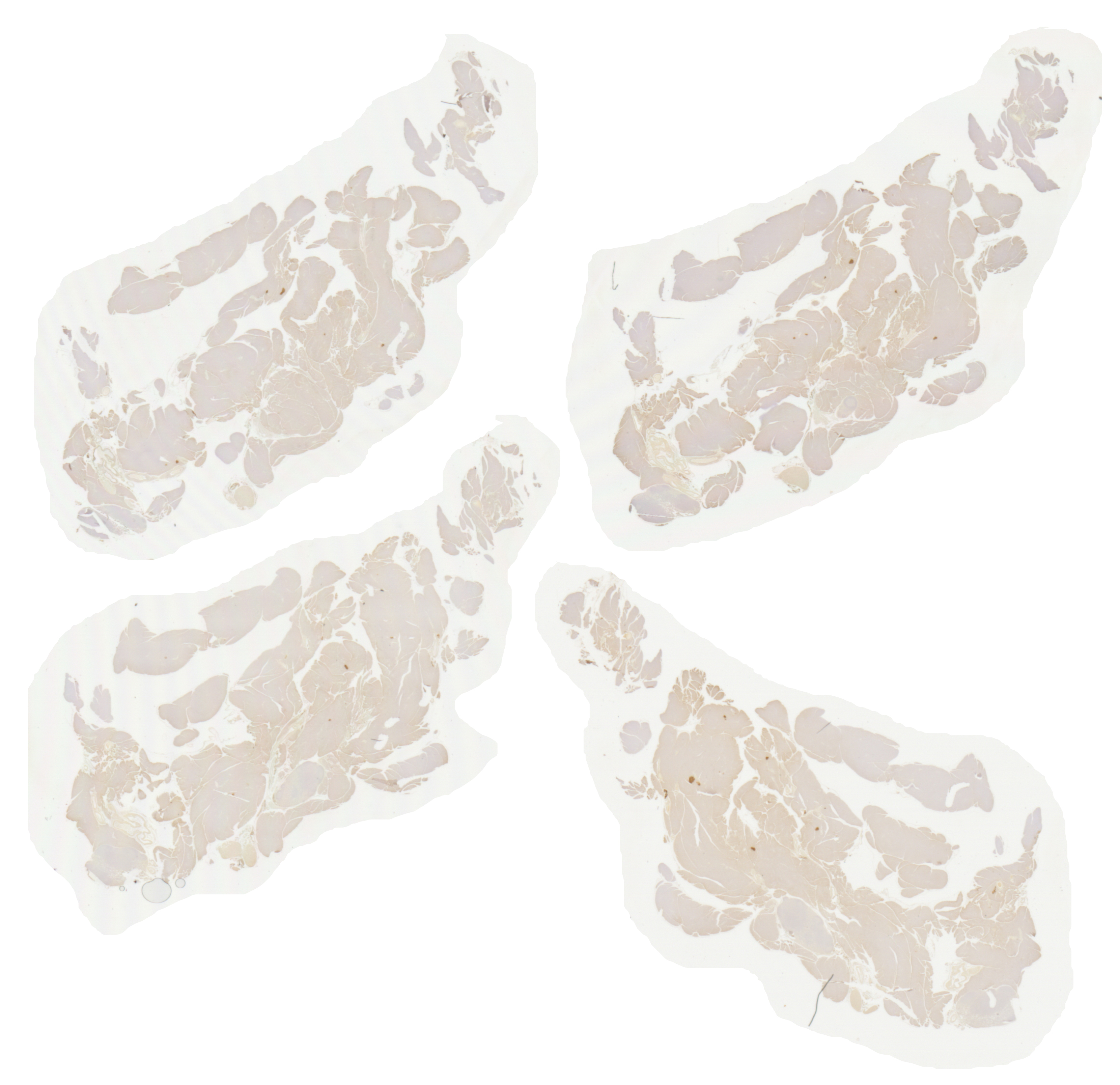

Supplement: Figure 1—source data 1. — The zip file contains all the 2400-dpi scanned images of insulin-immunostained pancreas sections used for quantitation of insulin-positive cell area. Insulin is stained as brown, and the whole section is counterstained as blue with hematoxylin. Folders are named after genotypes (P-Adn++ or P-Adn−−) and subfolders after time points (week 0, 2, 5, or 10) post initial dimerizer administration. Related to Figure 1A. DOI: http://dx.doi.org/10.7554/eLife.03851.004 [file elife03851s001.zip › Figure 1-source data 1/P-Adn--/Week 10/AKO42-5s b.jpg]

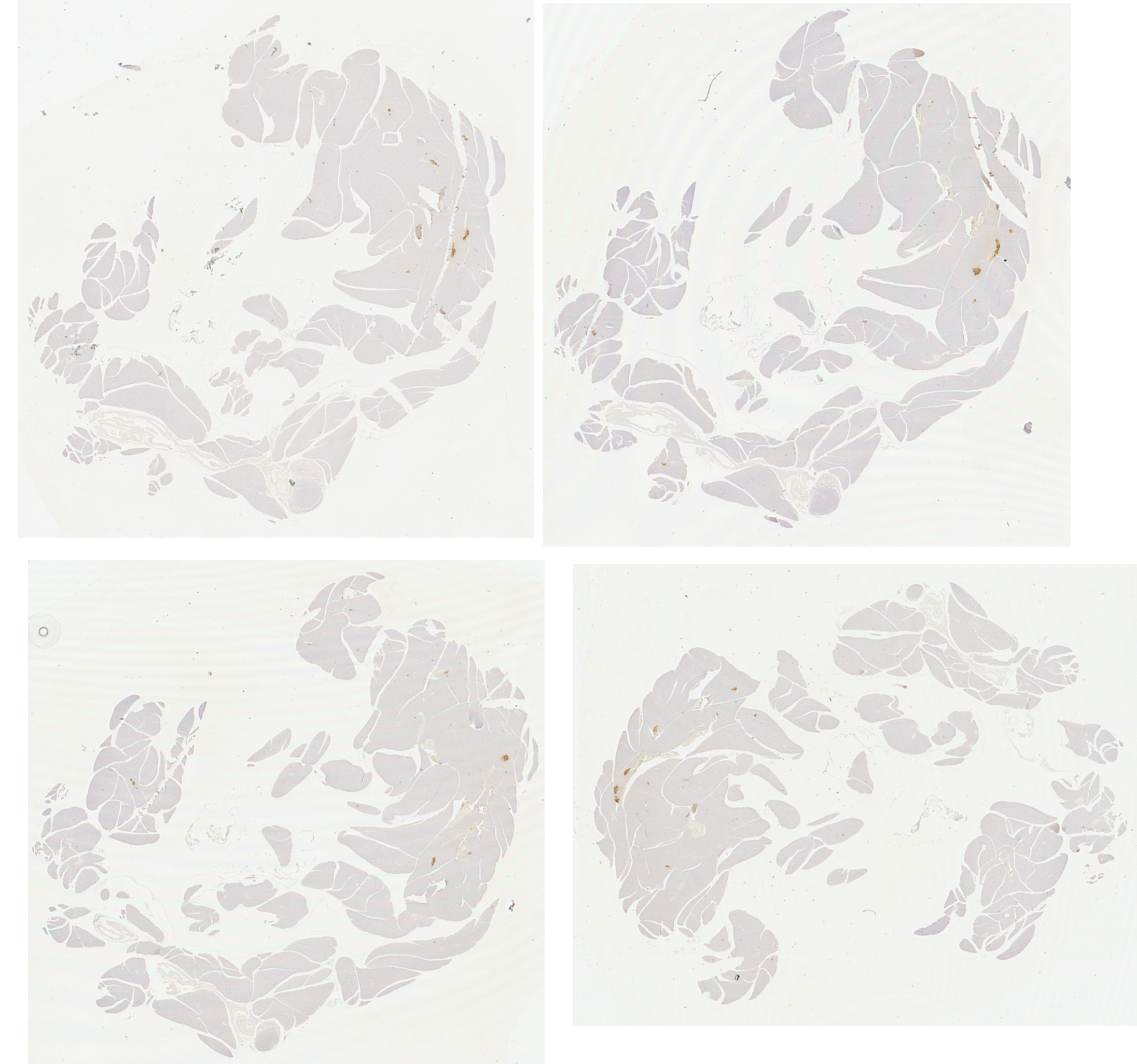

Supplement: Figure 1—source data 1. — The zip file contains all the 2400-dpi scanned images of insulin-immunostained pancreas sections used for quantitation of insulin-positive cell area. Insulin is stained as brown, and the whole section is counterstained as blue with hematoxylin. Folders are named after genotypes (P-Adn++ or P-Adn−−) and subfolders after time points (week 0, 2, 5, or 10) post initial dimerizer administration. Related to Figure 1A. DOI: http://dx.doi.org/10.7554/eLife.03851.004 [file elife03851s001.zip › Figure 1-source data 1/P-Adn--/Week 2/AKO30 a.jpg]

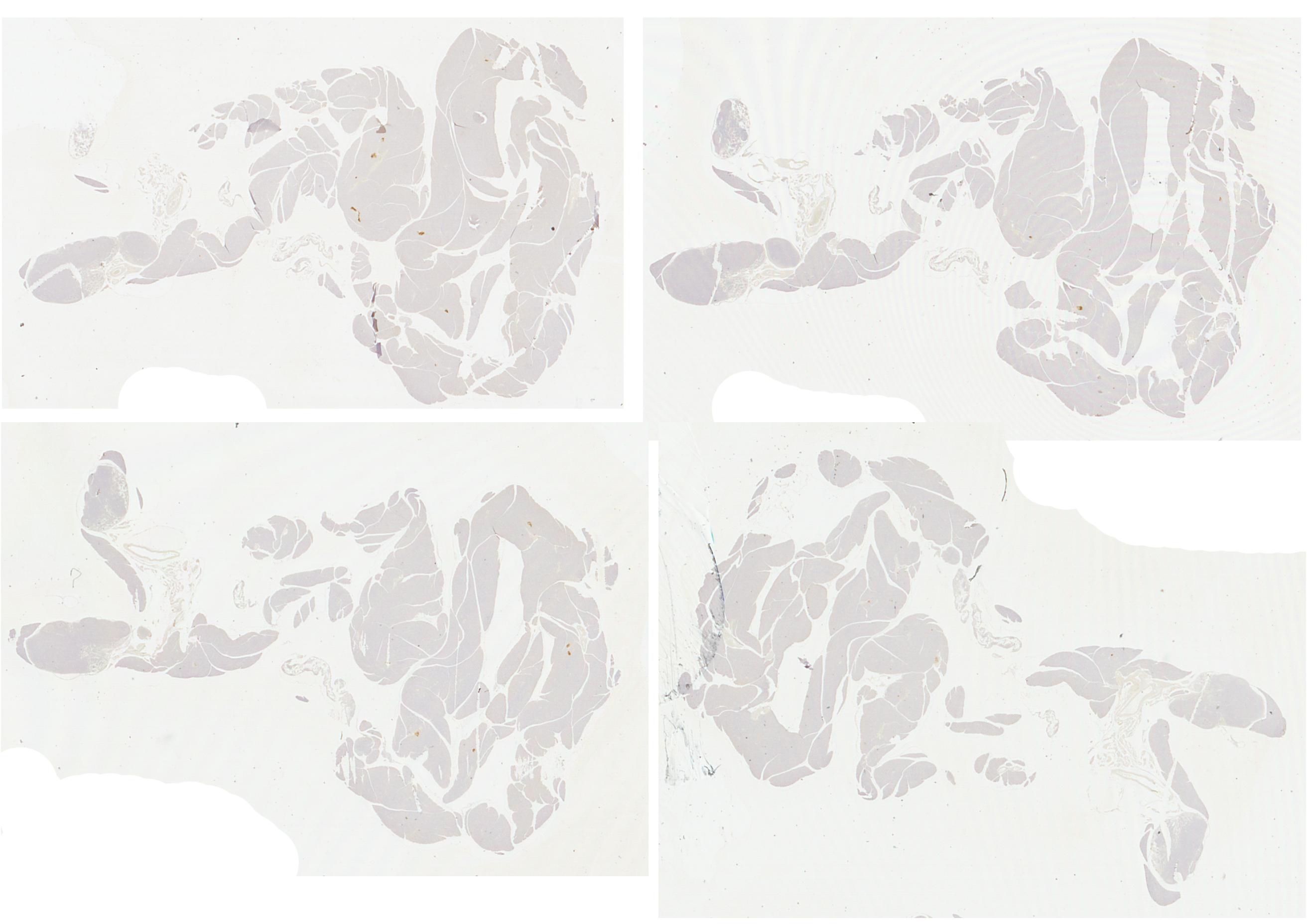

Supplement: Figure 1—source data 1. — The zip file contains all the 2400-dpi scanned images of insulin-immunostained pancreas sections used for quantitation of insulin-positive cell area. Insulin is stained as brown, and the whole section is counterstained as blue with hematoxylin. Folders are named after genotypes (P-Adn++ or P-Adn−−) and subfolders after time points (week 0, 2, 5, or 10) post initial dimerizer administration. Related to Figure 1A. DOI: http://dx.doi.org/10.7554/eLife.03851.004 [file elife03851s001.zip › Figure 1-source data 1/P-Adn--/Week 2/AKO30 b.jpg]

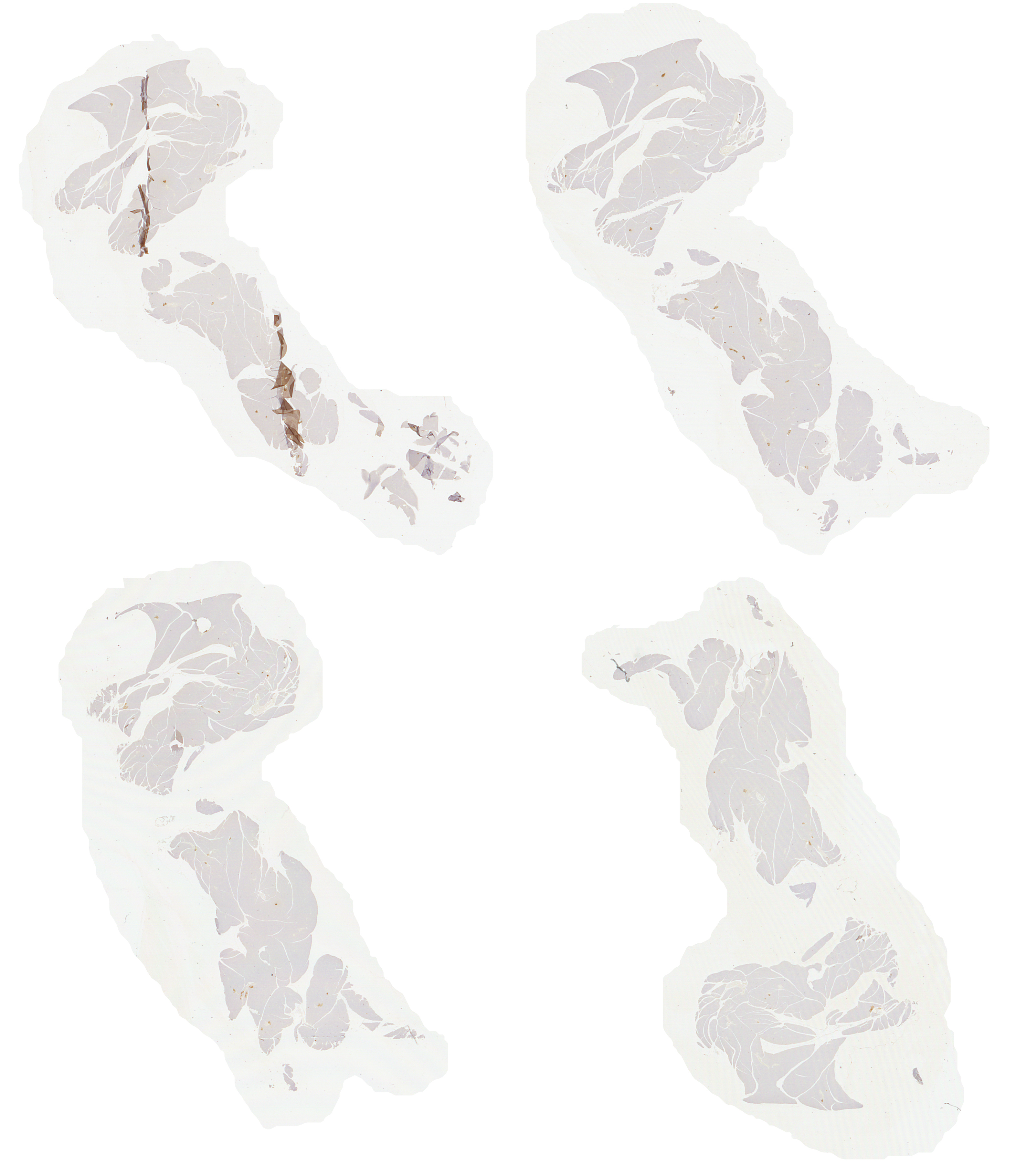

Supplement: Figure 1—source data 1. — The zip file contains all the 2400-dpi scanned images of insulin-immunostained pancreas sections used for quantitation of insulin-positive cell area. Insulin is stained as brown, and the whole section is counterstained as blue with hematoxylin. Folders are named after genotypes (P-Adn++ or P-Adn−−) and subfolders after time points (week 0, 2, 5, or 10) post initial dimerizer administration. Related to Figure 1A. DOI: http://dx.doi.org/10.7554/eLife.03851.004 [file elife03851s001.zip › Figure 1-source data 1/P-Adn--/Week 2/AKO30 c.jpg]

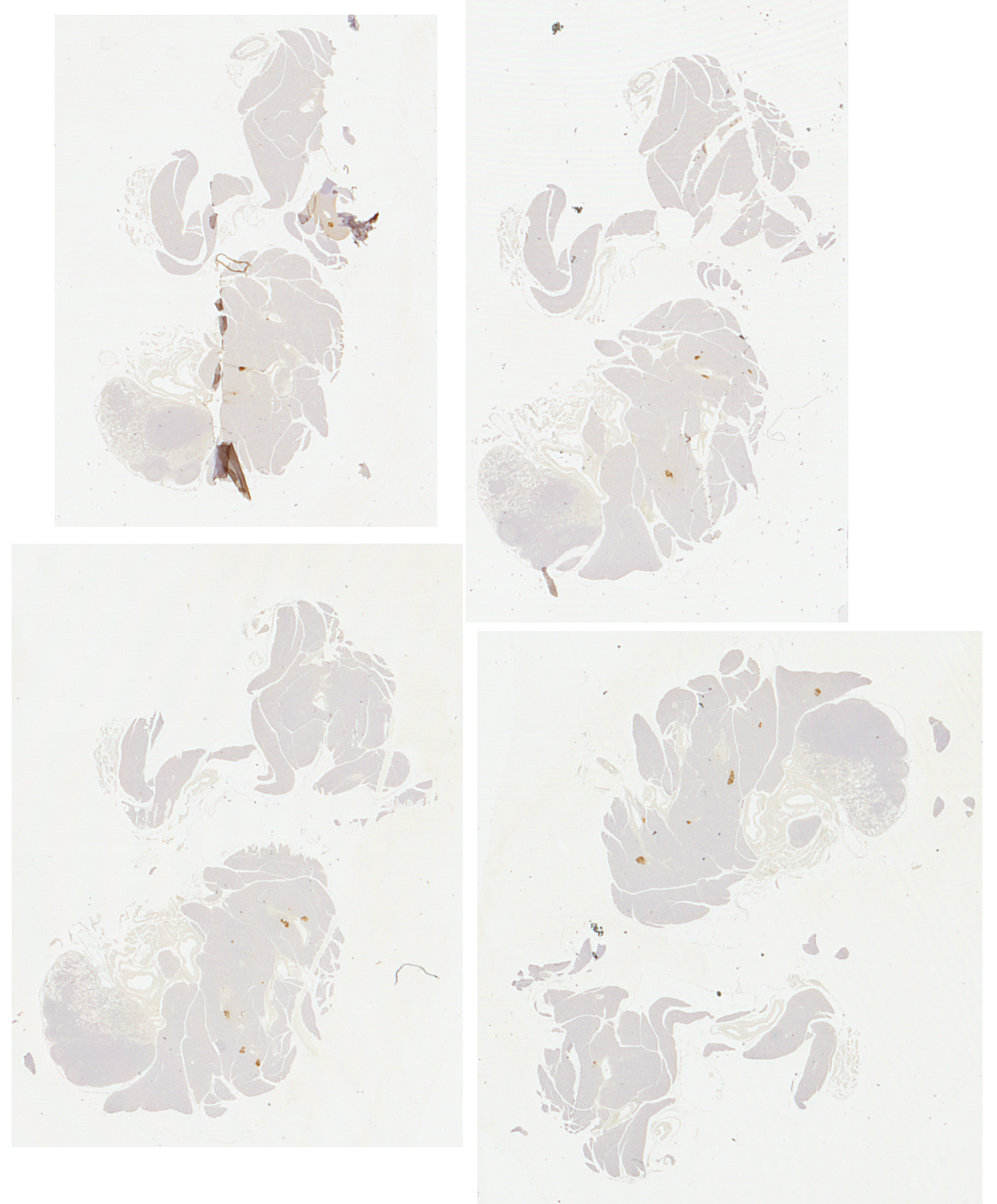

Supplement: Figure 1—source data 1. — The zip file contains all the 2400-dpi scanned images of insulin-immunostained pancreas sections used for quantitation of insulin-positive cell area. Insulin is stained as brown, and the whole section is counterstained as blue with hematoxylin. Folders are named after genotypes (P-Adn++ or P-Adn−−) and subfolders after time points (week 0, 2, 5, or 10) post initial dimerizer administration. Related to Figure 1A. DOI: http://dx.doi.org/10.7554/eLife.03851.004 [file elife03851s001.zip › Figure 1-source data 1/P-Adn--/Week 2/AKO30 d.jpg]

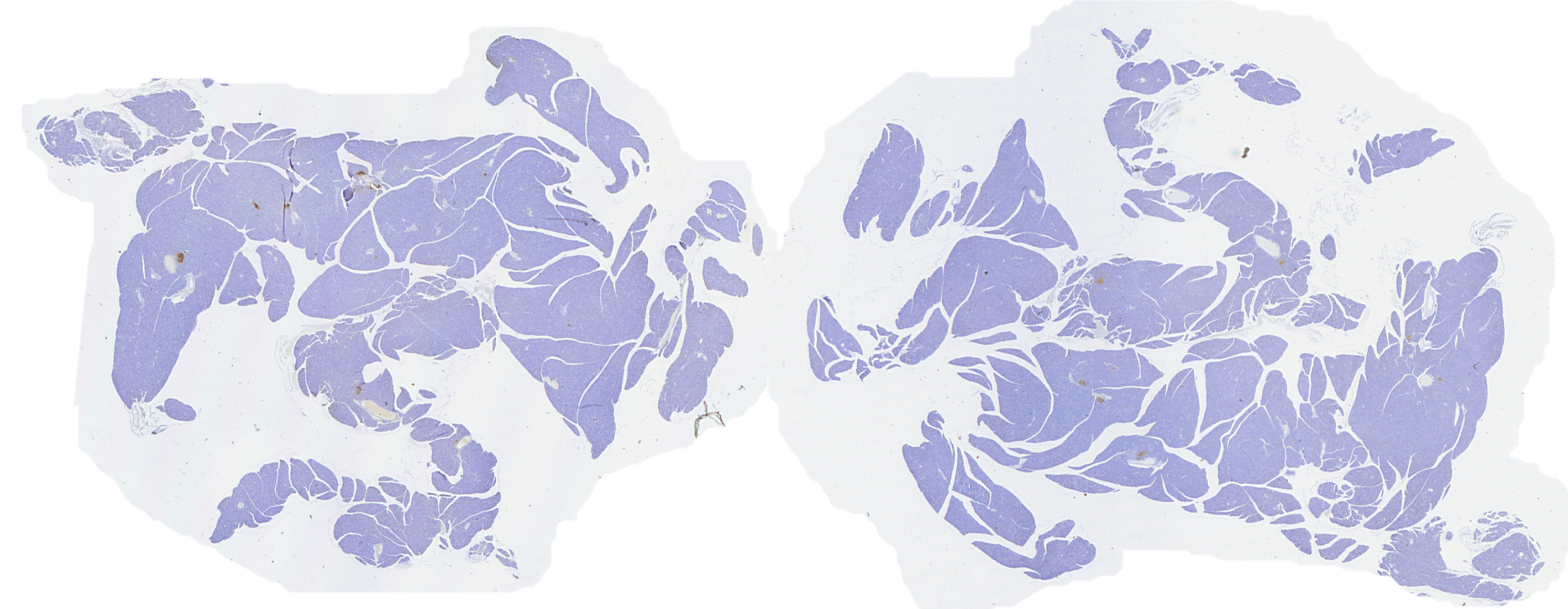

Supplement: Figure 1—source data 1. — The zip file contains all the 2400-dpi scanned images of insulin-immunostained pancreas sections used for quantitation of insulin-positive cell area. Insulin is stained as brown, and the whole section is counterstained as blue with hematoxylin. Folders are named after genotypes (P-Adn++ or P-Adn−−) and subfolders after time points (week 0, 2, 5, or 10) post initial dimerizer administration. Related to Figure 1A. DOI: http://dx.doi.org/10.7554/eLife.03851.004 [file elife03851s001.zip › Figure 1-source data 1/P-Adn--/Week 5/AKO42-1s a.jpg]

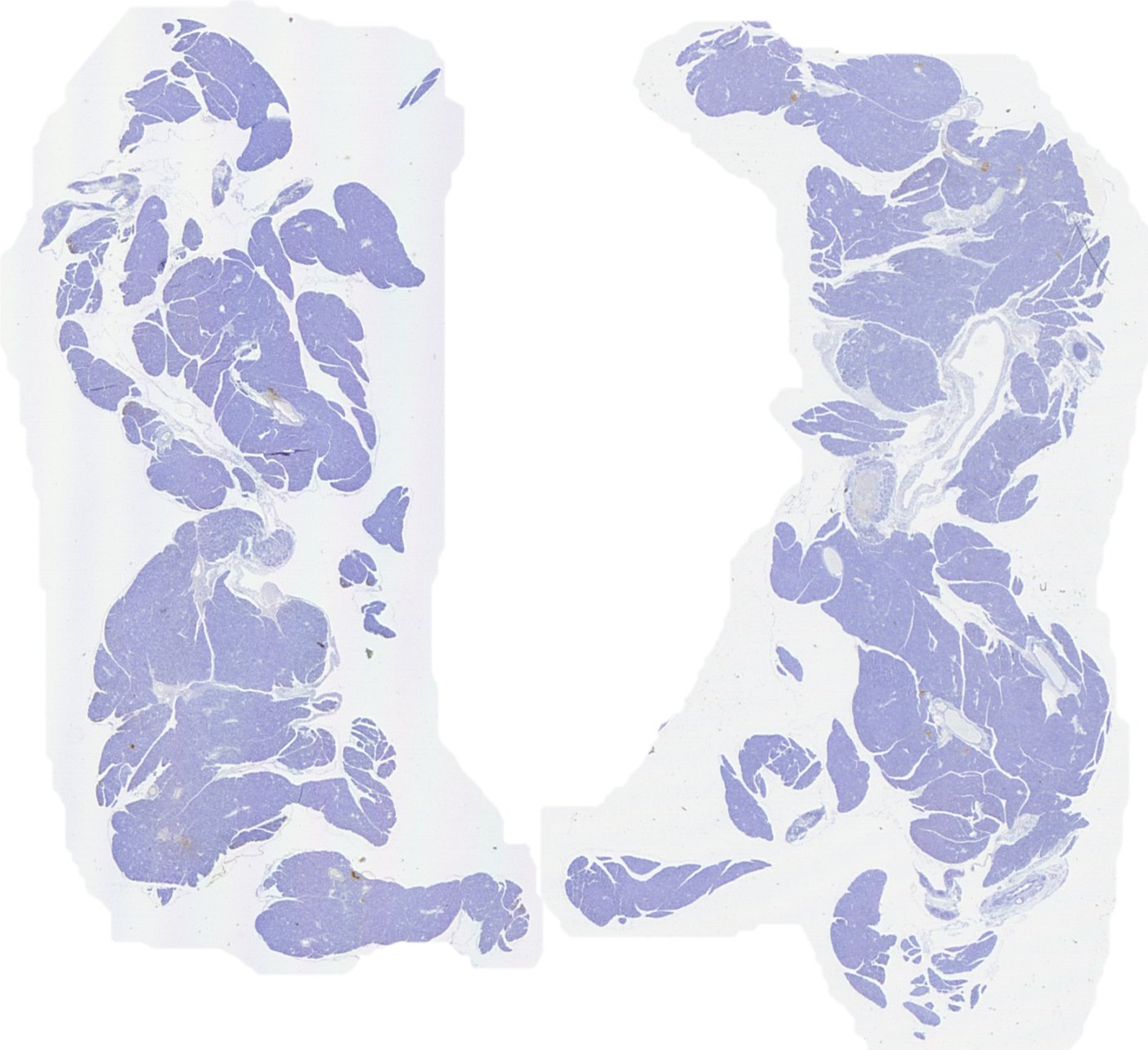

Supplement: Figure 1—source data 1. — The zip file contains all the 2400-dpi scanned images of insulin-immunostained pancreas sections used for quantitation of insulin-positive cell area. Insulin is stained as brown, and the whole section is counterstained as blue with hematoxylin. Folders are named after genotypes (P-Adn++ or P-Adn−−) and subfolders after time points (week 0, 2, 5, or 10) post initial dimerizer administration. Related to Figure 1A. DOI: http://dx.doi.org/10.7554/eLife.03851.004 [file elife03851s001.zip › Figure 1-source data 1/P-Adn--/Week 5/AKO42-1s b.jpg]

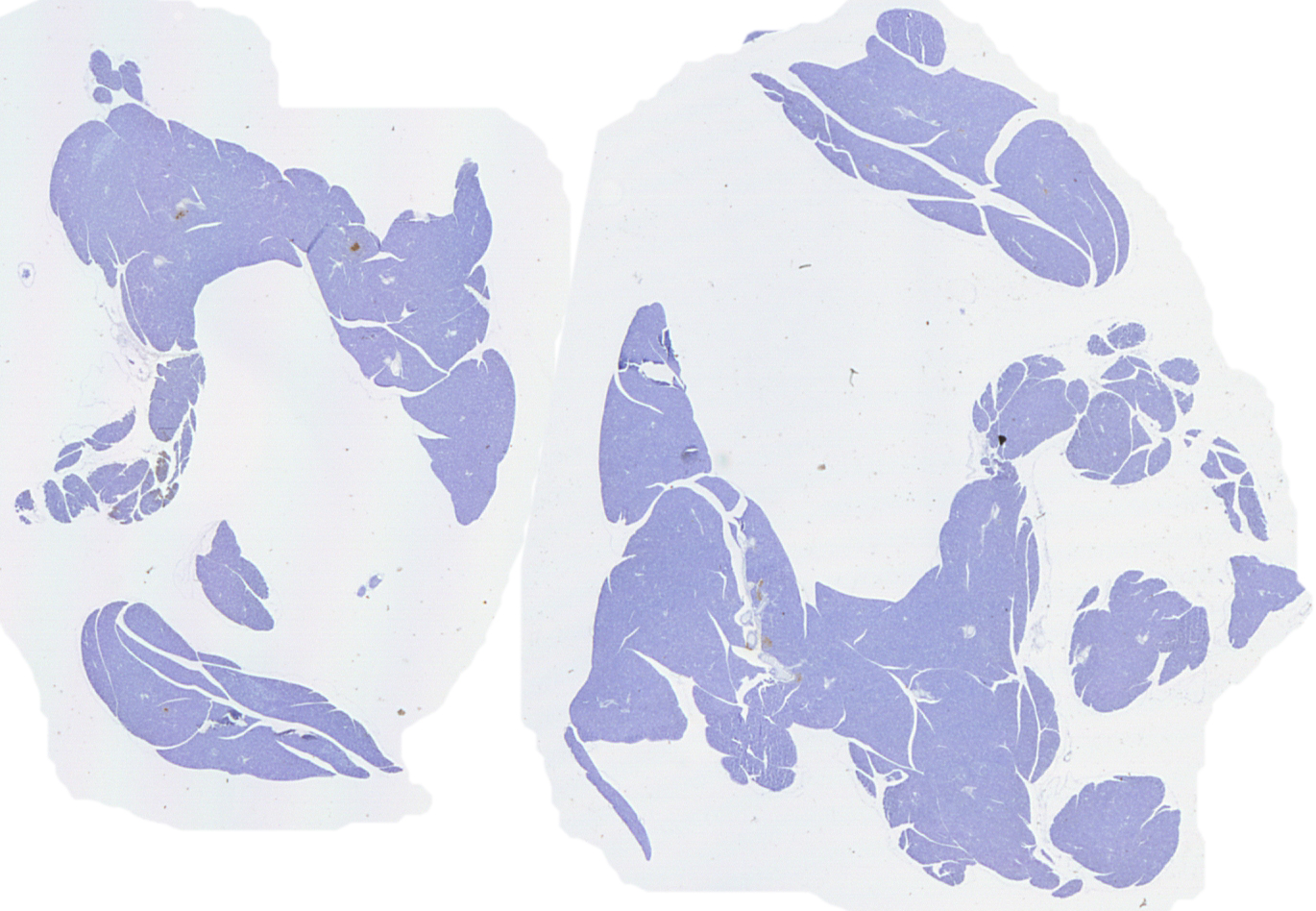

Supplement: Figure 1—source data 1. — The zip file contains all the 2400-dpi scanned images of insulin-immunostained pancreas sections used for quantitation of insulin-positive cell area. Insulin is stained as brown, and the whole section is counterstained as blue with hematoxylin. Folders are named after genotypes (P-Adn++ or P-Adn−−) and subfolders after time points (week 0, 2, 5, or 10) post initial dimerizer administration. Related to Figure 1A. DOI: http://dx.doi.org/10.7554/eLife.03851.004 [file elife03851s001.zip › Figure 1-source data 1/P-Adn--/Week 5/AKO42-1s c.jpg]

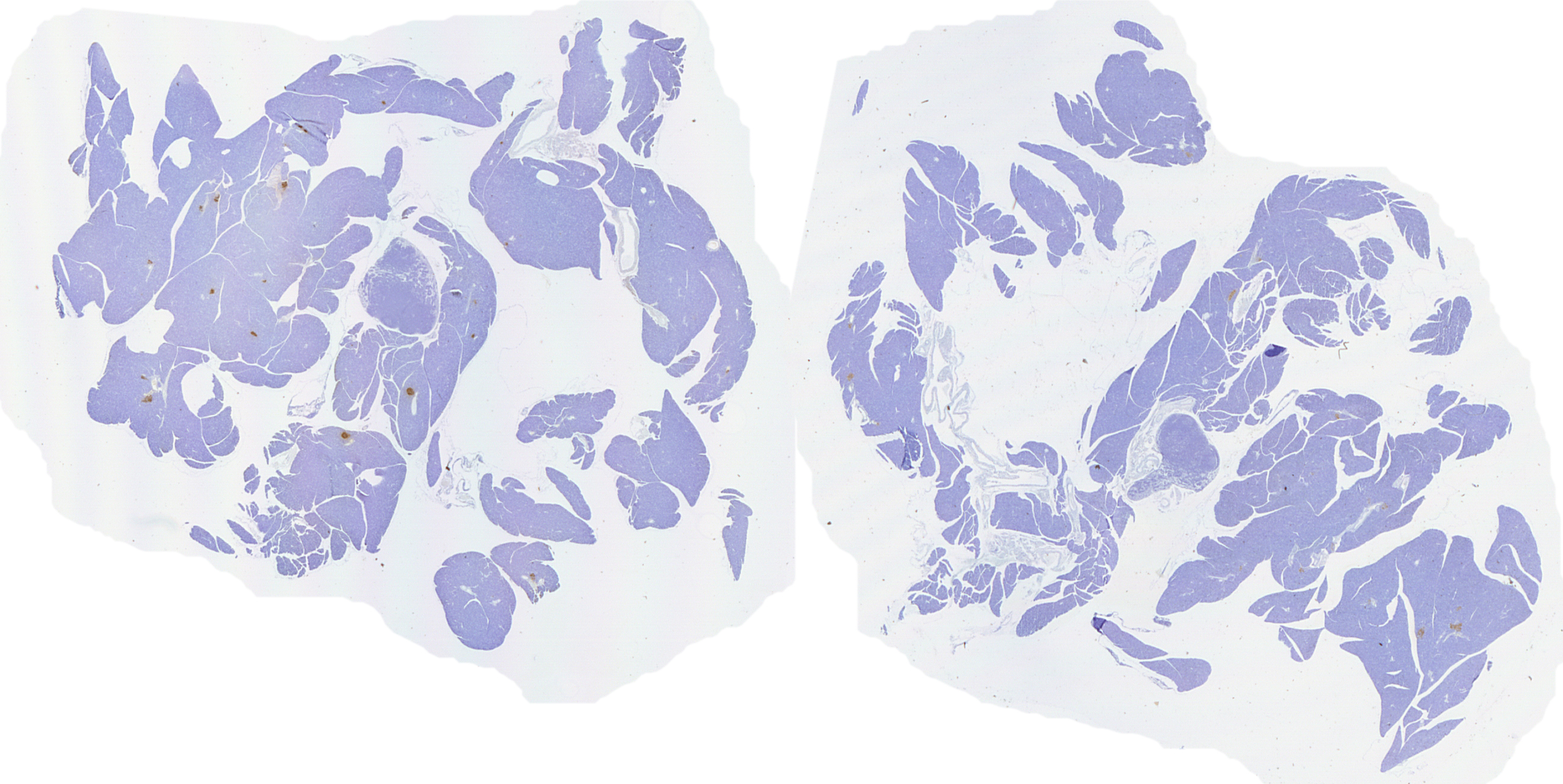

Supplement: Figure 1—source data 1. — The zip file contains all the 2400-dpi scanned images of insulin-immunostained pancreas sections used for quantitation of insulin-positive cell area. Insulin is stained as brown, and the whole section is counterstained as blue with hematoxylin. Folders are named after genotypes (P-Adn++ or P-Adn−−) and subfolders after time points (week 0, 2, 5, or 10) post initial dimerizer administration. Related to Figure 1A. DOI: http://dx.doi.org/10.7554/eLife.03851.004 [file elife03851s001.zip › Figure 1-source data 1/P-Adn--/Week 5/AKO42-1s d.jpg]

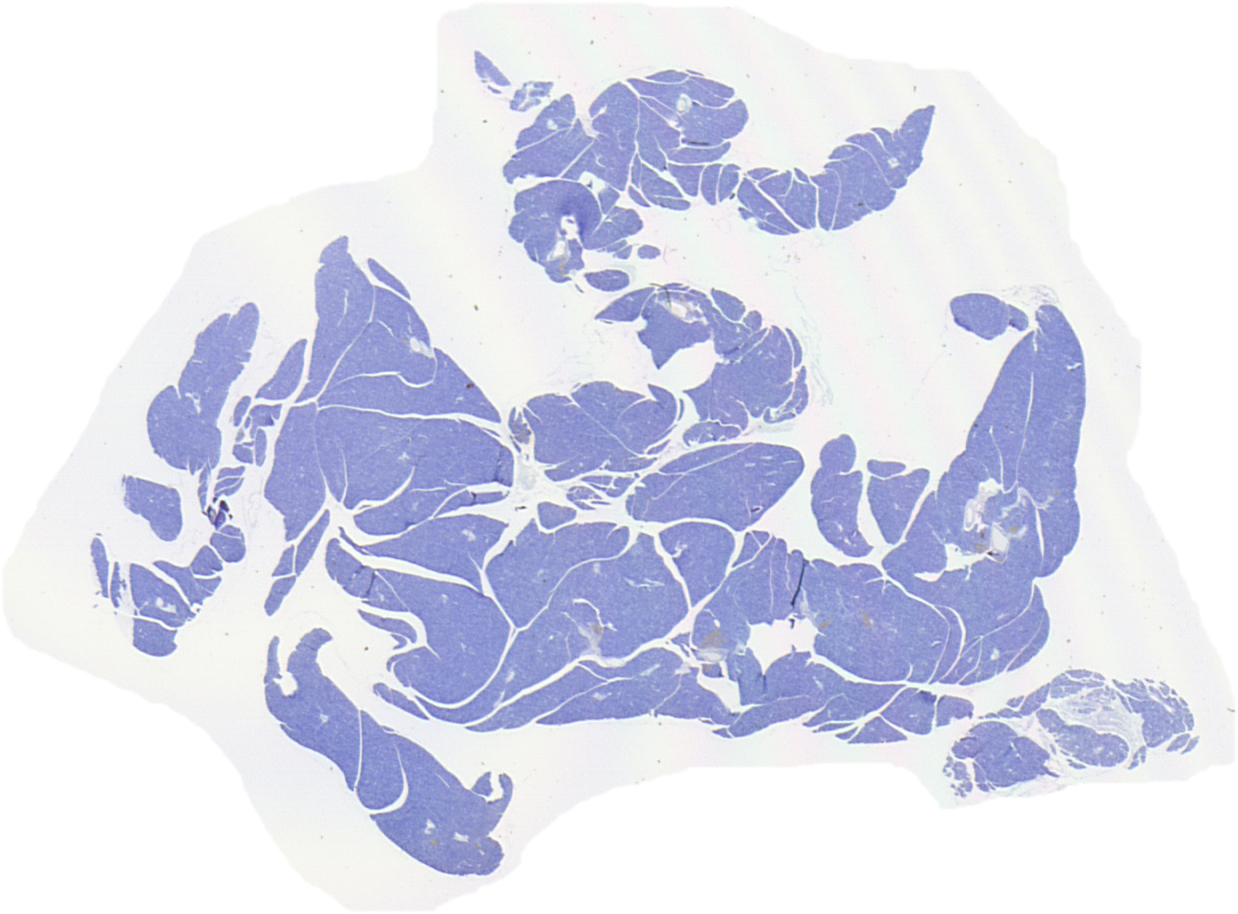

Supplement: Figure 1—source data 1. — The zip file contains all the 2400-dpi scanned images of insulin-immunostained pancreas sections used for quantitation of insulin-positive cell area. Insulin is stained as brown, and the whole section is counterstained as blue with hematoxylin. Folders are named after genotypes (P-Adn++ or P-Adn−−) and subfolders after time points (week 0, 2, 5, or 10) post initial dimerizer administration. Related to Figure 1A. DOI: http://dx.doi.org/10.7554/eLife.03851.004 [file elife03851s001.zip › Figure 1-source data 1/P-Adn--/Week 5/AKO42-1s-B1 a.jpg]

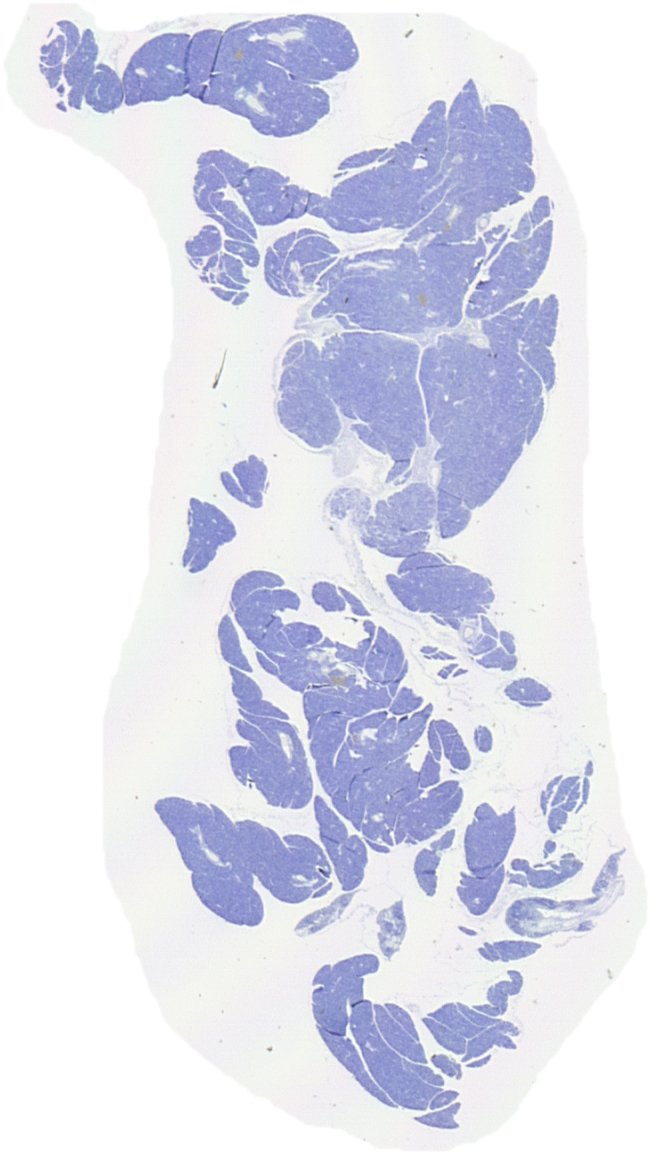

Supplement: Figure 1—source data 1. — The zip file contains all the 2400-dpi scanned images of insulin-immunostained pancreas sections used for quantitation of insulin-positive cell area. Insulin is stained as brown, and the whole section is counterstained as blue with hematoxylin. Folders are named after genotypes (P-Adn++ or P-Adn−−) and subfolders after time points (week 0, 2, 5, or 10) post initial dimerizer administration. Related to Figure 1A. DOI: http://dx.doi.org/10.7554/eLife.03851.004 [file elife03851s001.zip › Figure 1-source data 1/P-Adn--/Week 5/AKO42-1s-B1 b.jpg]

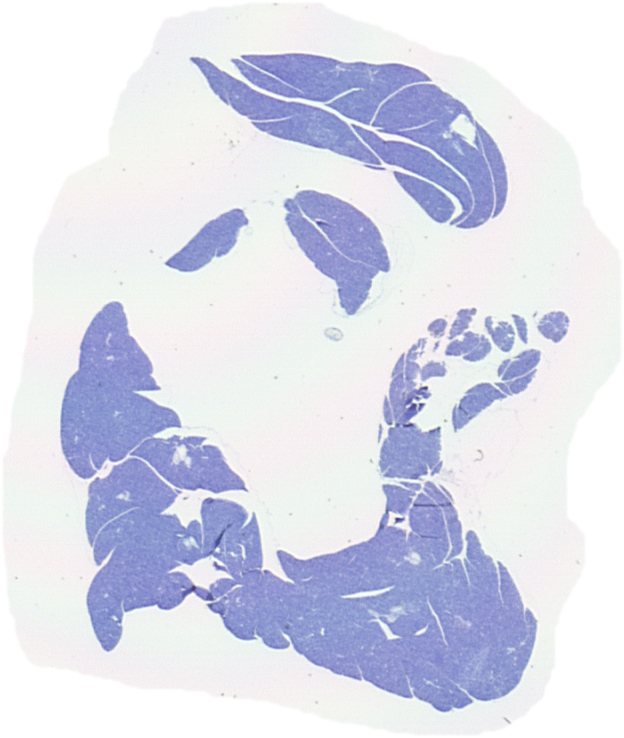

Supplement: Figure 1—source data 1. — The zip file contains all the 2400-dpi scanned images of insulin-immunostained pancreas sections used for quantitation of insulin-positive cell area. Insulin is stained as brown, and the whole section is counterstained as blue with hematoxylin. Folders are named after genotypes (P-Adn++ or P-Adn−−) and subfolders after time points (week 0, 2, 5, or 10) post initial dimerizer administration. Related to Figure 1A. DOI: http://dx.doi.org/10.7554/eLife.03851.004 [file elife03851s001.zip › Figure 1-source data 1/P-Adn--/Week 5/AKO42-1s-B1 c.jpg]

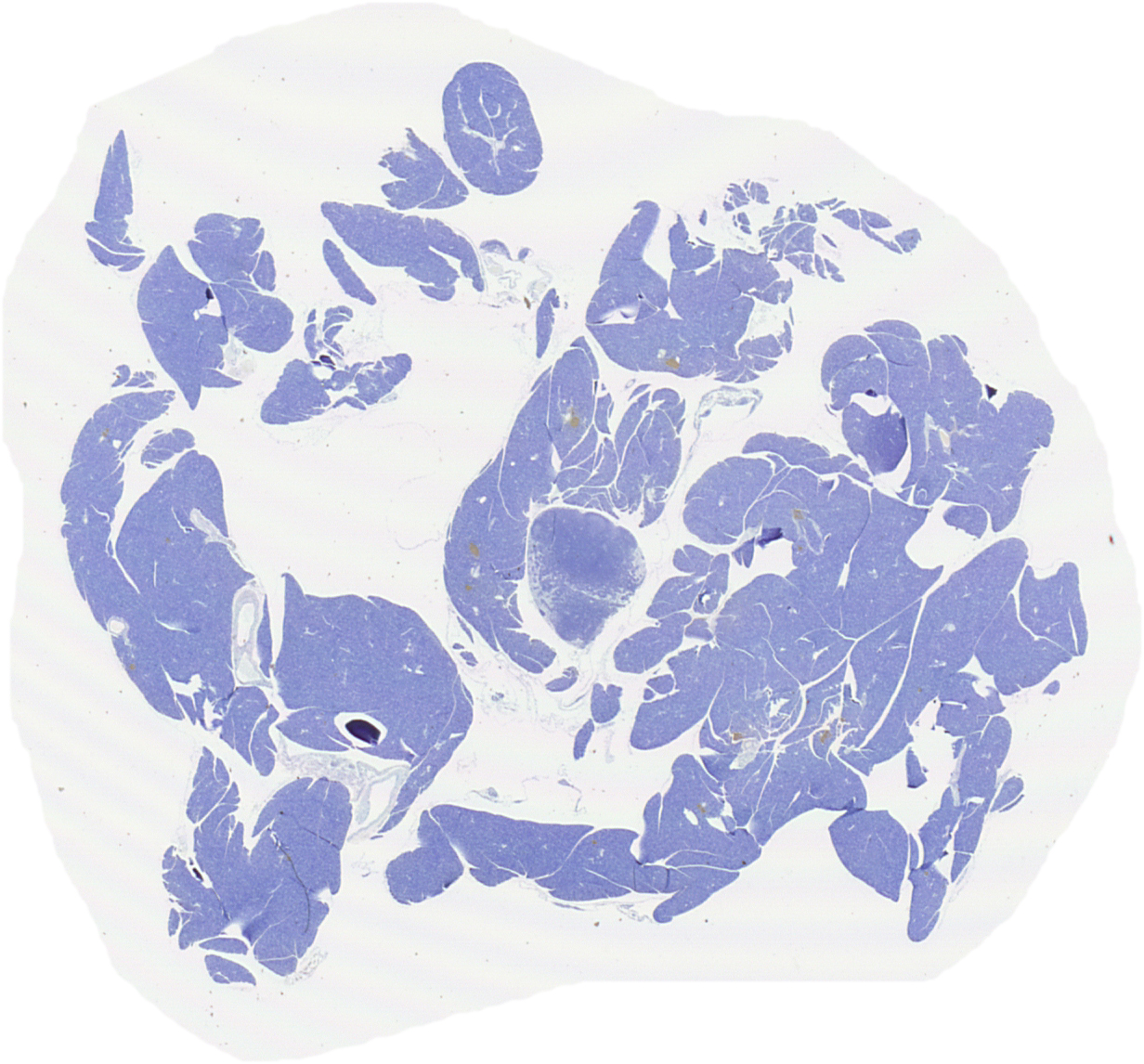

Supplement: Figure 1—source data 1. — The zip file contains all the 2400-dpi scanned images of insulin-immunostained pancreas sections used for quantitation of insulin-positive cell area. Insulin is stained as brown, and the whole section is counterstained as blue with hematoxylin. Folders are named after genotypes (P-Adn++ or P-Adn−−) and subfolders after time points (week 0, 2, 5, or 10) post initial dimerizer administration. Related to Figure 1A. DOI: http://dx.doi.org/10.7554/eLife.03851.004 [file elife03851s001.zip › Figure 1-source data 1/P-Adn--/Week 5/AKO42-1s-B1 d.jpg]

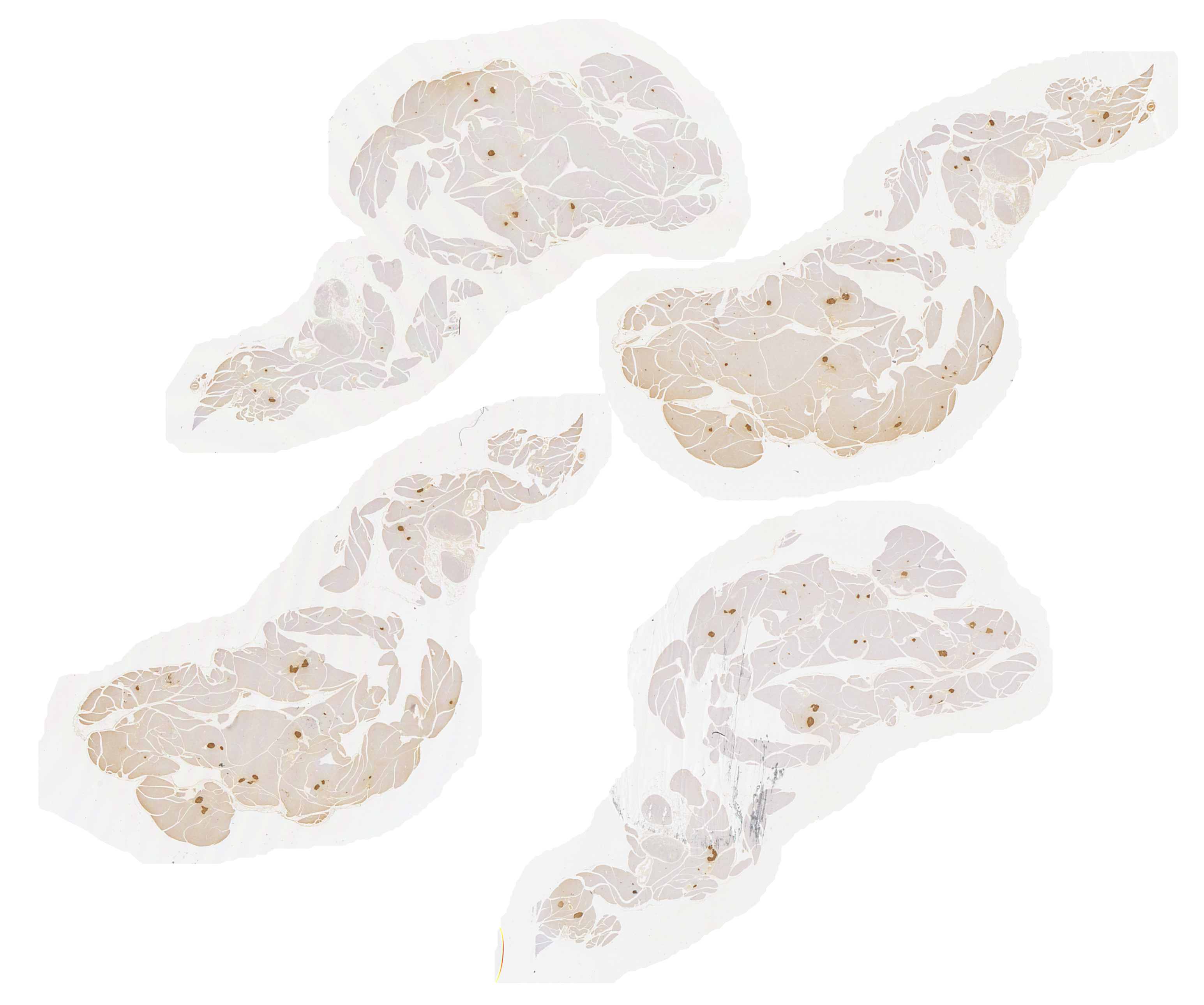

Supplement: Figure 1—source data 1. — The zip file contains all the 2400-dpi scanned images of insulin-immunostained pancreas sections used for quantitation of insulin-positive cell area. Insulin is stained as brown, and the whole section is counterstained as blue with hematoxylin. Folders are named after genotypes (P-Adn++ or P-Adn−−) and subfolders after time points (week 0, 2, 5, or 10) post initial dimerizer administration. Related to Figure 1A. DOI: http://dx.doi.org/10.7554/eLife.03851.004 [file elife03851s001.zip › Figure 1-source data 1/P-Adn++/Week 0/d47-2s a.jpg]

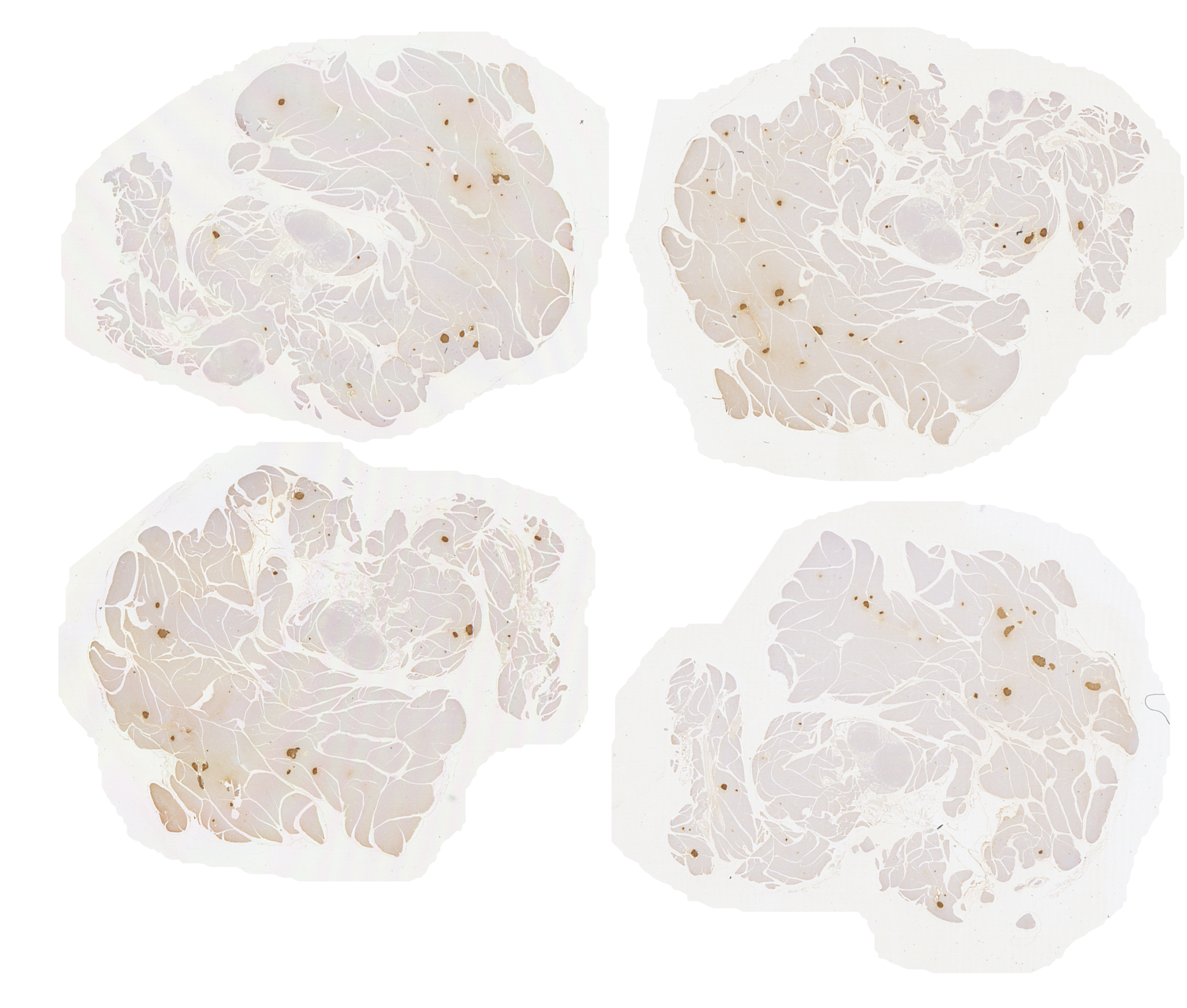

Supplement: Figure 1—source data 1. — The zip file contains all the 2400-dpi scanned images of insulin-immunostained pancreas sections used for quantitation of insulin-positive cell area. Insulin is stained as brown, and the whole section is counterstained as blue with hematoxylin. Folders are named after genotypes (P-Adn++ or P-Adn−−) and subfolders after time points (week 0, 2, 5, or 10) post initial dimerizer administration. Related to Figure 1A. DOI: http://dx.doi.org/10.7554/eLife.03851.004 [file elife03851s001.zip › Figure 1-source data 1/P-Adn++/Week 0/d47-2s b.jpg]

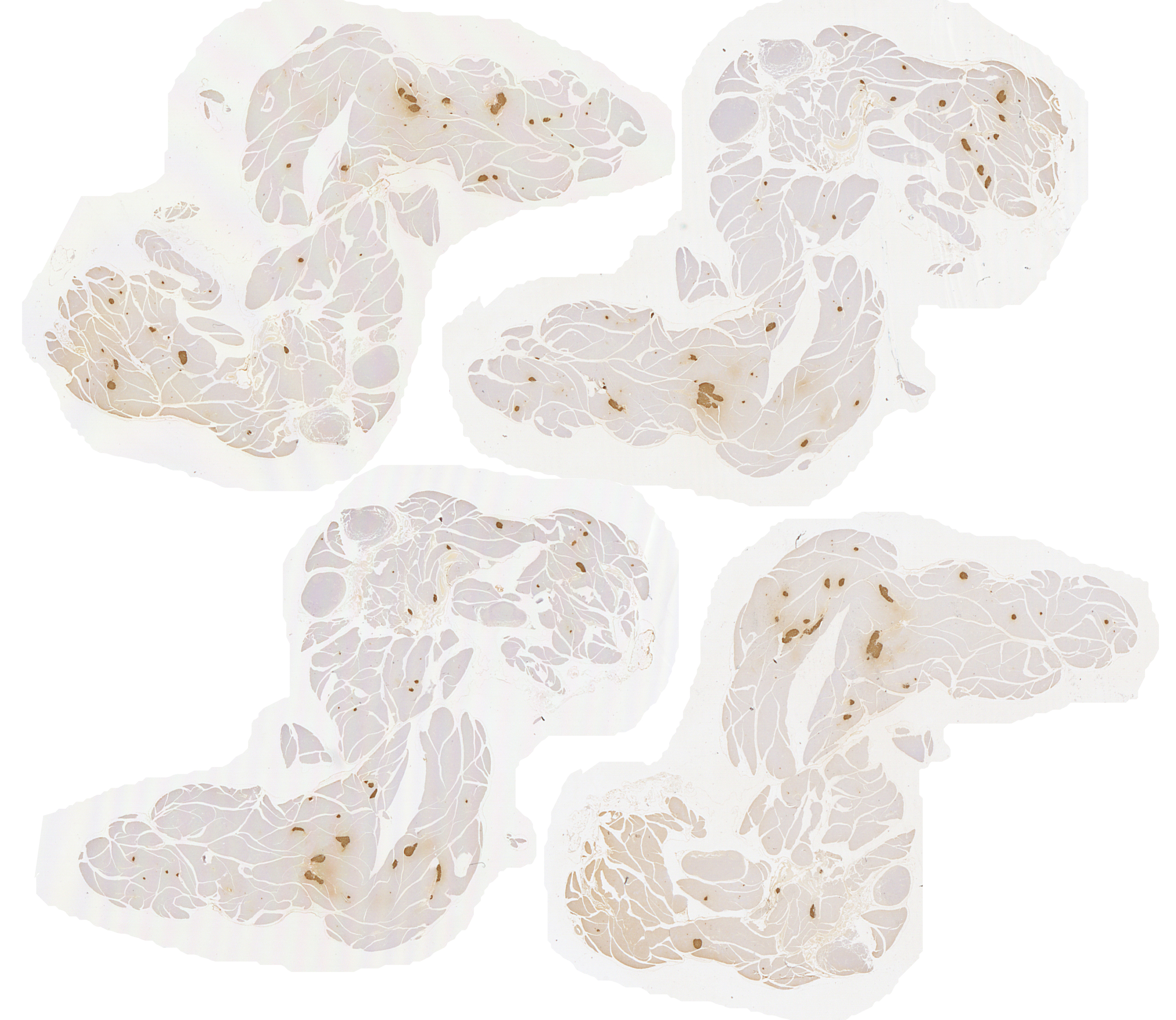

Supplement: Figure 1—source data 1. — The zip file contains all the 2400-dpi scanned images of insulin-immunostained pancreas sections used for quantitation of insulin-positive cell area. Insulin is stained as brown, and the whole section is counterstained as blue with hematoxylin. Folders are named after genotypes (P-Adn++ or P-Adn−−) and subfolders after time points (week 0, 2, 5, or 10) post initial dimerizer administration. Related to Figure 1A. DOI: http://dx.doi.org/10.7554/eLife.03851.004 [file elife03851s001.zip › Figure 1-source data 1/P-Adn++/Week 0/d47-2s c.jpg]

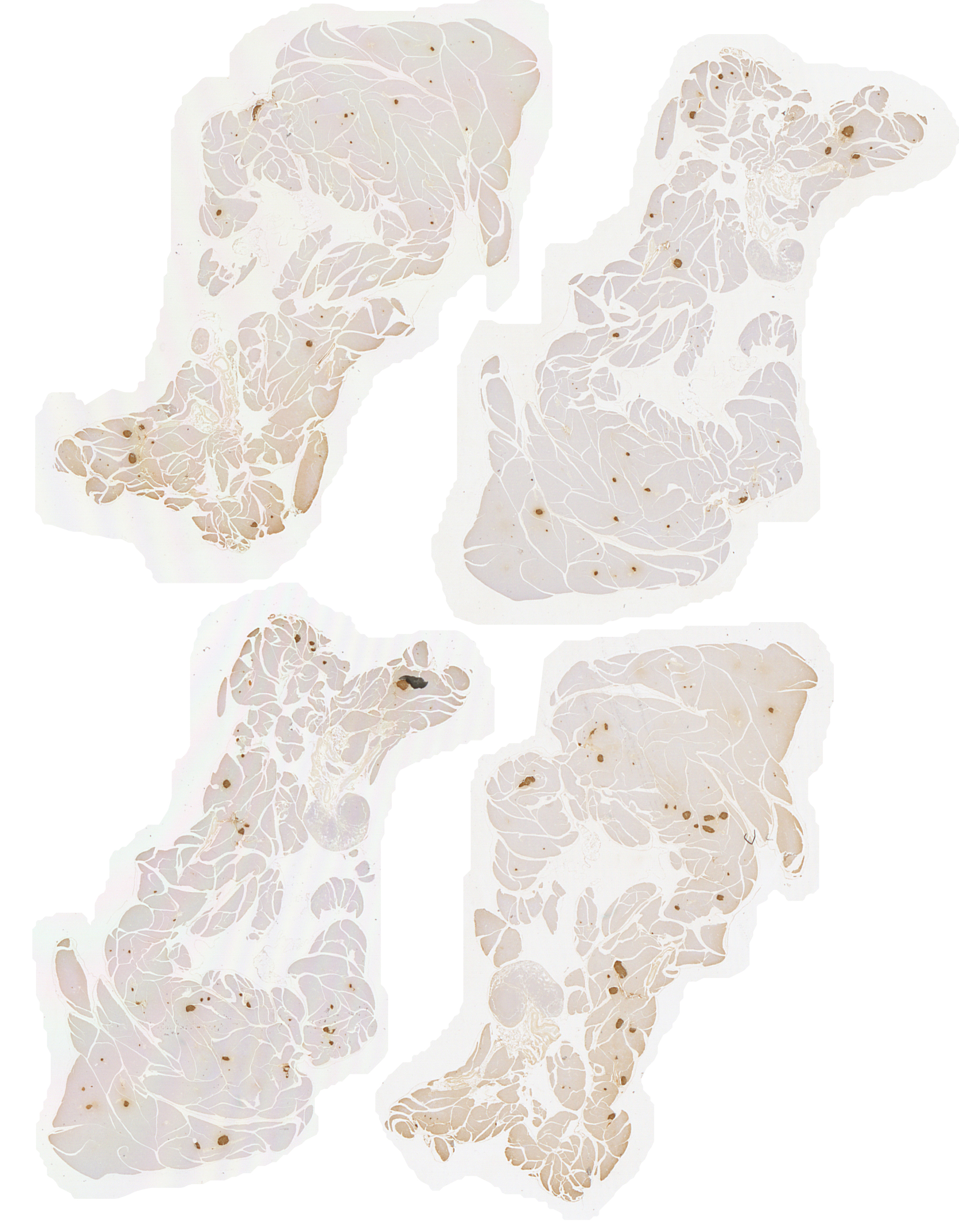

Supplement: Figure 1—source data 1. — The zip file contains all the 2400-dpi scanned images of insulin-immunostained pancreas sections used for quantitation of insulin-positive cell area. Insulin is stained as brown, and the whole section is counterstained as blue with hematoxylin. Folders are named after genotypes (P-Adn++ or P-Adn−−) and subfolders after time points (week 0, 2, 5, or 10) post initial dimerizer administration. Related to Figure 1A. DOI: http://dx.doi.org/10.7554/eLife.03851.004 [file elife03851s001.zip › Figure 1-source data 1/P-Adn++/Week 0/d47-2s d.jpg]

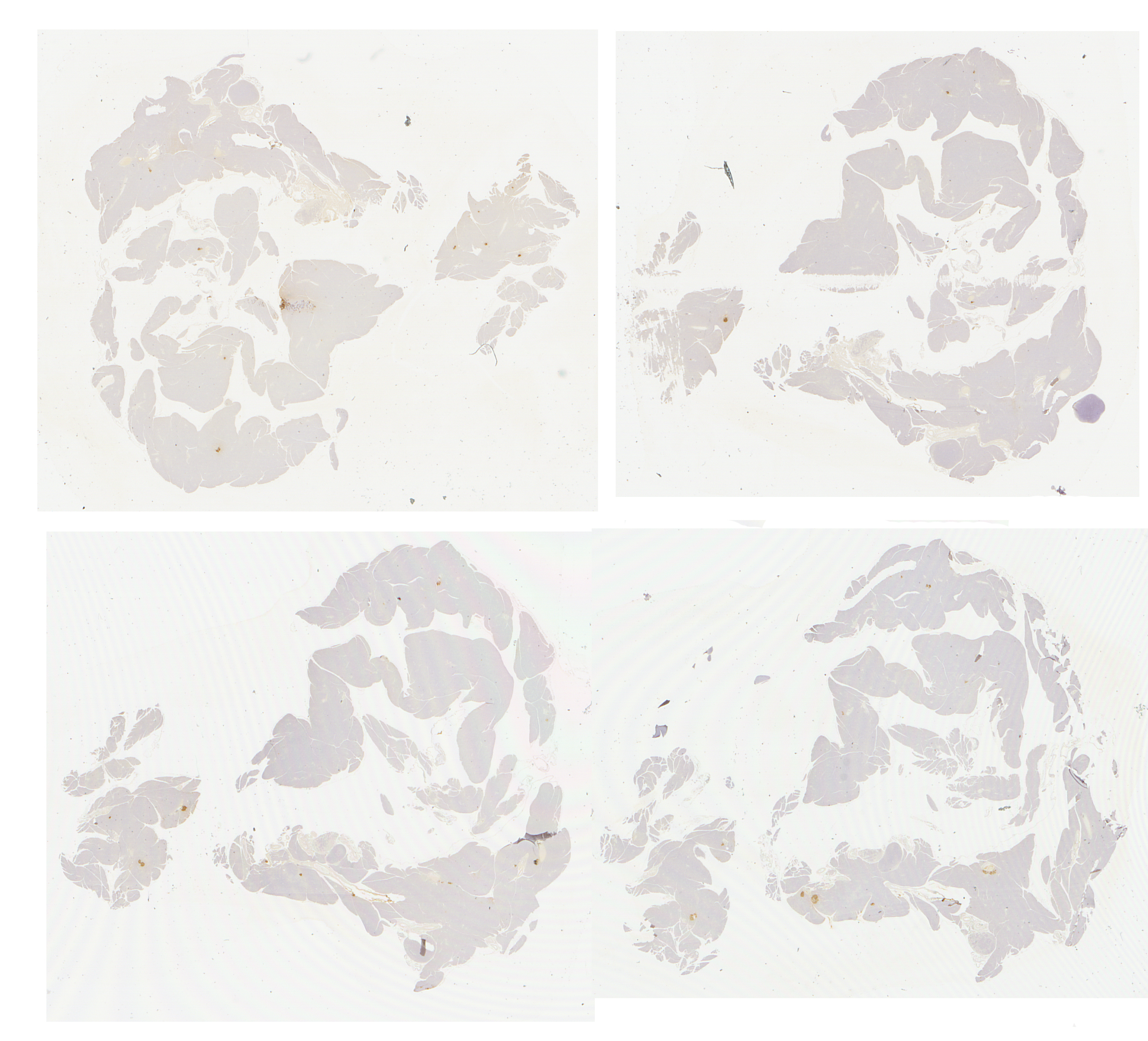

Supplement: Figure 1—source data 1. — The zip file contains all the 2400-dpi scanned images of insulin-immunostained pancreas sections used for quantitation of insulin-positive cell area. Insulin is stained as brown, and the whole section is counterstained as blue with hematoxylin. Folders are named after genotypes (P-Adn++ or P-Adn−−) and subfolders after time points (week 0, 2, 5, or 10) post initial dimerizer administration. Related to Figure 1A. DOI: http://dx.doi.org/10.7554/eLife.03851.004 [file elife03851s001.zip › Figure 1-source data 1/P-Adn++/Week 10/d22-5s a.jpg]

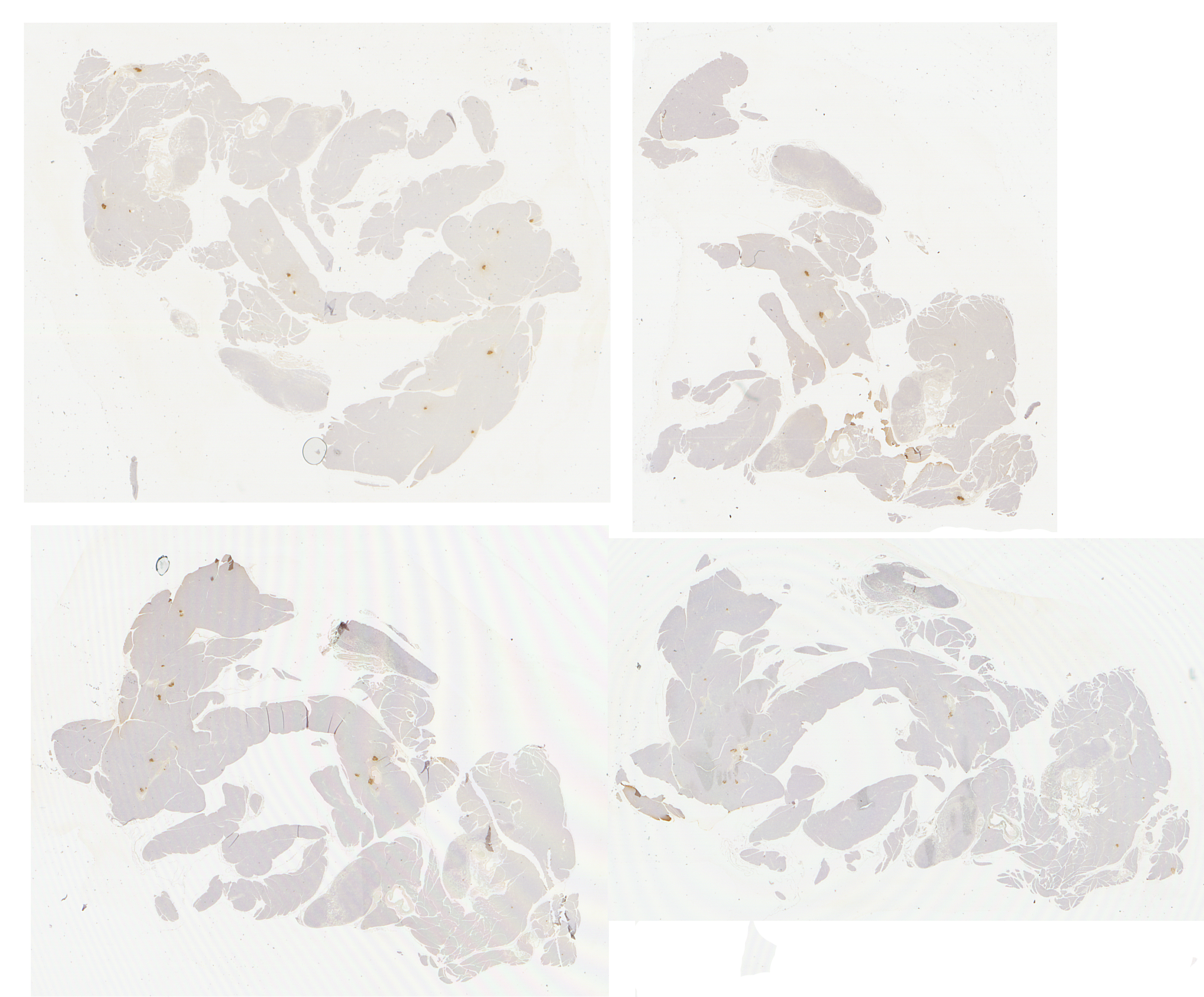

Supplement: Figure 1—source data 1. — The zip file contains all the 2400-dpi scanned images of insulin-immunostained pancreas sections used for quantitation of insulin-positive cell area. Insulin is stained as brown, and the whole section is counterstained as blue with hematoxylin. Folders are named after genotypes (P-Adn++ or P-Adn−−) and subfolders after time points (week 0, 2, 5, or 10) post initial dimerizer administration. Related to Figure 1A. DOI: http://dx.doi.org/10.7554/eLife.03851.004 [file elife03851s001.zip › Figure 1-source data 1/P-Adn++/Week 10/d22-5s b.jpg]

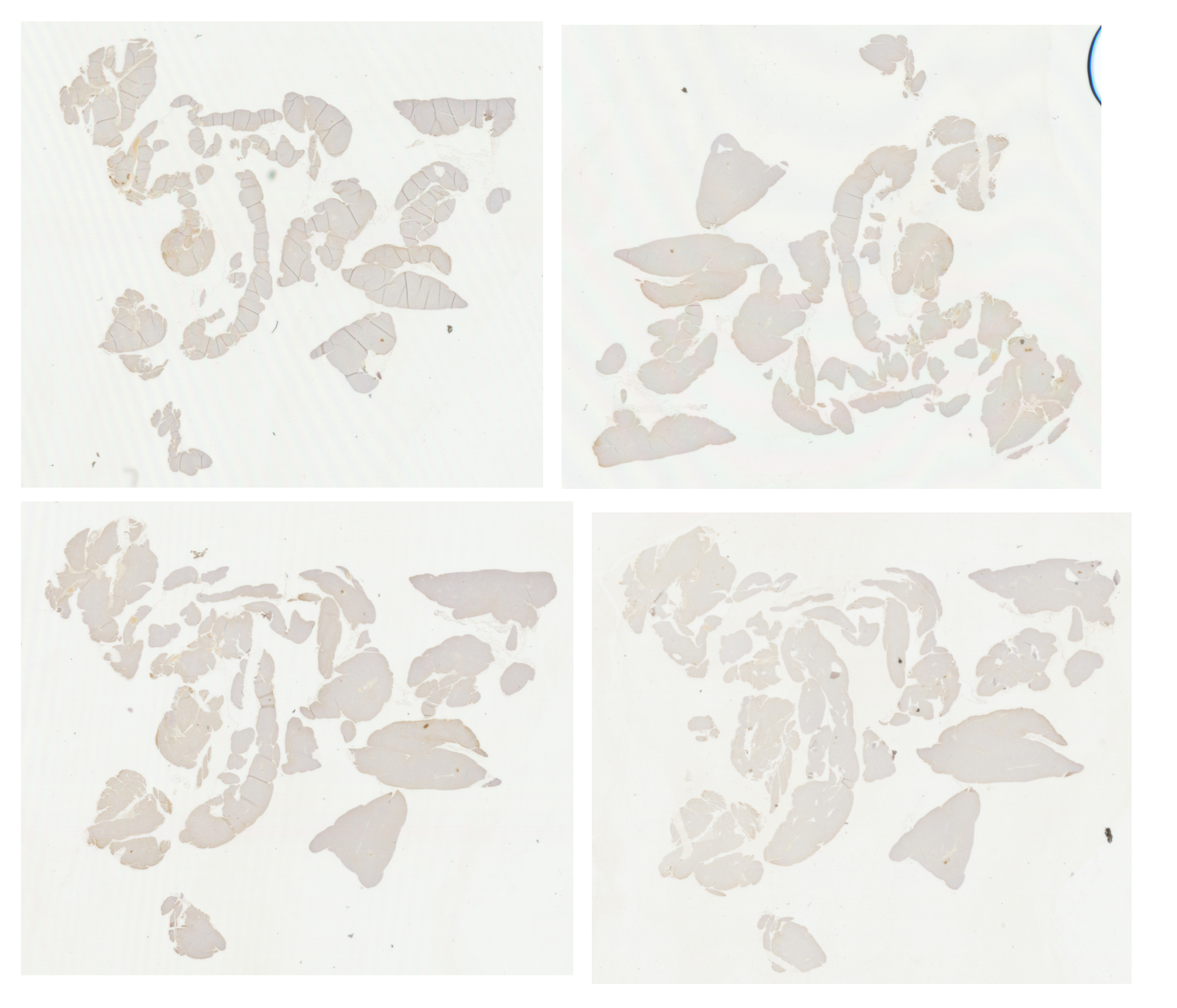

Supplement: Figure 1—source data 1. — The zip file contains all the 2400-dpi scanned images of insulin-immunostained pancreas sections used for quantitation of insulin-positive cell area. Insulin is stained as brown, and the whole section is counterstained as blue with hematoxylin. Folders are named after genotypes (P-Adn++ or P-Adn−−) and subfolders after time points (week 0, 2, 5, or 10) post initial dimerizer administration. Related to Figure 1A. DOI: http://dx.doi.org/10.7554/eLife.03851.004 [file elife03851s001.zip › Figure 1-source data 1/P-Adn++/Week 10/d39-3s a.jpg]

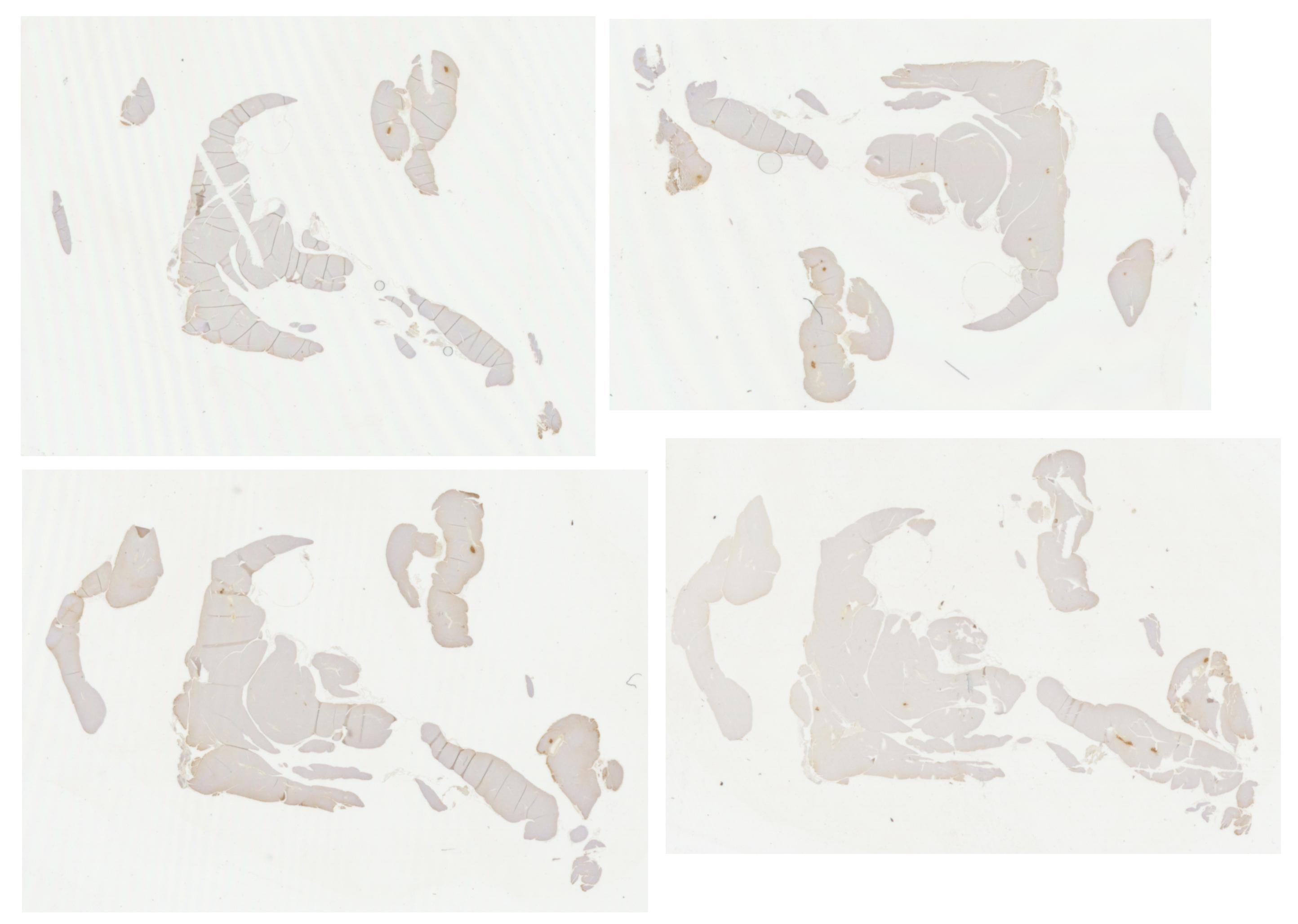

Supplement: Figure 1—source data 1. — The zip file contains all the 2400-dpi scanned images of insulin-immunostained pancreas sections used for quantitation of insulin-positive cell area. Insulin is stained as brown, and the whole section is counterstained as blue with hematoxylin. Folders are named after genotypes (P-Adn++ or P-Adn−−) and subfolders after time points (week 0, 2, 5, or 10) post initial dimerizer administration. Related to Figure 1A. DOI: http://dx.doi.org/10.7554/eLife.03851.004 [file elife03851s001.zip › Figure 1-source data 1/P-Adn++/Week 10/d39-3s b.jpg]

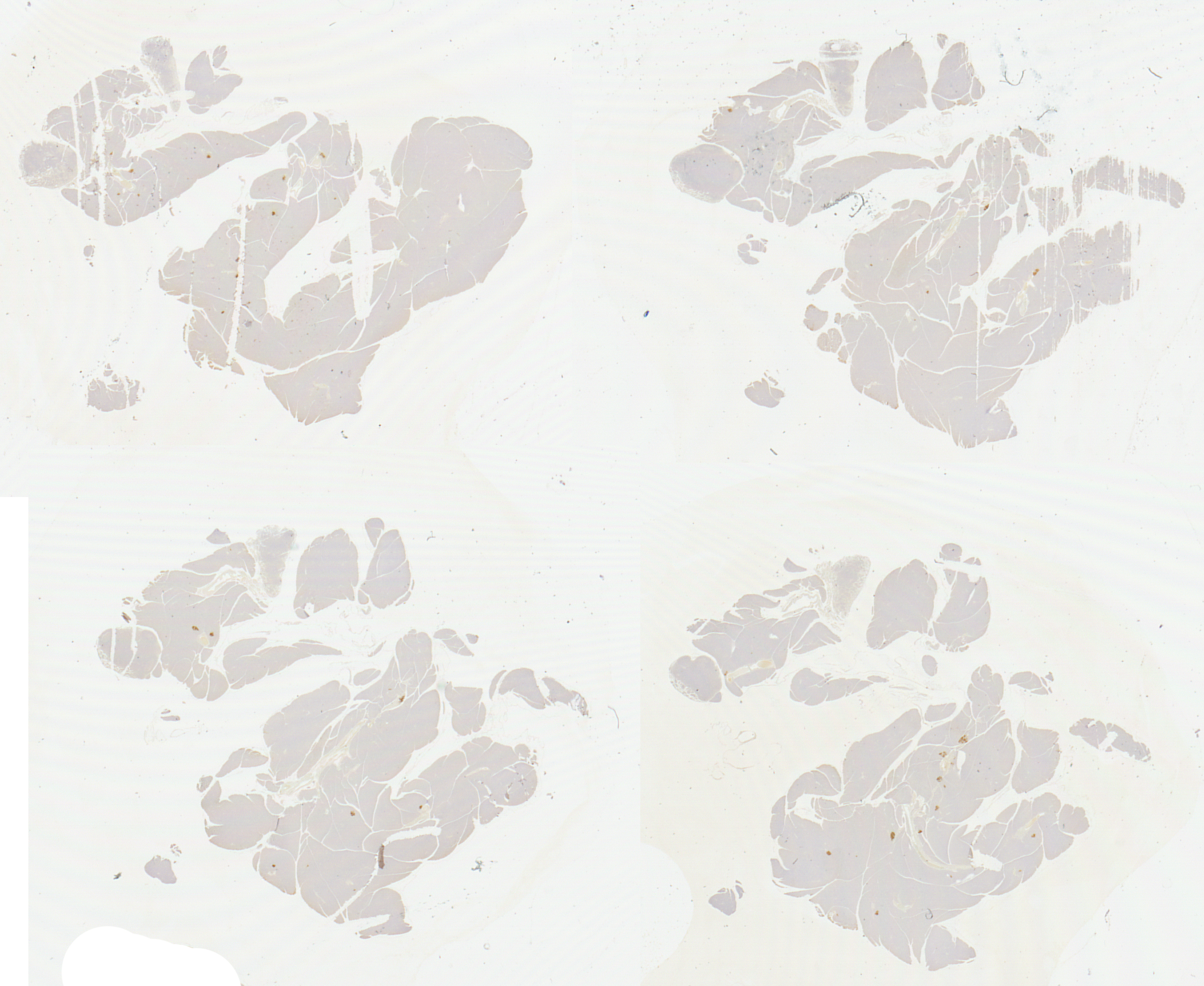

Supplement: Figure 1—source data 1. — The zip file contains all the 2400-dpi scanned images of insulin-immunostained pancreas sections used for quantitation of insulin-positive cell area. Insulin is stained as brown, and the whole section is counterstained as blue with hematoxylin. Folders are named after genotypes (P-Adn++ or P-Adn−−) and subfolders after time points (week 0, 2, 5, or 10) post initial dimerizer administration. Related to Figure 1A. DOI: http://dx.doi.org/10.7554/eLife.03851.004 [file elife03851s001.zip › Figure 1-source data 1/P-Adn++/Week 2/d45-12 a.jpg]

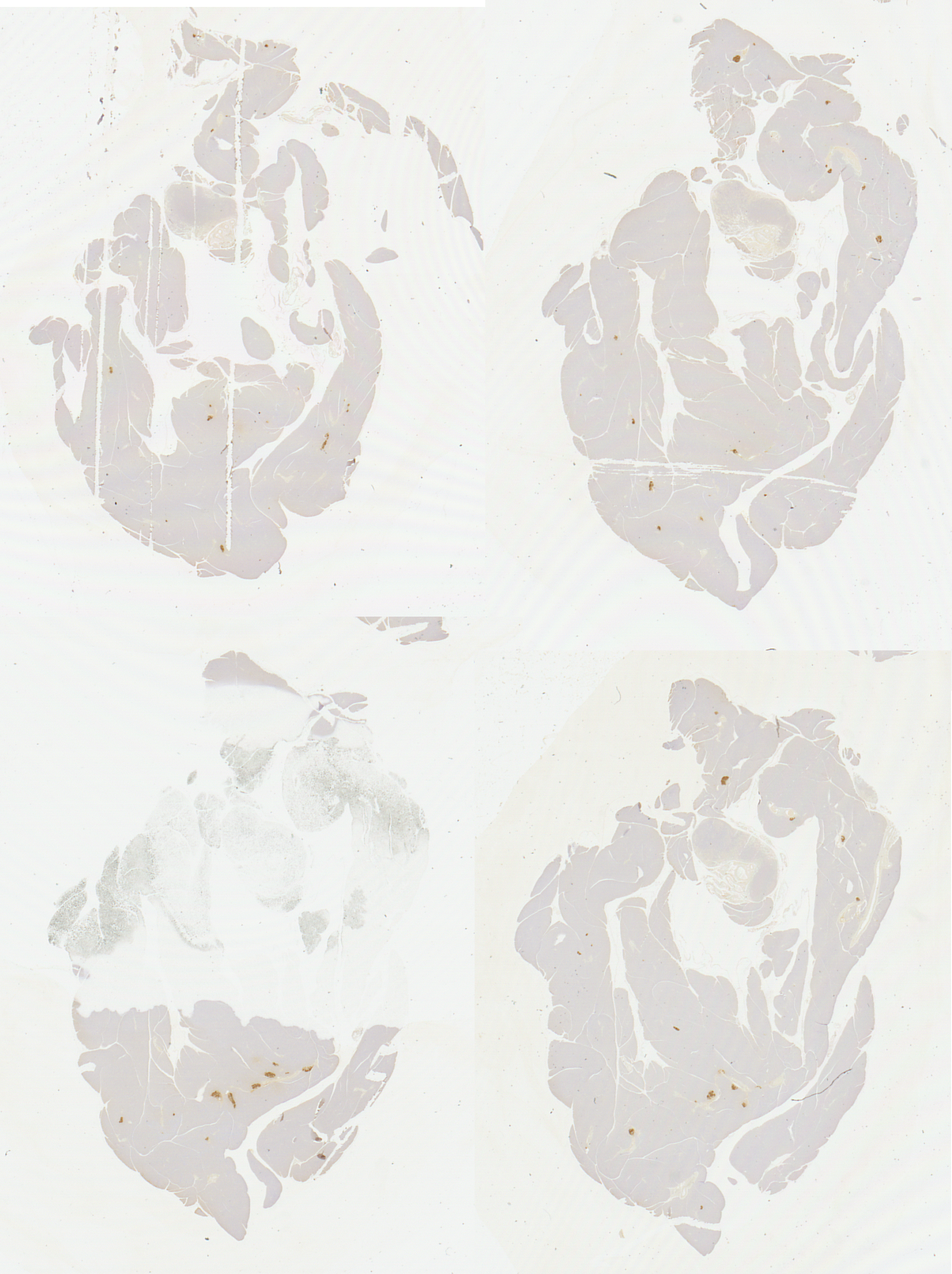

Supplement: Figure 1—source data 1. — The zip file contains all the 2400-dpi scanned images of insulin-immunostained pancreas sections used for quantitation of insulin-positive cell area. Insulin is stained as brown, and the whole section is counterstained as blue with hematoxylin. Folders are named after genotypes (P-Adn++ or P-Adn−−) and subfolders after time points (week 0, 2, 5, or 10) post initial dimerizer administration. Related to Figure 1A. DOI: http://dx.doi.org/10.7554/eLife.03851.004 [file elife03851s001.zip › Figure 1-source data 1/P-Adn++/Week 2/d45-12 b.jpg]

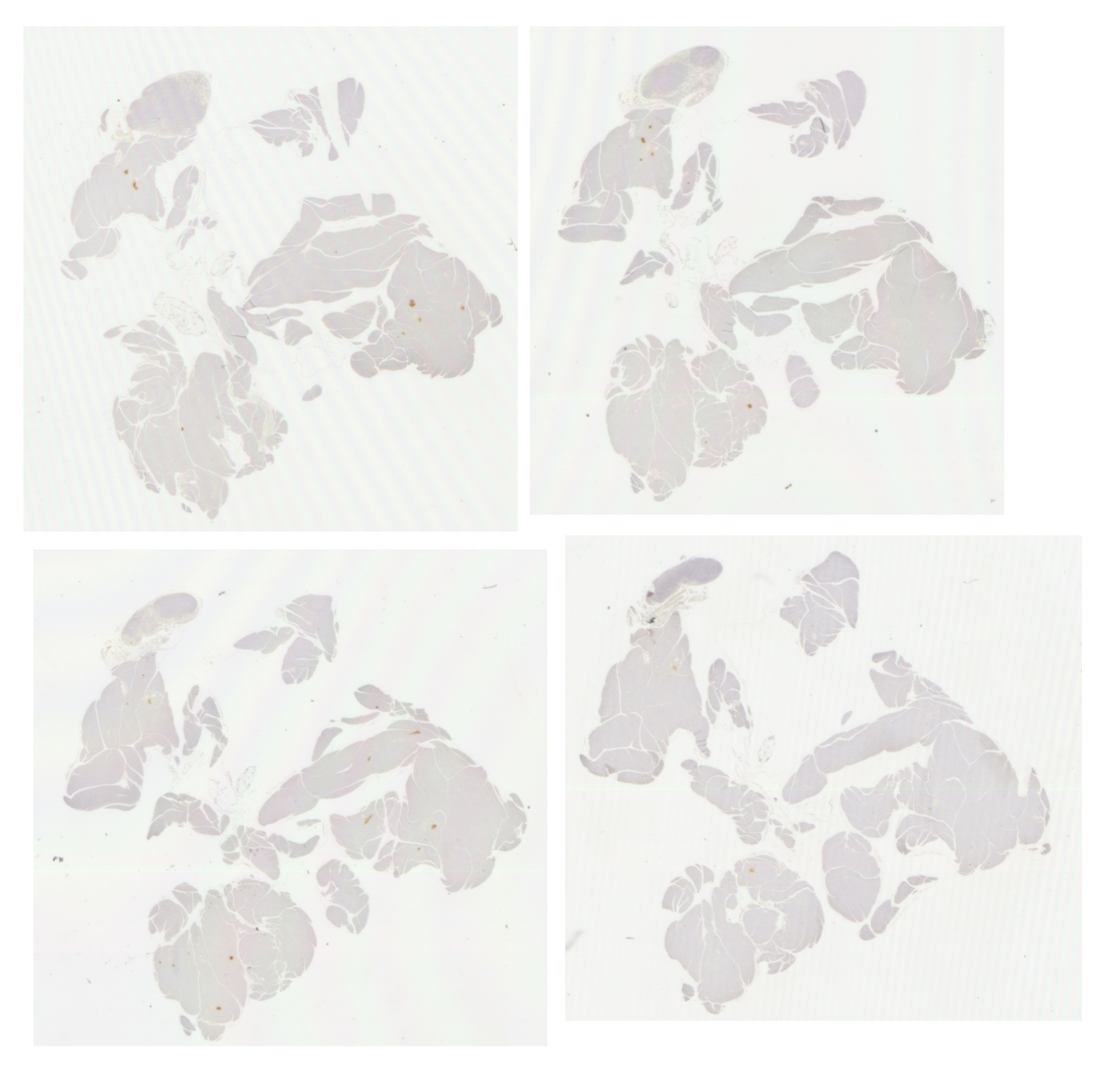

Supplement: Figure 1—source data 1. — The zip file contains all the 2400-dpi scanned images of insulin-immunostained pancreas sections used for quantitation of insulin-positive cell area. Insulin is stained as brown, and the whole section is counterstained as blue with hematoxylin. Folders are named after genotypes (P-Adn++ or P-Adn−−) and subfolders after time points (week 0, 2, 5, or 10) post initial dimerizer administration. Related to Figure 1A. DOI: http://dx.doi.org/10.7554/eLife.03851.004 [file elife03851s001.zip › Figure 1-source data 1/P-Adn++/Week 2/d46-14 a.jpg]

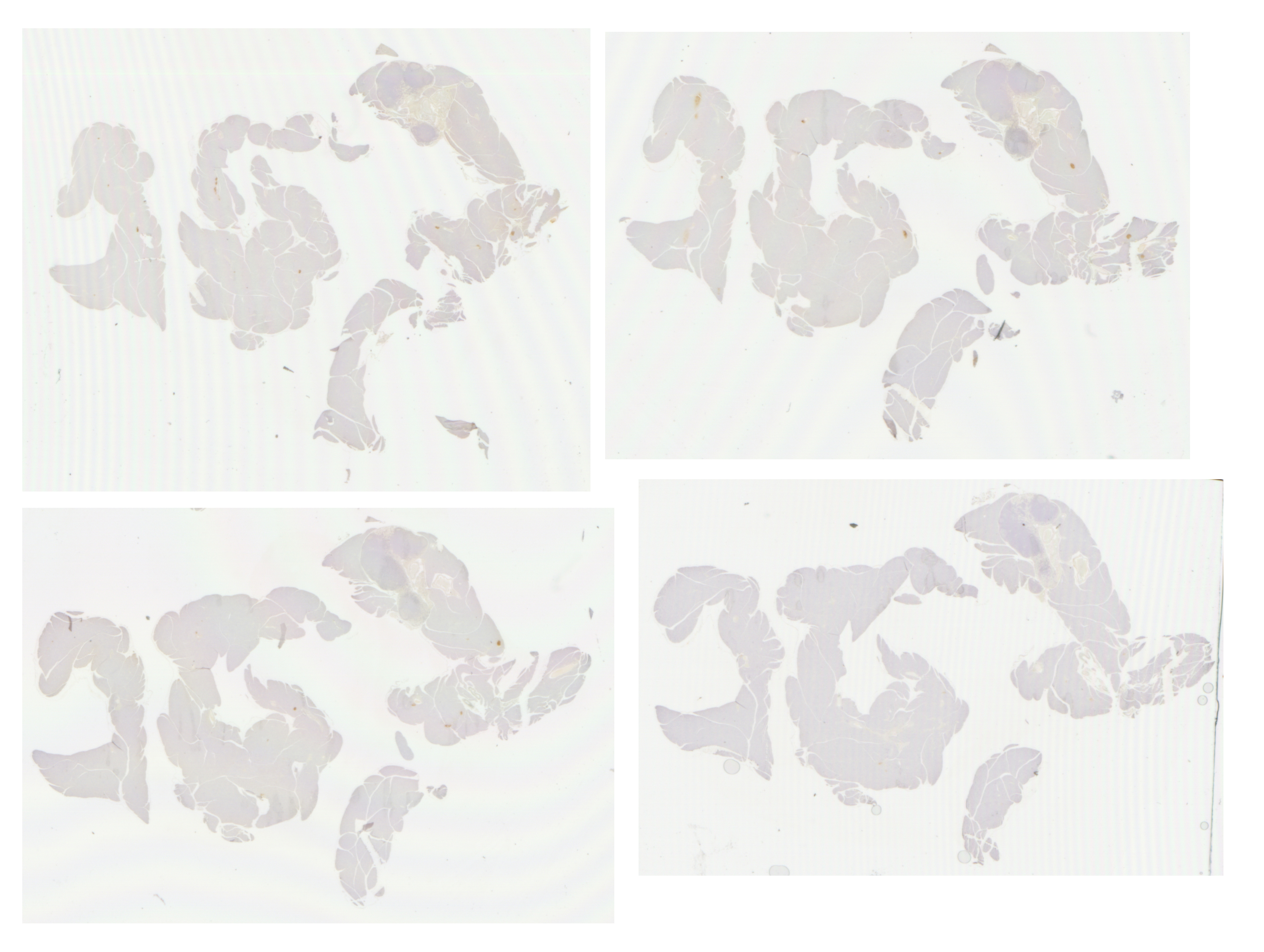

Supplement: Figure 1—source data 1. — The zip file contains all the 2400-dpi scanned images of insulin-immunostained pancreas sections used for quantitation of insulin-positive cell area. Insulin is stained as brown, and the whole section is counterstained as blue with hematoxylin. Folders are named after genotypes (P-Adn++ or P-Adn−−) and subfolders after time points (week 0, 2, 5, or 10) post initial dimerizer administration. Related to Figure 1A. DOI: http://dx.doi.org/10.7554/eLife.03851.004 [file elife03851s001.zip › Figure 1-source data 1/P-Adn++/Week 2/d46-14 b.jpg]

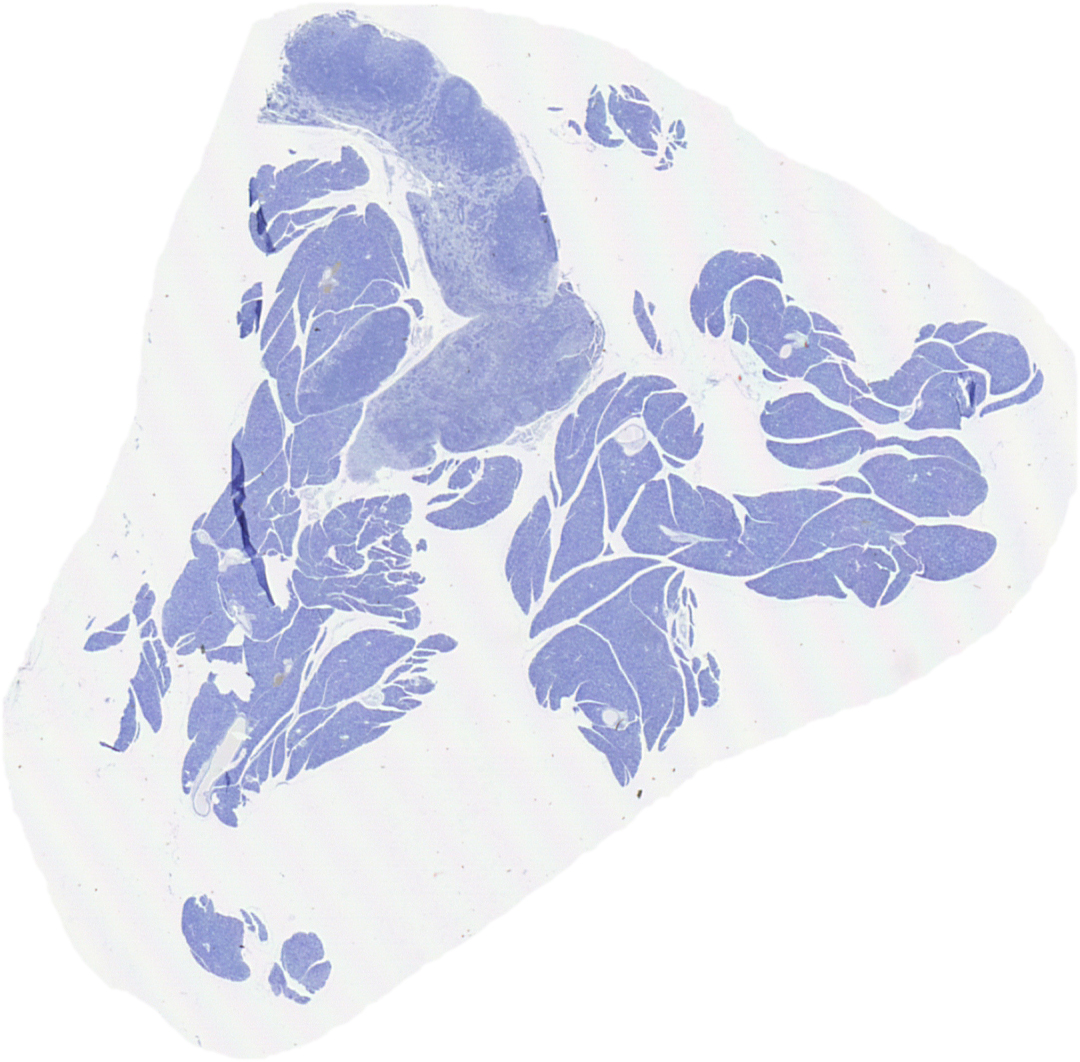

Supplement: Figure 1—source data 1. — The zip file contains all the 2400-dpi scanned images of insulin-immunostained pancreas sections used for quantitation of insulin-positive cell area. Insulin is stained as brown, and the whole section is counterstained as blue with hematoxylin. Folders are named after genotypes (P-Adn++ or P-Adn−−) and subfolders after time points (week 0, 2, 5, or 10) post initial dimerizer administration. Related to Figure 1A. DOI: http://dx.doi.org/10.7554/eLife.03851.004 [file elife03851s001.zip › Figure 1-source data 1/P-Adn++/Week 5/d55-2s-D2 a.jpg]

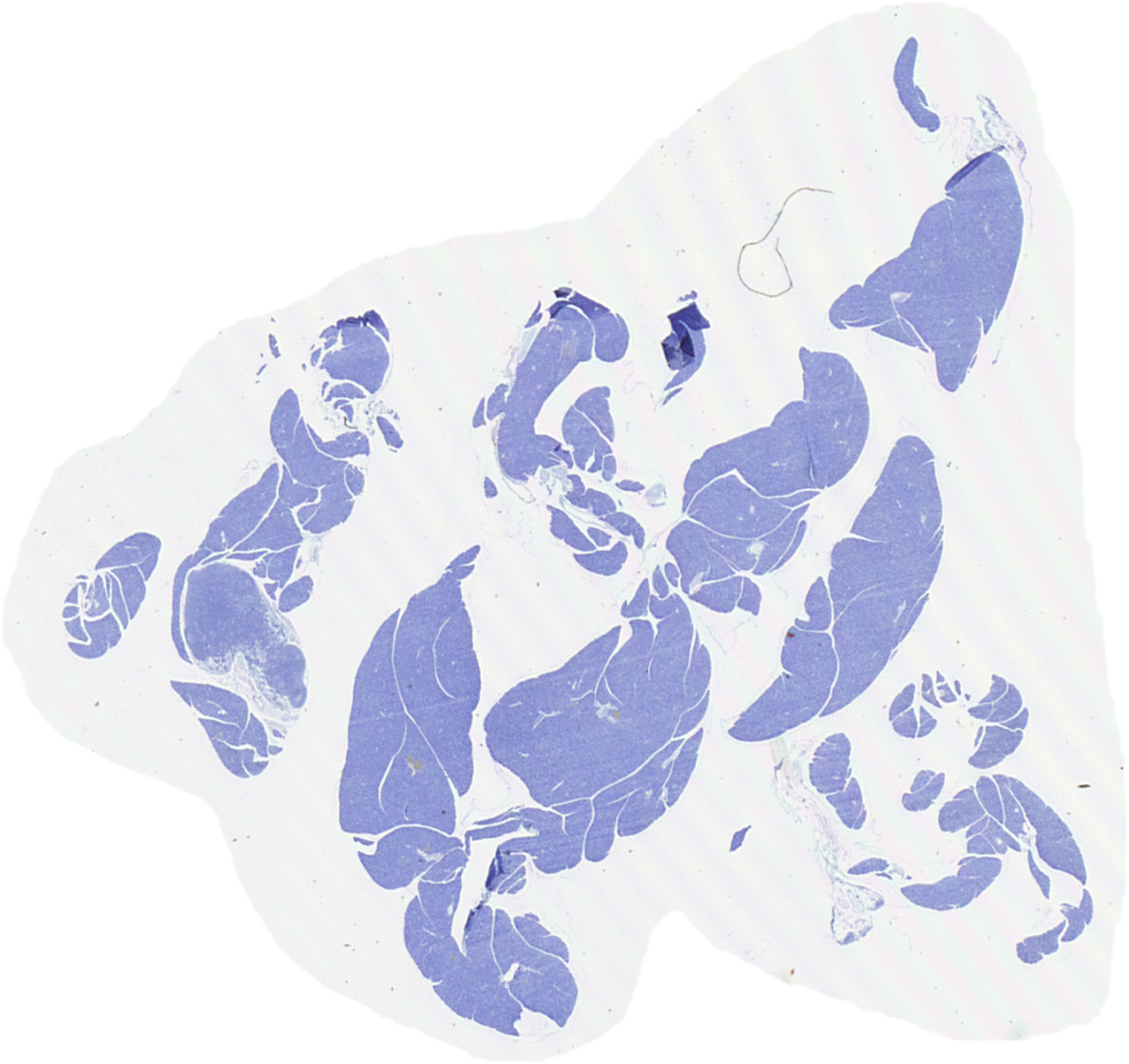

Supplement: Figure 1—source data 1. — The zip file contains all the 2400-dpi scanned images of insulin-immunostained pancreas sections used for quantitation of insulin-positive cell area. Insulin is stained as brown, and the whole section is counterstained as blue with hematoxylin. Folders are named after genotypes (P-Adn++ or P-Adn−−) and subfolders after time points (week 0, 2, 5, or 10) post initial dimerizer administration. Related to Figure 1A. DOI: http://dx.doi.org/10.7554/eLife.03851.004 [file elife03851s001.zip › Figure 1-source data 1/P-Adn++/Week 5/d55-2s-D2 b.jpg]

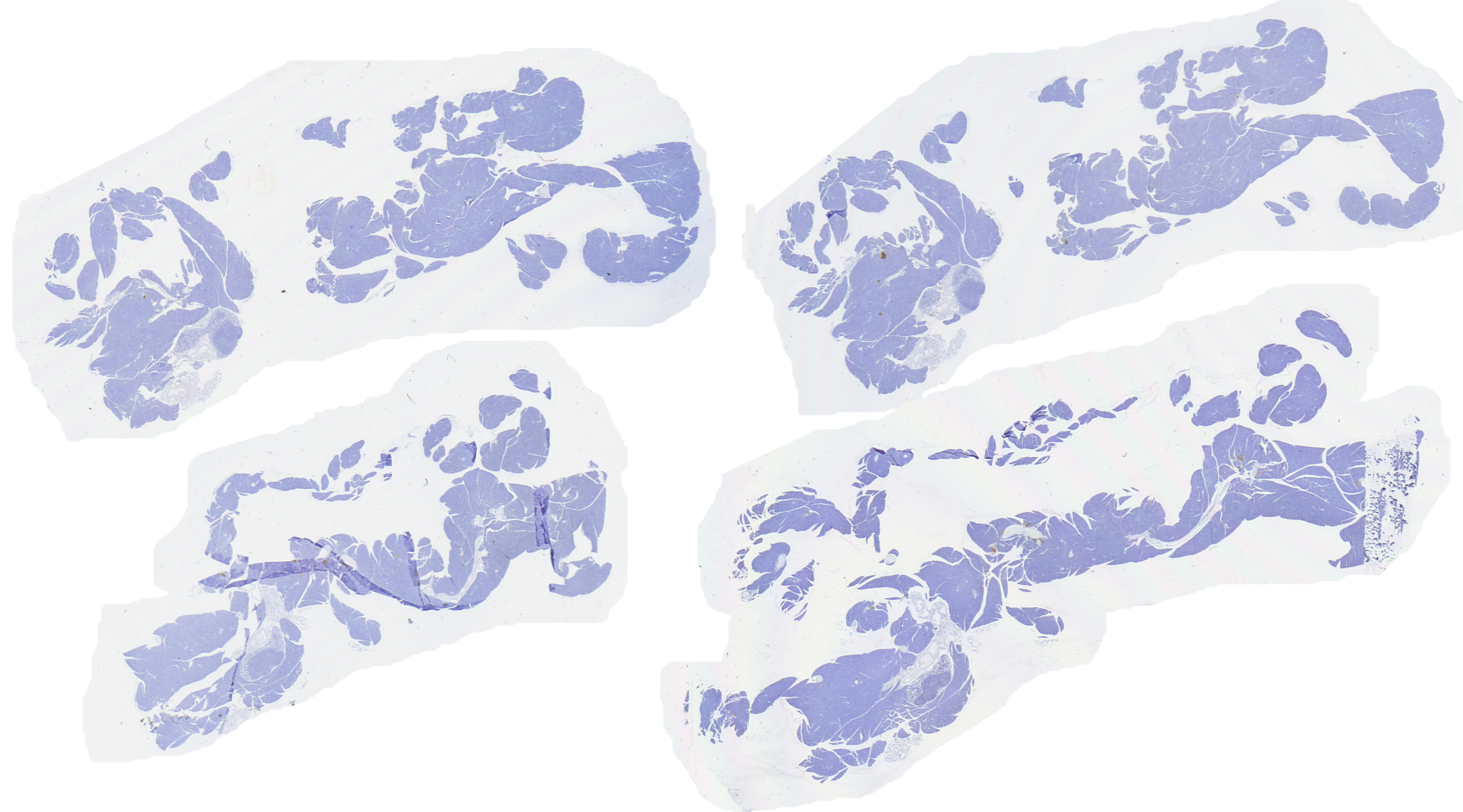

Supplement: Figure 1—source data 1. — The zip file contains all the 2400-dpi scanned images of insulin-immunostained pancreas sections used for quantitation of insulin-positive cell area. Insulin is stained as brown, and the whole section is counterstained as blue with hematoxylin. Folders are named after genotypes (P-Adn++ or P-Adn−−) and subfolders after time points (week 0, 2, 5, or 10) post initial dimerizer administration. Related to Figure 1A. DOI: http://dx.doi.org/10.7554/eLife.03851.004 [file elife03851s001.zip › Figure 1-source data 1/P-Adn++/Week 5/d67-1s a.jpg]

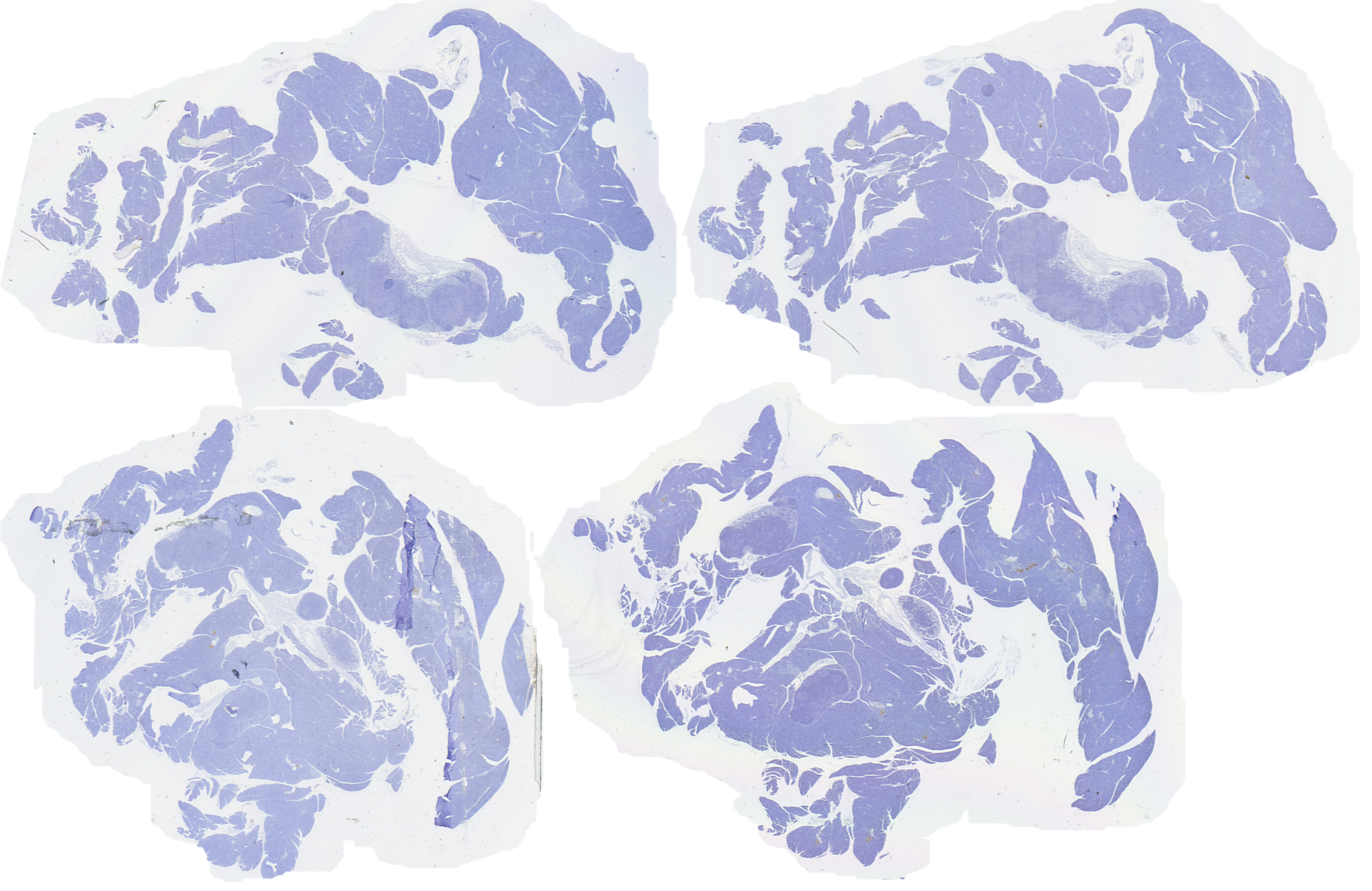

Supplement: Figure 1—source data 1. — The zip file contains all the 2400-dpi scanned images of insulin-immunostained pancreas sections used for quantitation of insulin-positive cell area. Insulin is stained as brown, and the whole section is counterstained as blue with hematoxylin. Folders are named after genotypes (P-Adn++ or P-Adn−−) and subfolders after time points (week 0, 2, 5, or 10) post initial dimerizer administration. Related to Figure 1A. DOI: http://dx.doi.org/10.7554/eLife.03851.004 [file elife03851s001.zip › Figure 1-source data 1/P-Adn++/Week 5/d67-1s b.jpg]

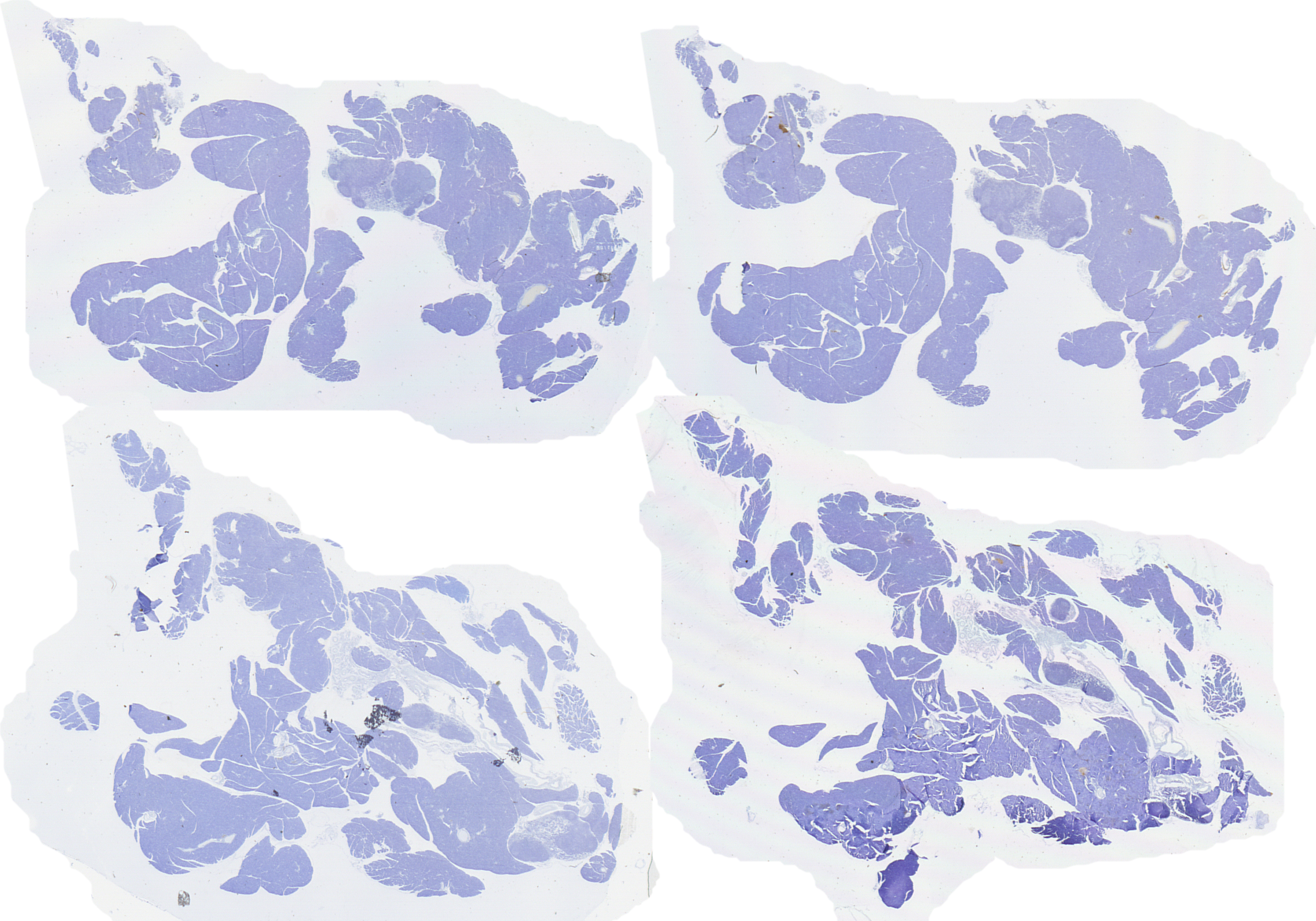

Supplement: Figure 1—source data 1. — The zip file contains all the 2400-dpi scanned images of insulin-immunostained pancreas sections used for quantitation of insulin-positive cell area. Insulin is stained as brown, and the whole section is counterstained as blue with hematoxylin. Folders are named after genotypes (P-Adn++ or P-Adn−−) and subfolders after time points (week 0, 2, 5, or 10) post initial dimerizer administration. Related to Figure 1A. DOI: http://dx.doi.org/10.7554/eLife.03851.004 [file elife03851s001.zip › Figure 1-source data 1/P-Adn++/Week 5/d67-1s c.jpg]

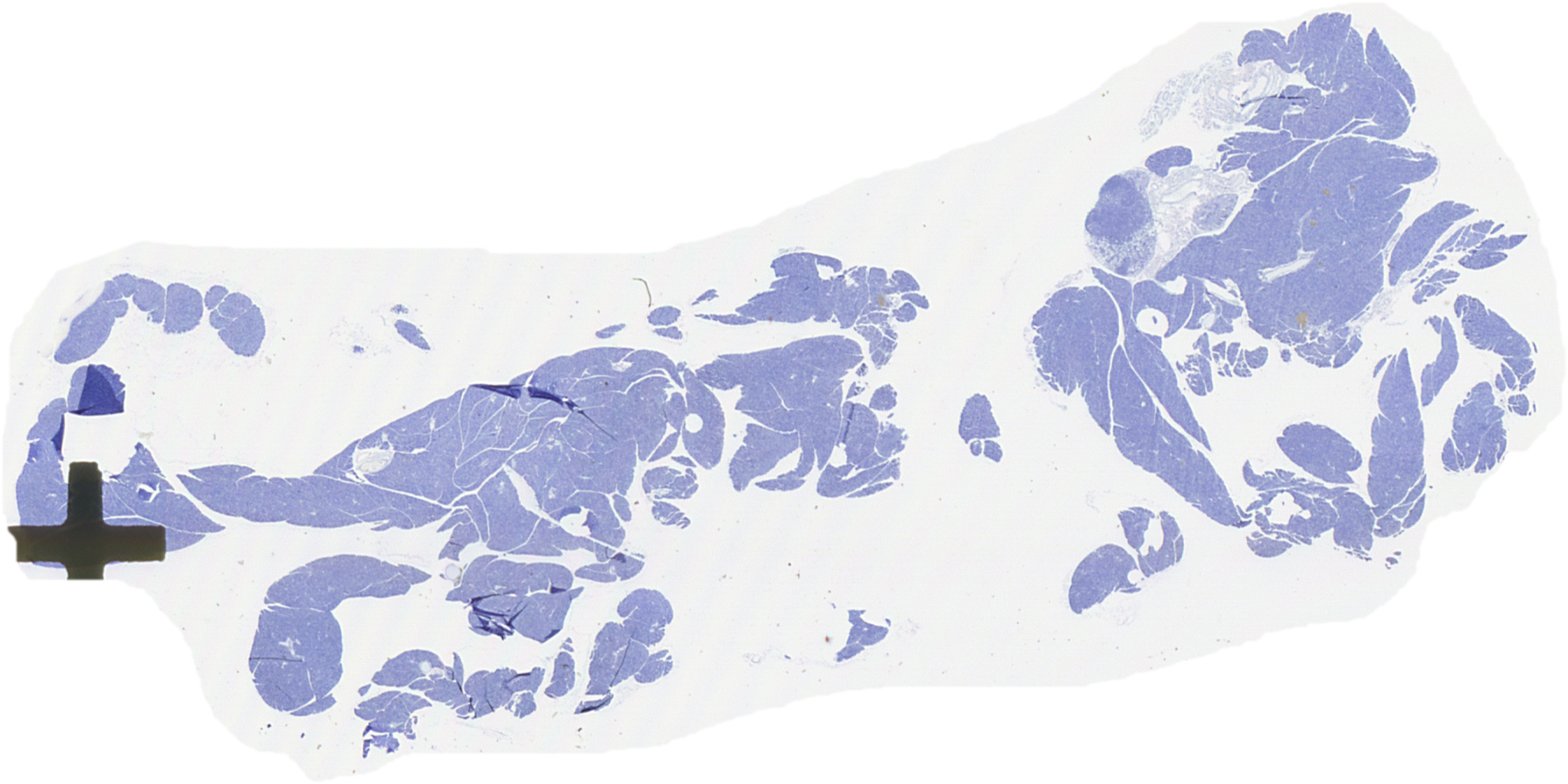

Supplement: Figure 1—source data 1. — The zip file contains all the 2400-dpi scanned images of insulin-immunostained pancreas sections used for quantitation of insulin-positive cell area. Insulin is stained as brown, and the whole section is counterstained as blue with hematoxylin. Folders are named after genotypes (P-Adn++ or P-Adn−−) and subfolders after time points (week 0, 2, 5, or 10) post initial dimerizer administration. Related to Figure 1A. DOI: http://dx.doi.org/10.7554/eLife.03851.004 [file elife03851s001.zip › Figure 1-source data 1/P-Adn++/Week 5/d67-1s-B6 a.jpg]

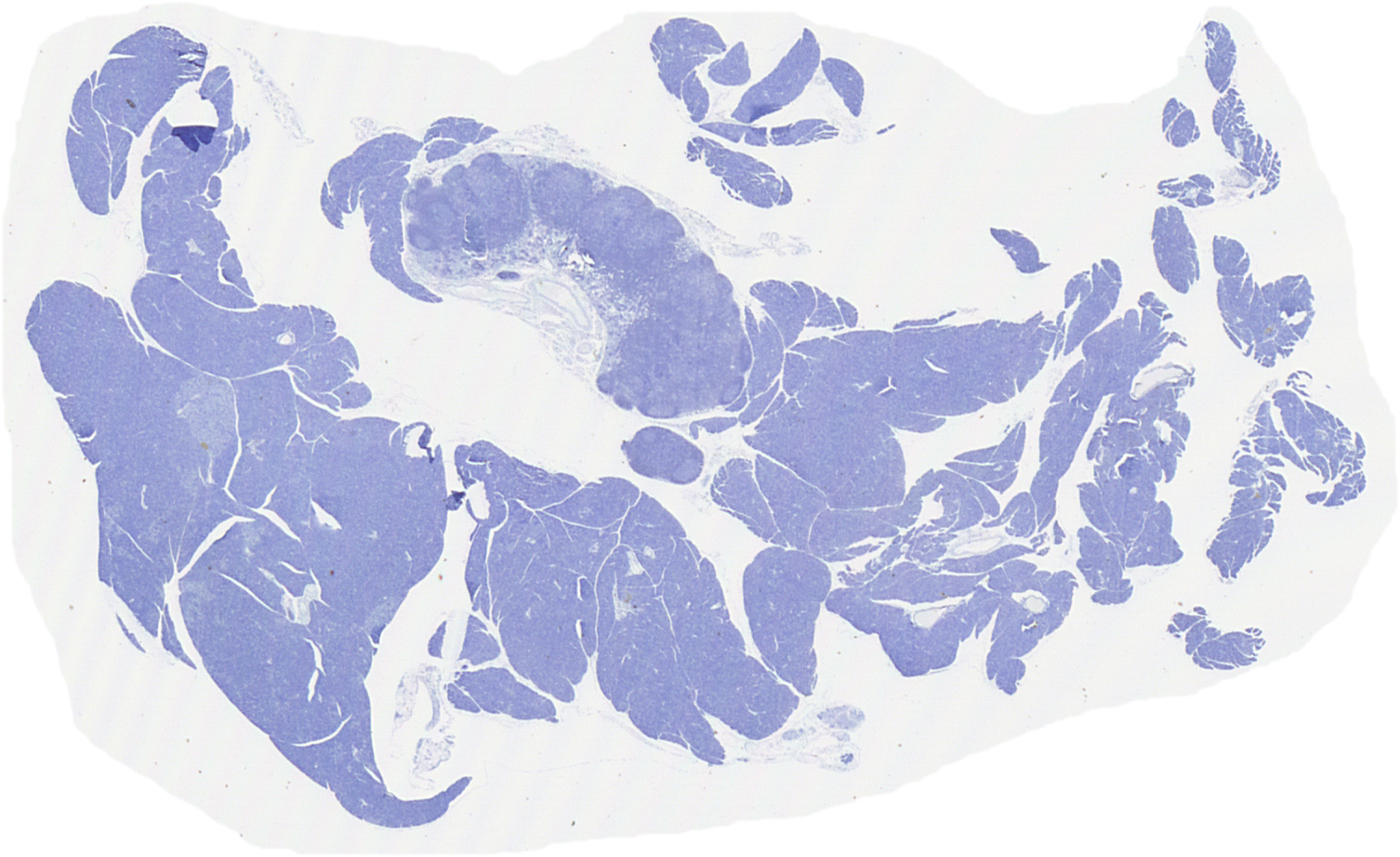

Supplement: Figure 1—source data 1. — The zip file contains all the 2400-dpi scanned images of insulin-immunostained pancreas sections used for quantitation of insulin-positive cell area. Insulin is stained as brown, and the whole section is counterstained as blue with hematoxylin. Folders are named after genotypes (P-Adn++ or P-Adn−−) and subfolders after time points (week 0, 2, 5, or 10) post initial dimerizer administration. Related to Figure 1A. DOI: http://dx.doi.org/10.7554/eLife.03851.004 [file elife03851s001.zip › Figure 1-source data 1/P-Adn++/Week 5/d67-1s-B6 b.jpg]

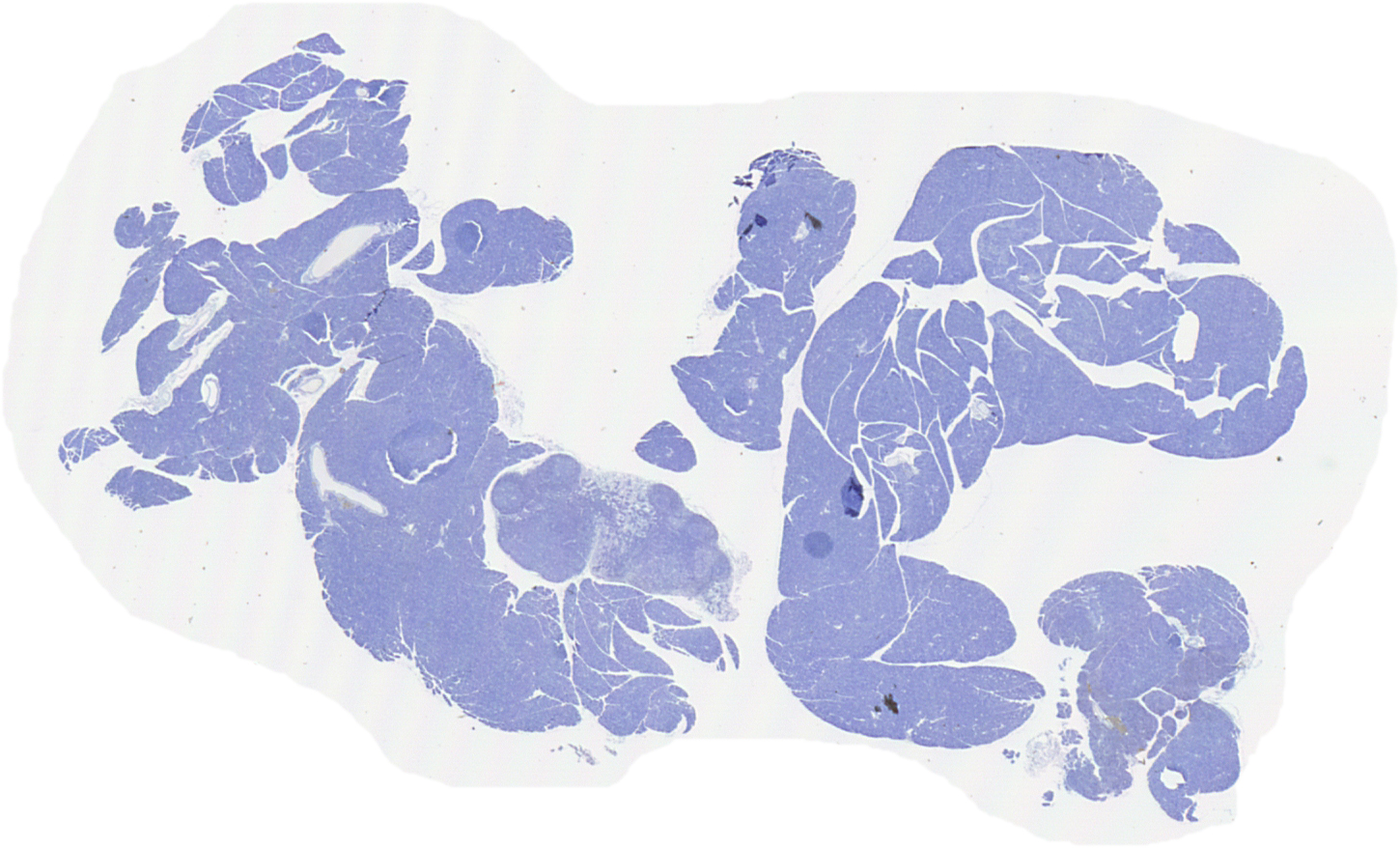

Supplement: Figure 1—source data 1. — The zip file contains all the 2400-dpi scanned images of insulin-immunostained pancreas sections used for quantitation of insulin-positive cell area. Insulin is stained as brown, and the whole section is counterstained as blue with hematoxylin. Folders are named after genotypes (P-Adn++ or P-Adn−−) and subfolders after time points (week 0, 2, 5, or 10) post initial dimerizer administration. Related to Figure 1A. DOI: http://dx.doi.org/10.7554/eLife.03851.004 [file elife03851s001.zip › Figure 1-source data 1/P-Adn++/Week 5/d67-1s-B6 c.jpg]

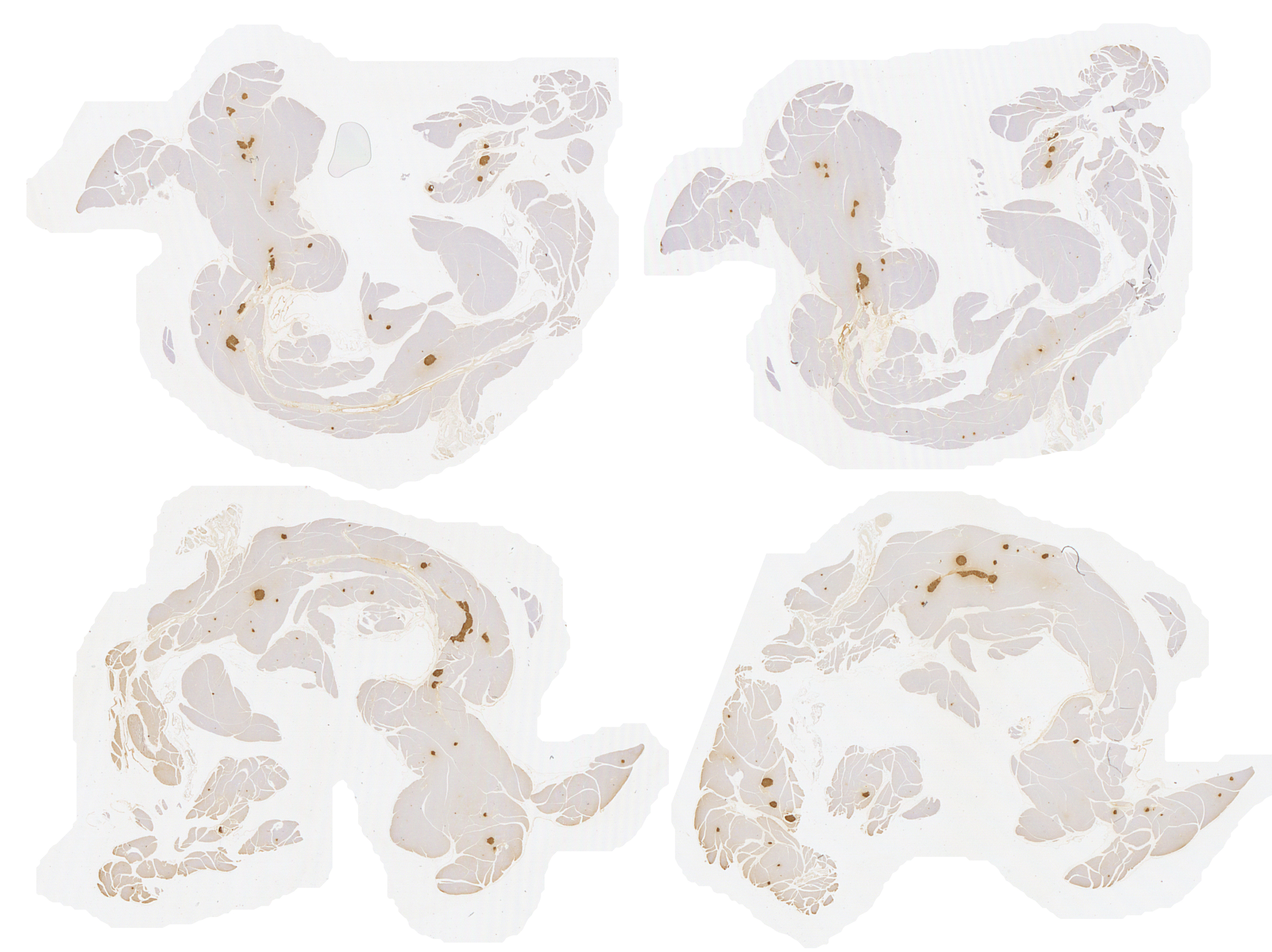

Supplement: Figure 7—source data 1. — The zip file contains all the 2400-dpi scanned images of insulin-immunostained pancreas sections used for quantitation of insulin-positive cell area. Insulin is stained as brown, and the whole section is counterstained as blue with hematoxylin. Folders are named after genotypes (WT or P-AdnTg+) and subfolders after time points (week 0, 2, 5, or 10) post initial dimerizer administration. Related to Figure 7A. DOI: http://dx.doi.org/10.7554/eLife.03851.031 [file elife03851s002.zip › Figure 7-source data 1/P-AdnTg+/Week 0/d47-1s a.jpg]

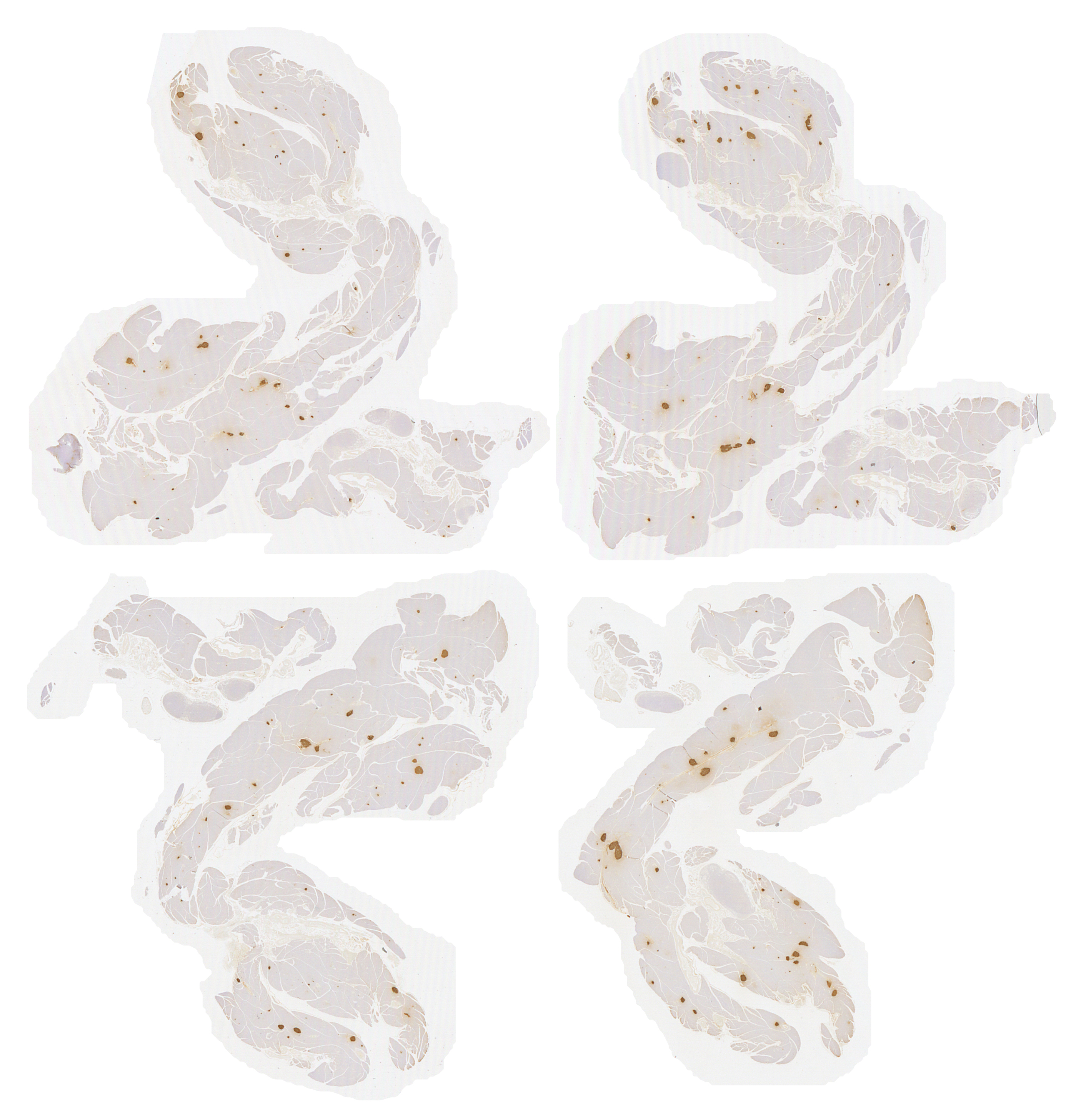

Supplement: Figure 7—source data 1. — The zip file contains all the 2400-dpi scanned images of insulin-immunostained pancreas sections used for quantitation of insulin-positive cell area. Insulin is stained as brown, and the whole section is counterstained as blue with hematoxylin. Folders are named after genotypes (WT or P-AdnTg+) and subfolders after time points (week 0, 2, 5, or 10) post initial dimerizer administration. Related to Figure 7A. DOI: http://dx.doi.org/10.7554/eLife.03851.031 [file elife03851s002.zip › Figure 7-source data 1/P-AdnTg+/Week 0/d47-1s b.jpg]

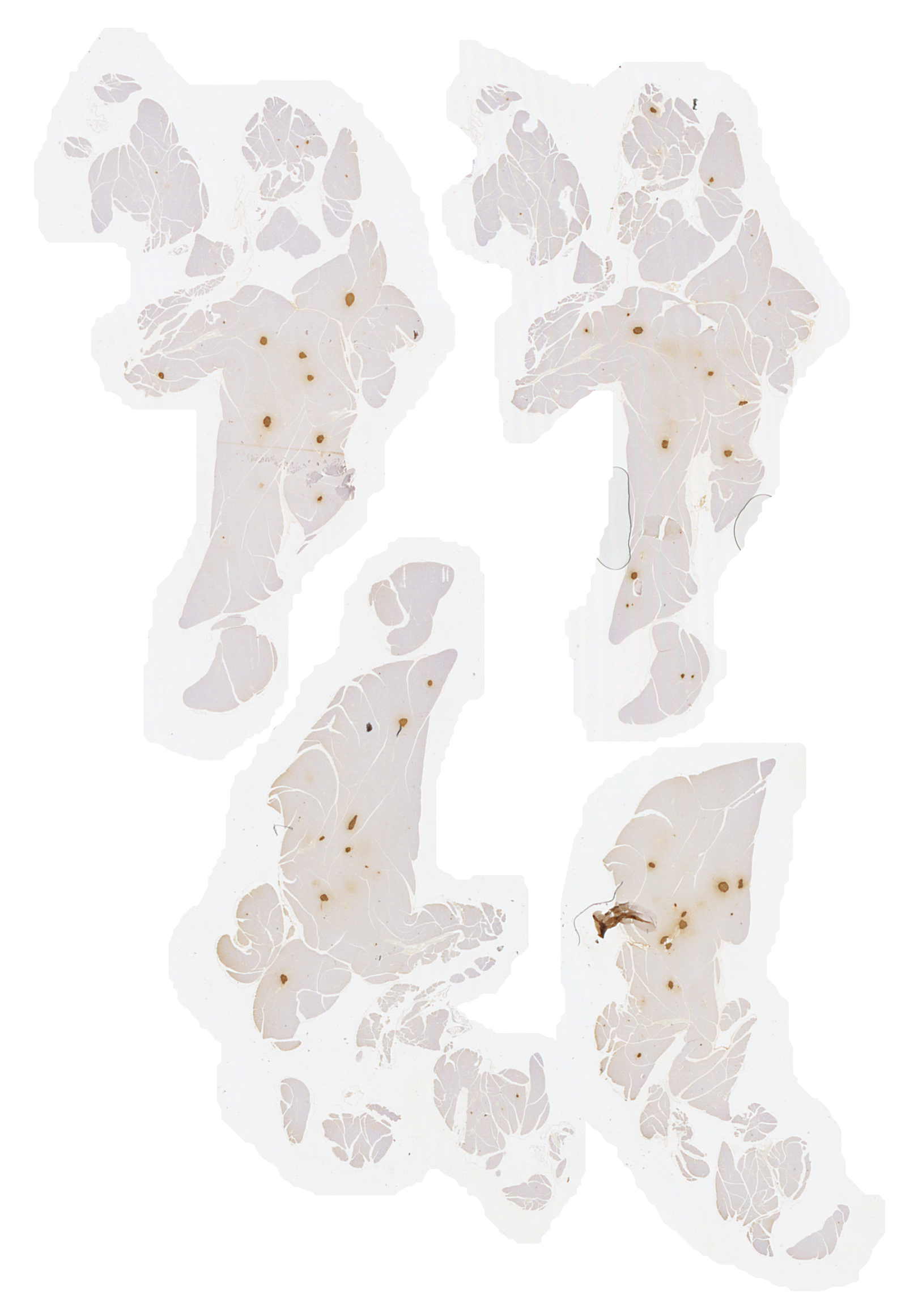

Supplement: Figure 7—source data 1. — The zip file contains all the 2400-dpi scanned images of insulin-immunostained pancreas sections used for quantitation of insulin-positive cell area. Insulin is stained as brown, and the whole section is counterstained as blue with hematoxylin. Folders are named after genotypes (WT or P-AdnTg+) and subfolders after time points (week 0, 2, 5, or 10) post initial dimerizer administration. Related to Figure 7A. DOI: http://dx.doi.org/10.7554/eLife.03851.031 [file elife03851s002.zip › Figure 7-source data 1/P-AdnTg+/Week 0/d47-1s c.jpg]

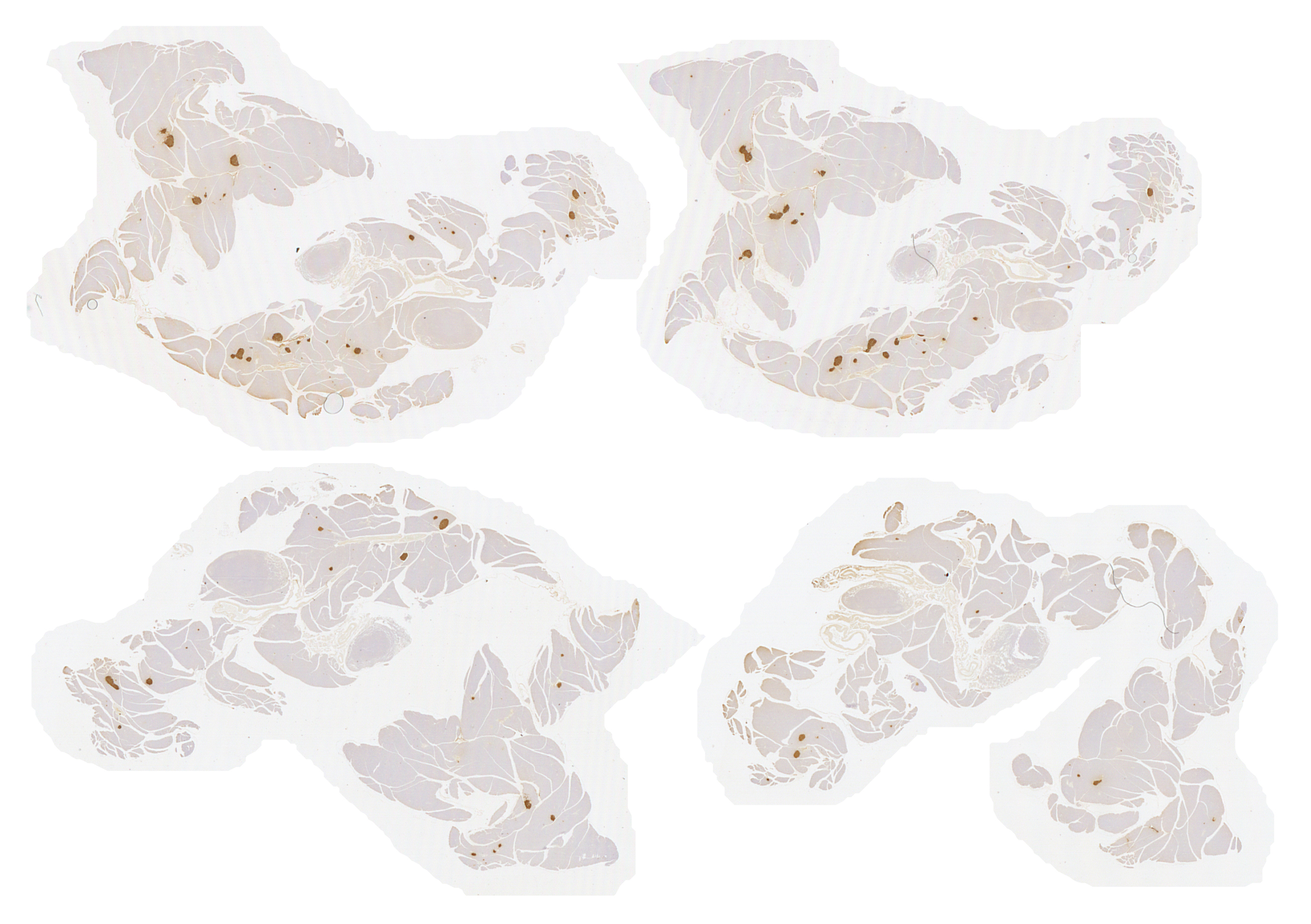

Supplement: Figure 7—source data 1. — The zip file contains all the 2400-dpi scanned images of insulin-immunostained pancreas sections used for quantitation of insulin-positive cell area. Insulin is stained as brown, and the whole section is counterstained as blue with hematoxylin. Folders are named after genotypes (WT or P-AdnTg+) and subfolders after time points (week 0, 2, 5, or 10) post initial dimerizer administration. Related to Figure 7A. DOI: http://dx.doi.org/10.7554/eLife.03851.031 [file elife03851s002.zip › Figure 7-source data 1/P-AdnTg+/Week 0/d47-1s d.jpg]

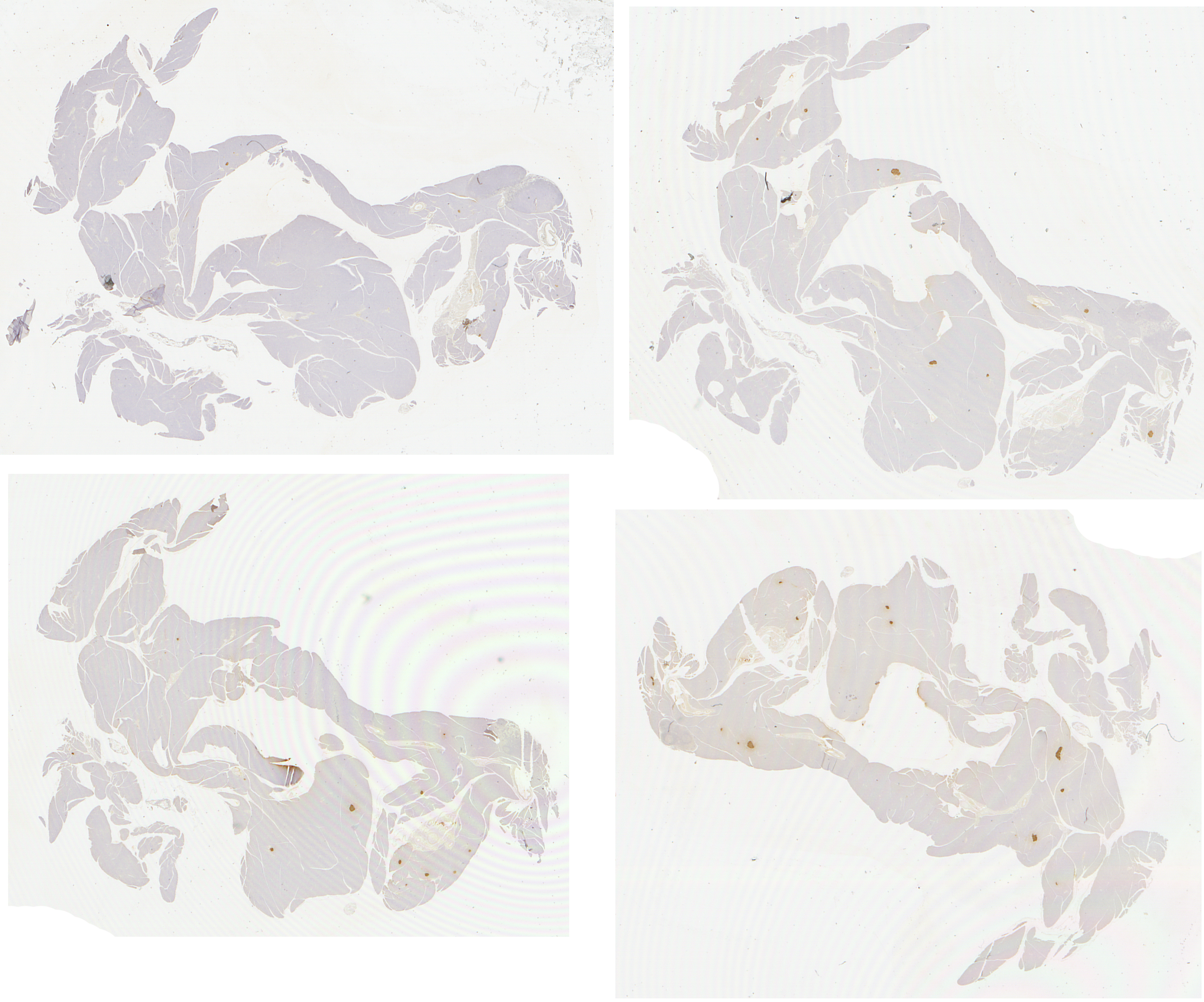

Supplement: Figure 7—source data 1. — The zip file contains all the 2400-dpi scanned images of insulin-immunostained pancreas sections used for quantitation of insulin-positive cell area. Insulin is stained as brown, and the whole section is counterstained as blue with hematoxylin. Folders are named after genotypes (WT or P-AdnTg+) and subfolders after time points (week 0, 2, 5, or 10) post initial dimerizer administration. Related to Figure 7A. DOI: http://dx.doi.org/10.7554/eLife.03851.031 [file elife03851s002.zip › Figure 7-source data 1/P-AdnTg+/Week 10/d25-1s a.jpg]

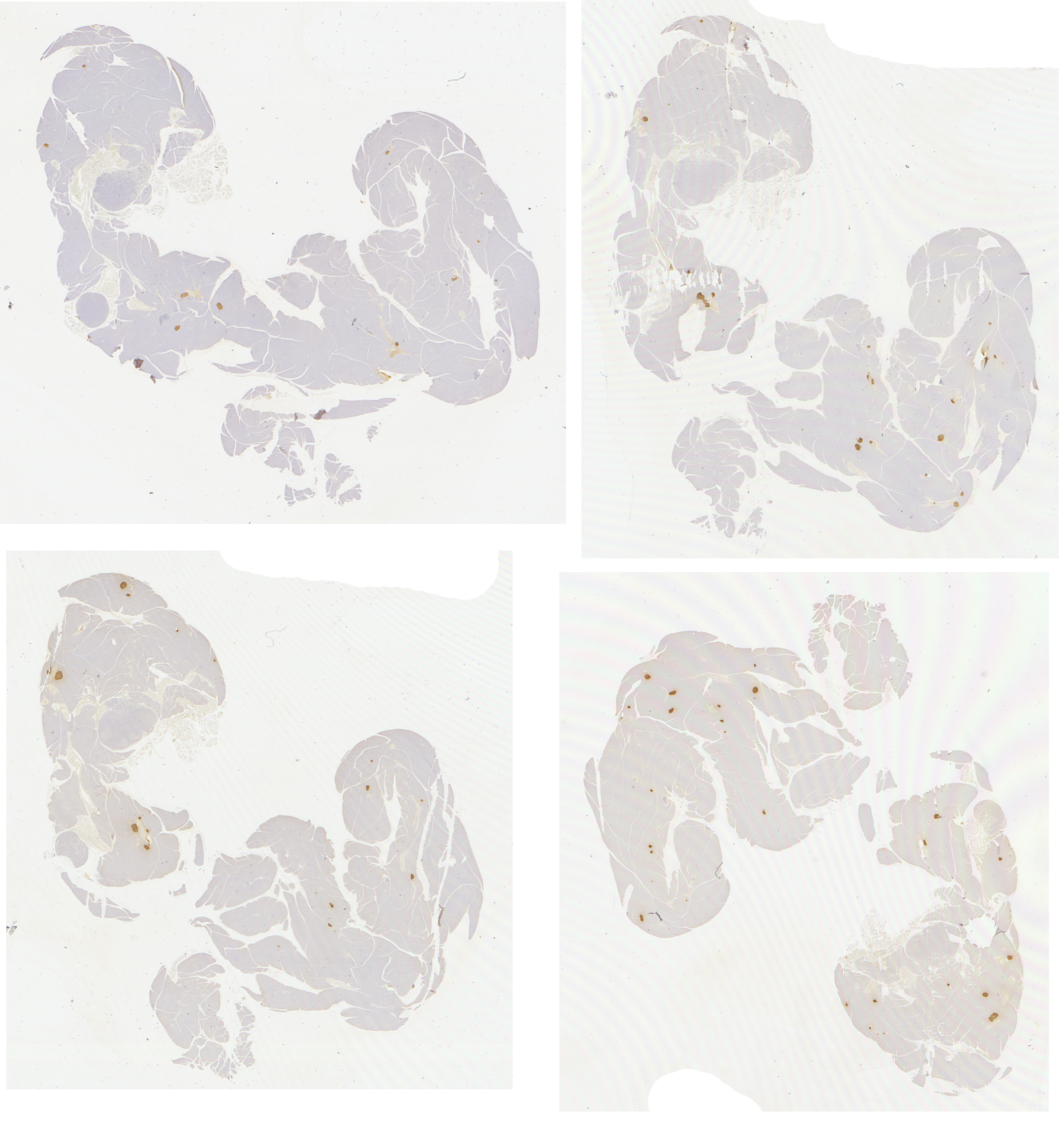

Supplement: Figure 7—source data 1. — The zip file contains all the 2400-dpi scanned images of insulin-immunostained pancreas sections used for quantitation of insulin-positive cell area. Insulin is stained as brown, and the whole section is counterstained as blue with hematoxylin. Folders are named after genotypes (WT or P-AdnTg+) and subfolders after time points (week 0, 2, 5, or 10) post initial dimerizer administration. Related to Figure 7A. DOI: http://dx.doi.org/10.7554/eLife.03851.031 [file elife03851s002.zip › Figure 7-source data 1/P-AdnTg+/Week 10/d25-1s b.jpg]

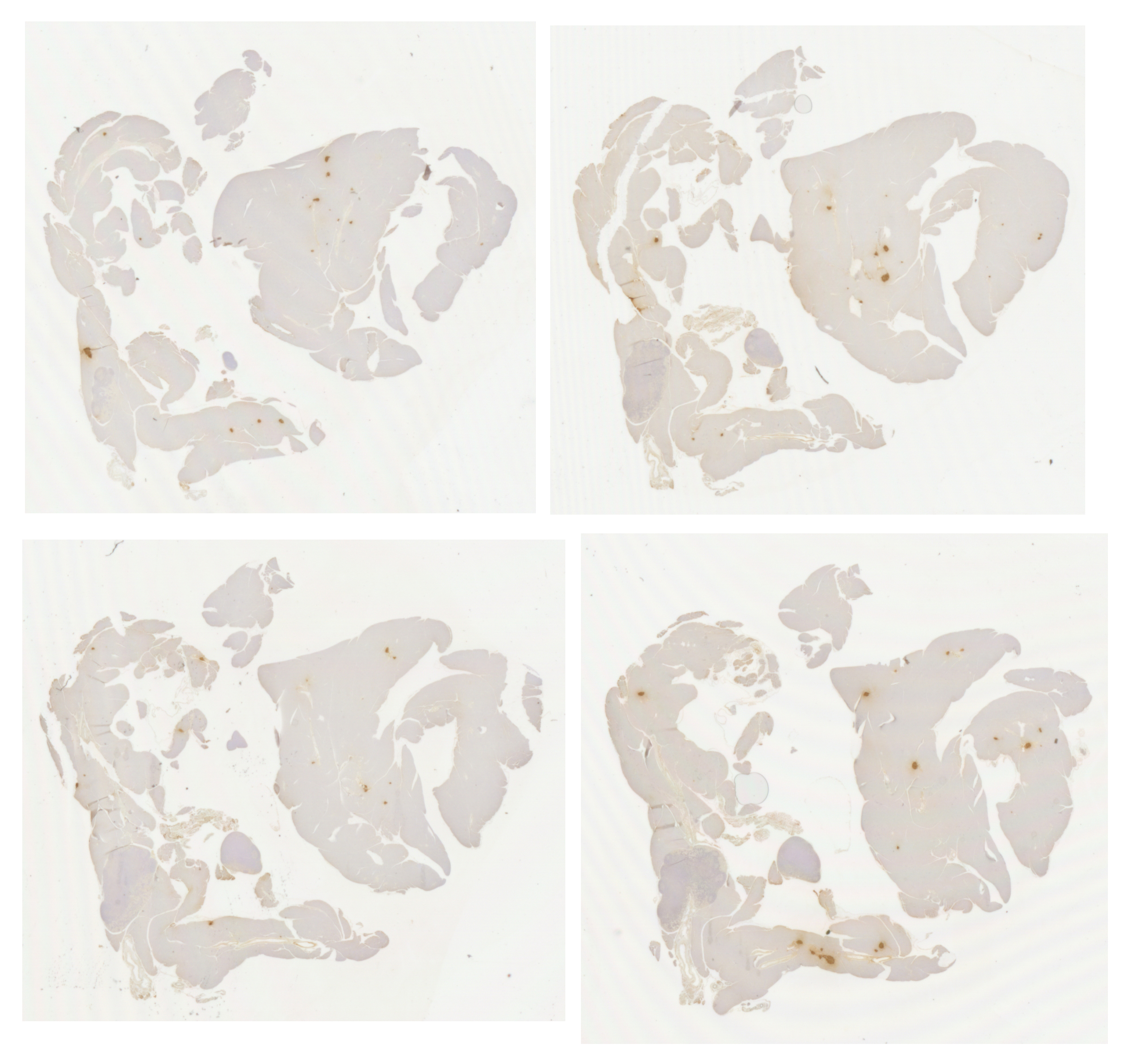

Supplement: Figure 7—source data 1. — The zip file contains all the 2400-dpi scanned images of insulin-immunostained pancreas sections used for quantitation of insulin-positive cell area. Insulin is stained as brown, and the whole section is counterstained as blue with hematoxylin. Folders are named after genotypes (WT or P-AdnTg+) and subfolders after time points (week 0, 2, 5, or 10) post initial dimerizer administration. Related to Figure 7A. DOI: http://dx.doi.org/10.7554/eLife.03851.031 [file elife03851s002.zip › Figure 7-source data 1/P-AdnTg+/Week 10/d41-12 a.jpg]

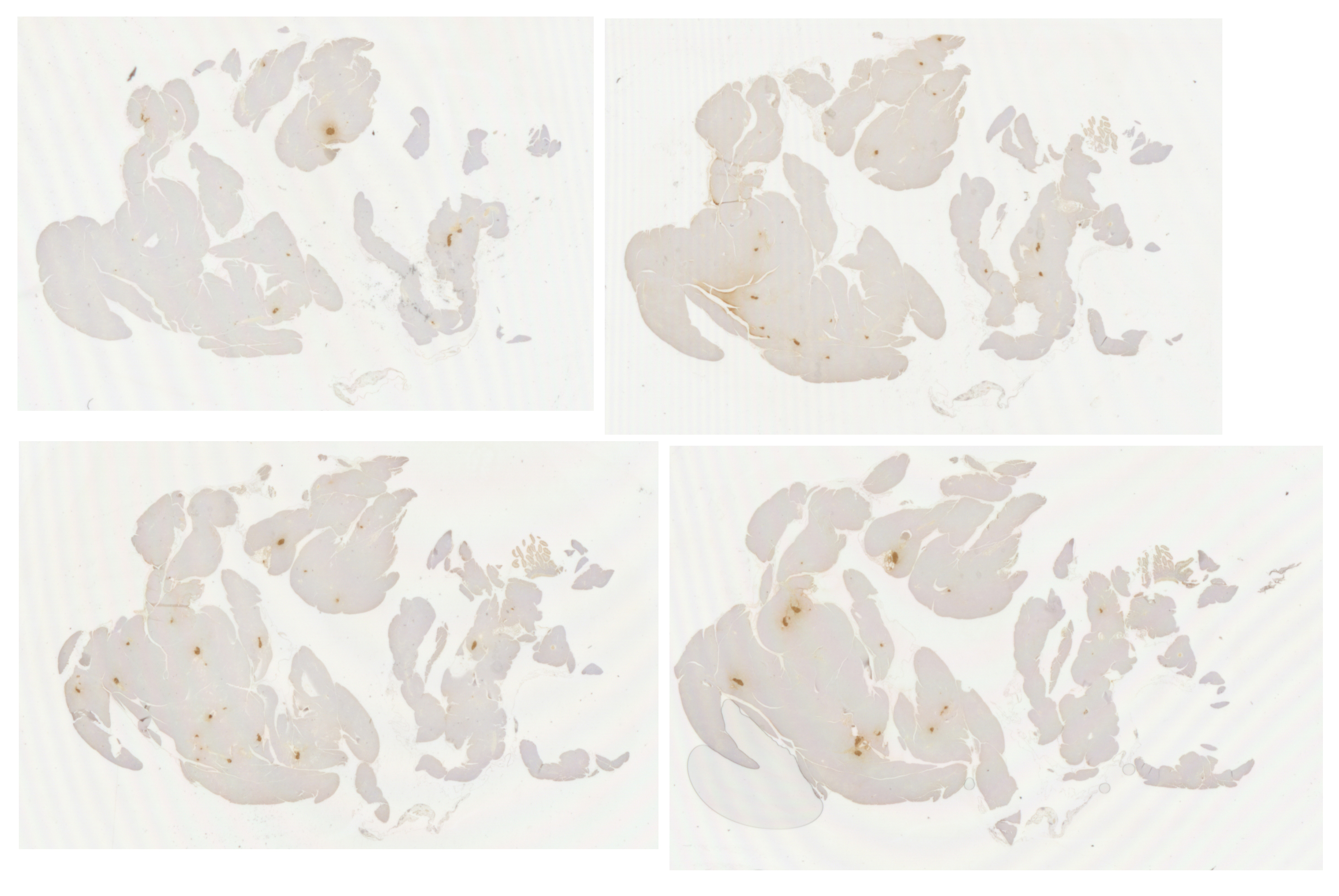

Supplement: Figure 7—source data 1. — The zip file contains all the 2400-dpi scanned images of insulin-immunostained pancreas sections used for quantitation of insulin-positive cell area. Insulin is stained as brown, and the whole section is counterstained as blue with hematoxylin. Folders are named after genotypes (WT or P-AdnTg+) and subfolders after time points (week 0, 2, 5, or 10) post initial dimerizer administration. Related to Figure 7A. DOI: http://dx.doi.org/10.7554/eLife.03851.031 [file elife03851s002.zip › Figure 7-source data 1/P-AdnTg+/Week 10/d41-12 b.jpg]

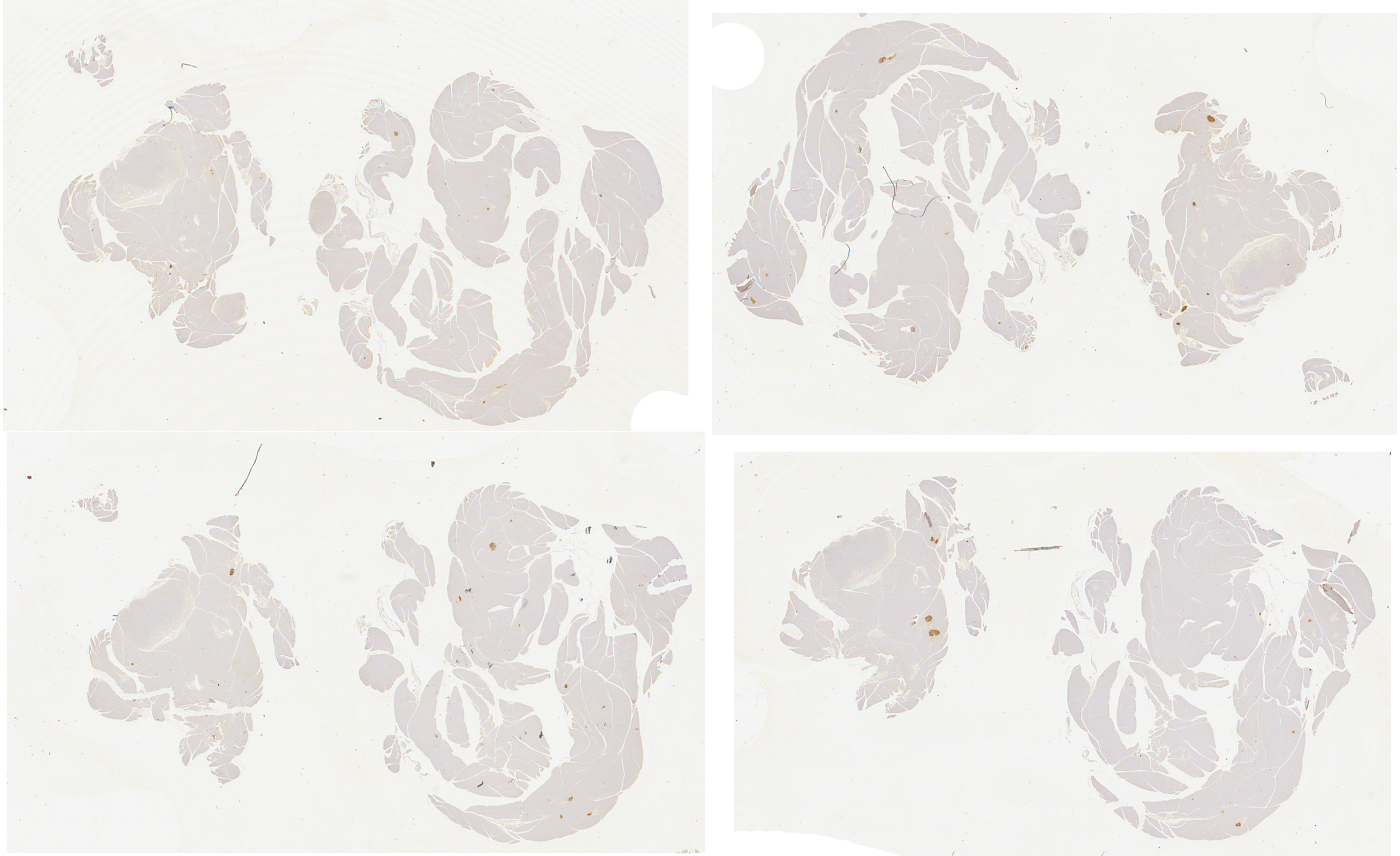

Supplement: Figure 7—source data 1. — The zip file contains all the 2400-dpi scanned images of insulin-immunostained pancreas sections used for quantitation of insulin-positive cell area. Insulin is stained as brown, and the whole section is counterstained as blue with hematoxylin. Folders are named after genotypes (WT or P-AdnTg+) and subfolders after time points (week 0, 2, 5, or 10) post initial dimerizer administration. Related to Figure 7A. DOI: http://dx.doi.org/10.7554/eLife.03851.031 [file elife03851s002.zip › Figure 7-source data 1/P-AdnTg+/Week 2/d45-345 a.jpg]

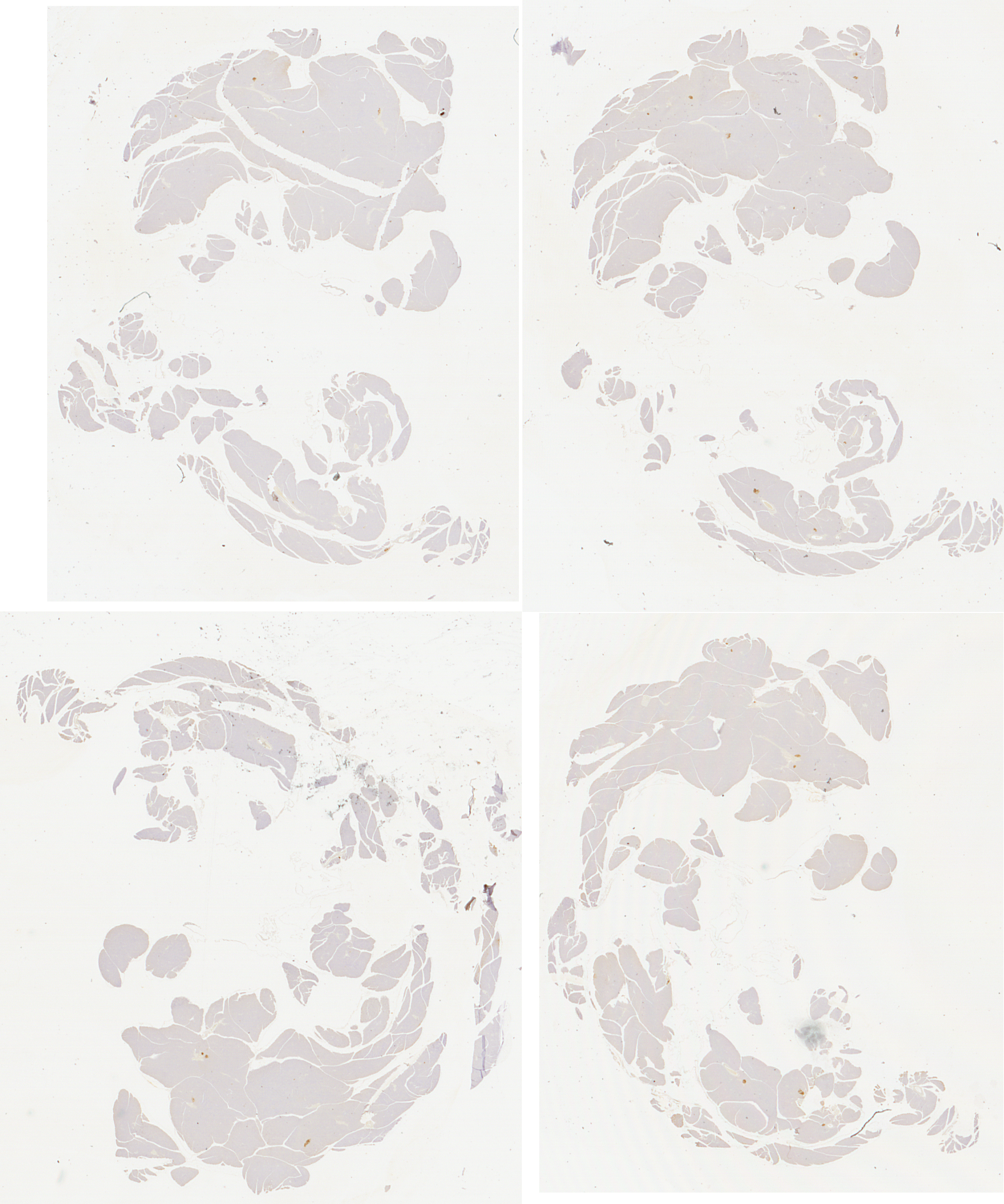

Supplement: Figure 7—source data 1. — The zip file contains all the 2400-dpi scanned images of insulin-immunostained pancreas sections used for quantitation of insulin-positive cell area. Insulin is stained as brown, and the whole section is counterstained as blue with hematoxylin. Folders are named after genotypes (WT or P-AdnTg+) and subfolders after time points (week 0, 2, 5, or 10) post initial dimerizer administration. Related to Figure 7A. DOI: http://dx.doi.org/10.7554/eLife.03851.031 [file elife03851s002.zip › Figure 7-source data 1/P-AdnTg+/Week 2/d45-345 b.jpg]

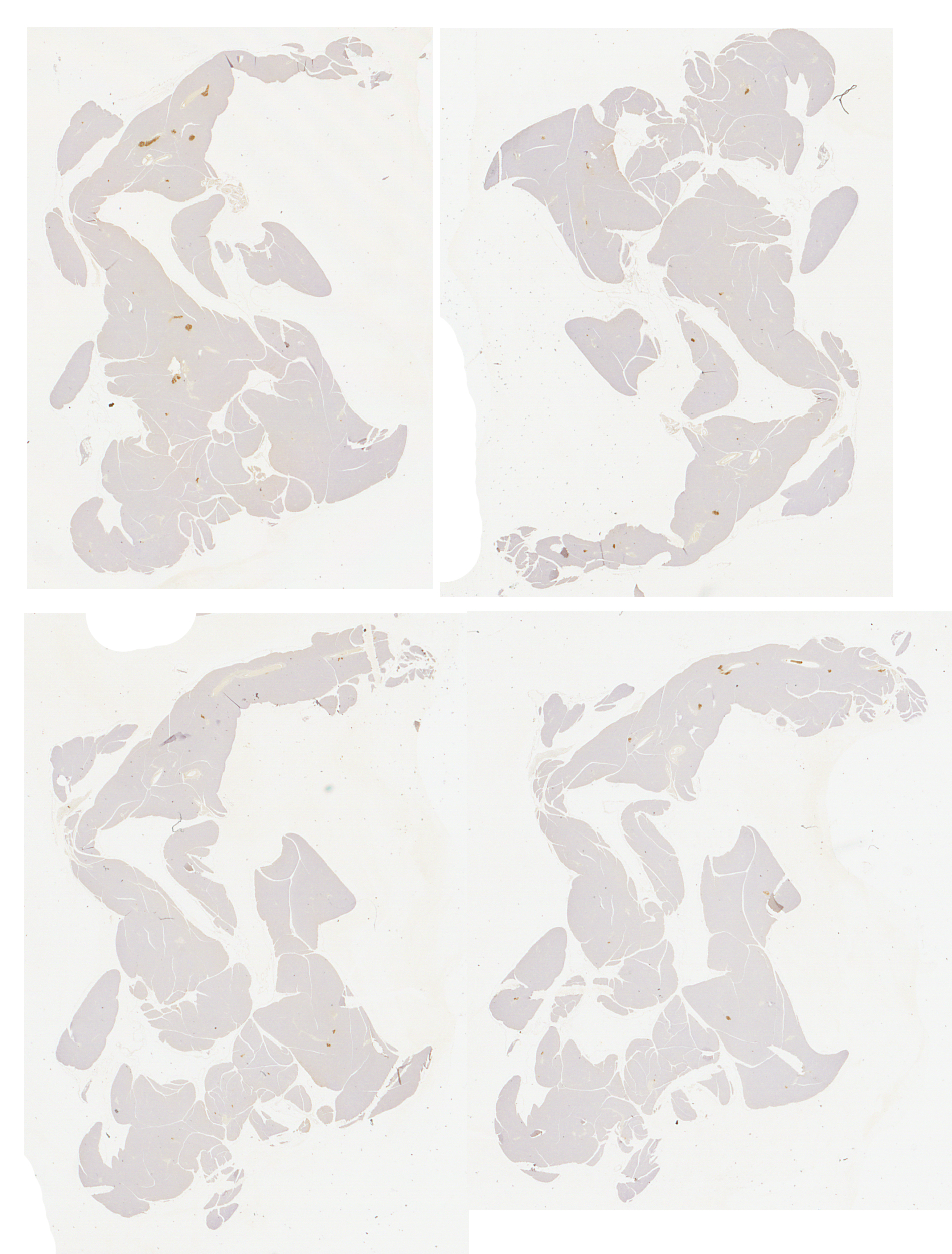

Supplement: Figure 7—source data 1. — The zip file contains all the 2400-dpi scanned images of insulin-immunostained pancreas sections used for quantitation of insulin-positive cell area. Insulin is stained as brown, and the whole section is counterstained as blue with hematoxylin. Folders are named after genotypes (WT or P-AdnTg+) and subfolders after time points (week 0, 2, 5, or 10) post initial dimerizer administration. Related to Figure 7A. DOI: http://dx.doi.org/10.7554/eLife.03851.031 [file elife03851s002.zip › Figure 7-source data 1/P-AdnTg+/Week 2/d45-345 c.jpg]

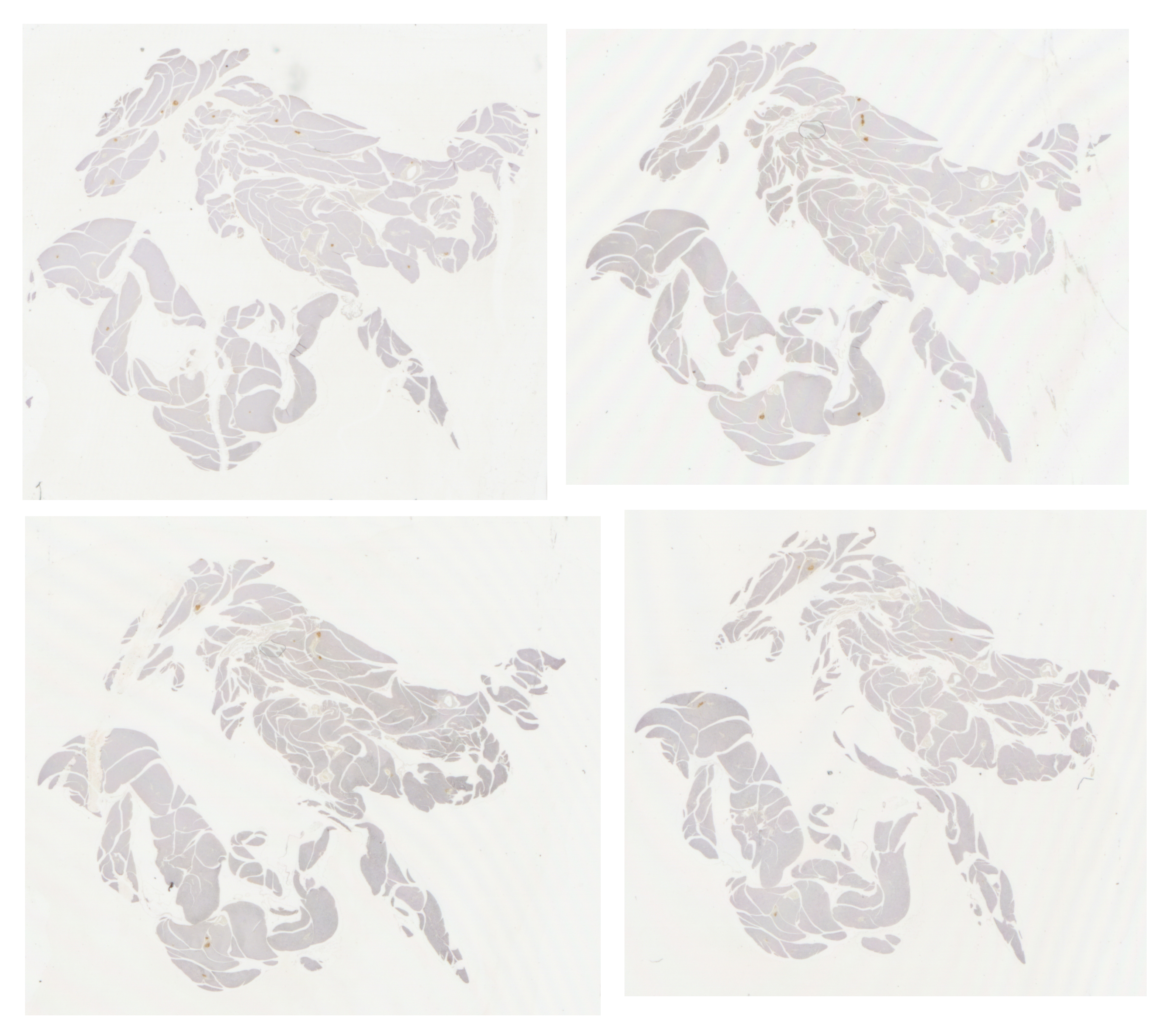

Supplement: Figure 7—source data 1. — The zip file contains all the 2400-dpi scanned images of insulin-immunostained pancreas sections used for quantitation of insulin-positive cell area. Insulin is stained as brown, and the whole section is counterstained as blue with hematoxylin. Folders are named after genotypes (WT or P-AdnTg+) and subfolders after time points (week 0, 2, 5, or 10) post initial dimerizer administration. Related to Figure 7A. DOI: http://dx.doi.org/10.7554/eLife.03851.031 [file elife03851s002.zip › Figure 7-source data 1/P-AdnTg+/Week 2/d46-235 a.jpg]

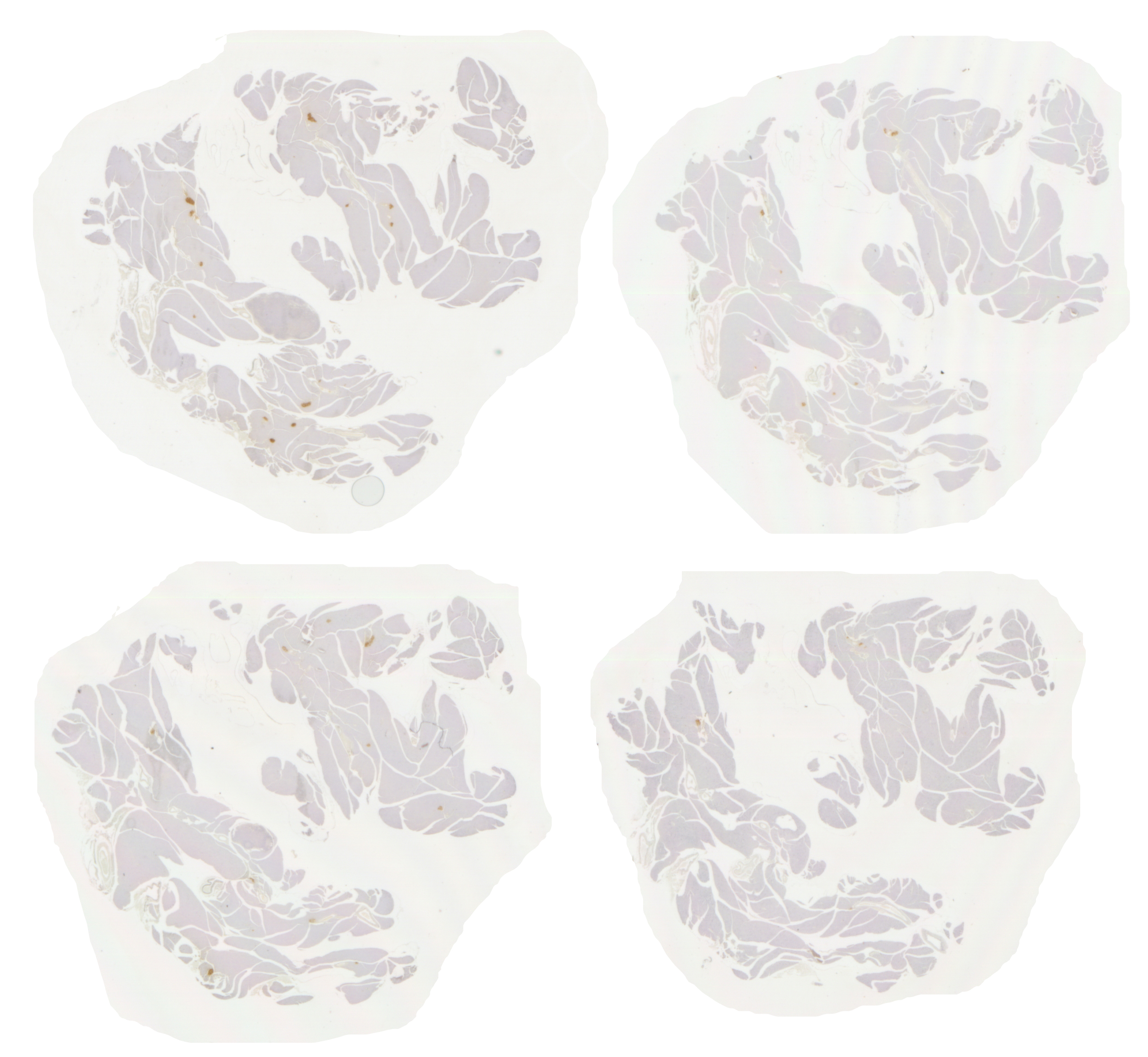

Supplement: Figure 7—source data 1. — The zip file contains all the 2400-dpi scanned images of insulin-immunostained pancreas sections used for quantitation of insulin-positive cell area. Insulin is stained as brown, and the whole section is counterstained as blue with hematoxylin. Folders are named after genotypes (WT or P-AdnTg+) and subfolders after time points (week 0, 2, 5, or 10) post initial dimerizer administration. Related to Figure 7A. DOI: http://dx.doi.org/10.7554/eLife.03851.031 [file elife03851s002.zip › Figure 7-source data 1/P-AdnTg+/Week 2/d46-235 b.jpg]

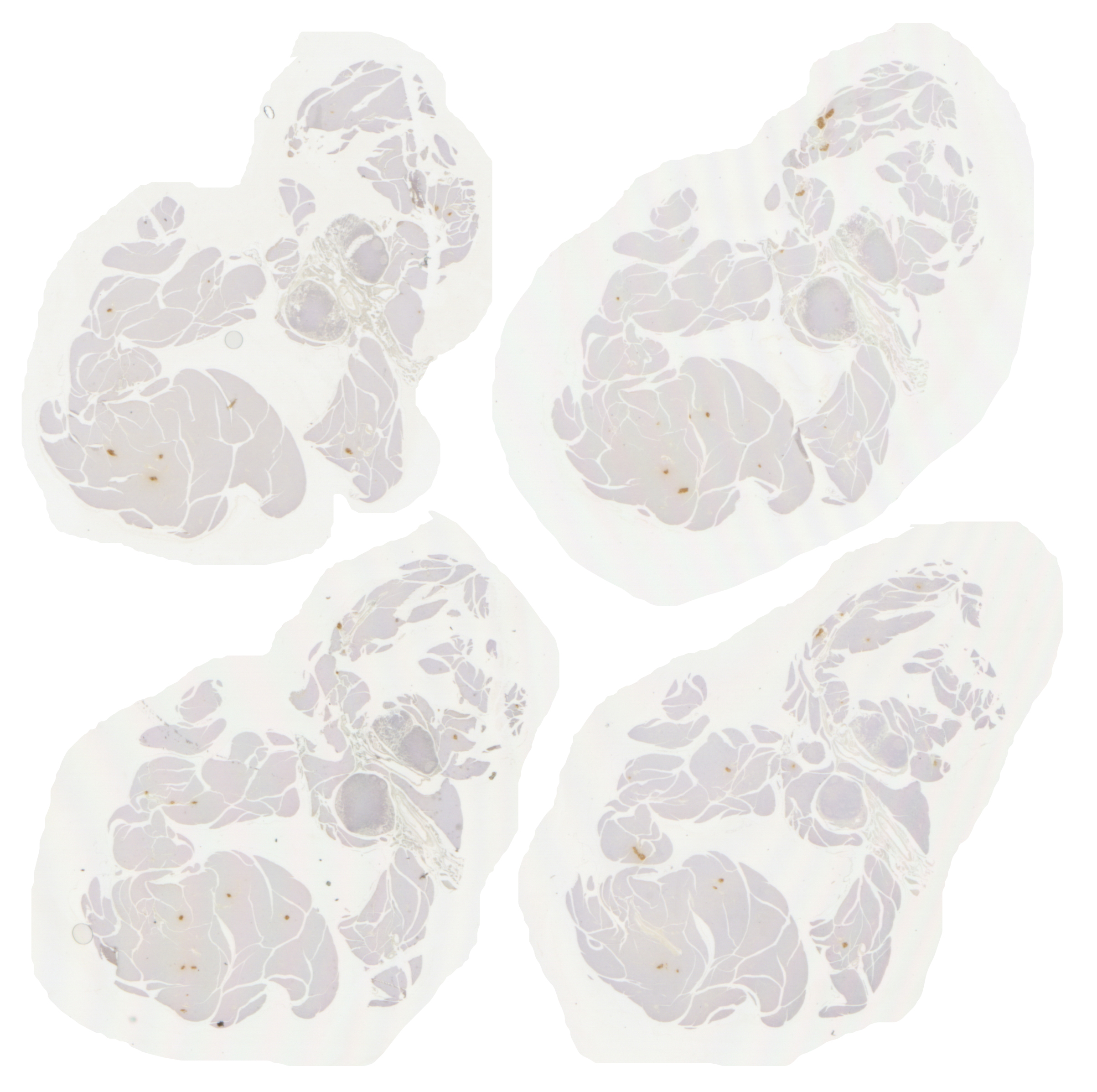

Supplement: Figure 7—source data 1. — The zip file contains all the 2400-dpi scanned images of insulin-immunostained pancreas sections used for quantitation of insulin-positive cell area. Insulin is stained as brown, and the whole section is counterstained as blue with hematoxylin. Folders are named after genotypes (WT or P-AdnTg+) and subfolders after time points (week 0, 2, 5, or 10) post initial dimerizer administration. Related to Figure 7A. DOI: http://dx.doi.org/10.7554/eLife.03851.031 [file elife03851s002.zip › Figure 7-source data 1/P-AdnTg+/Week 2/d46-235 c.jpg]

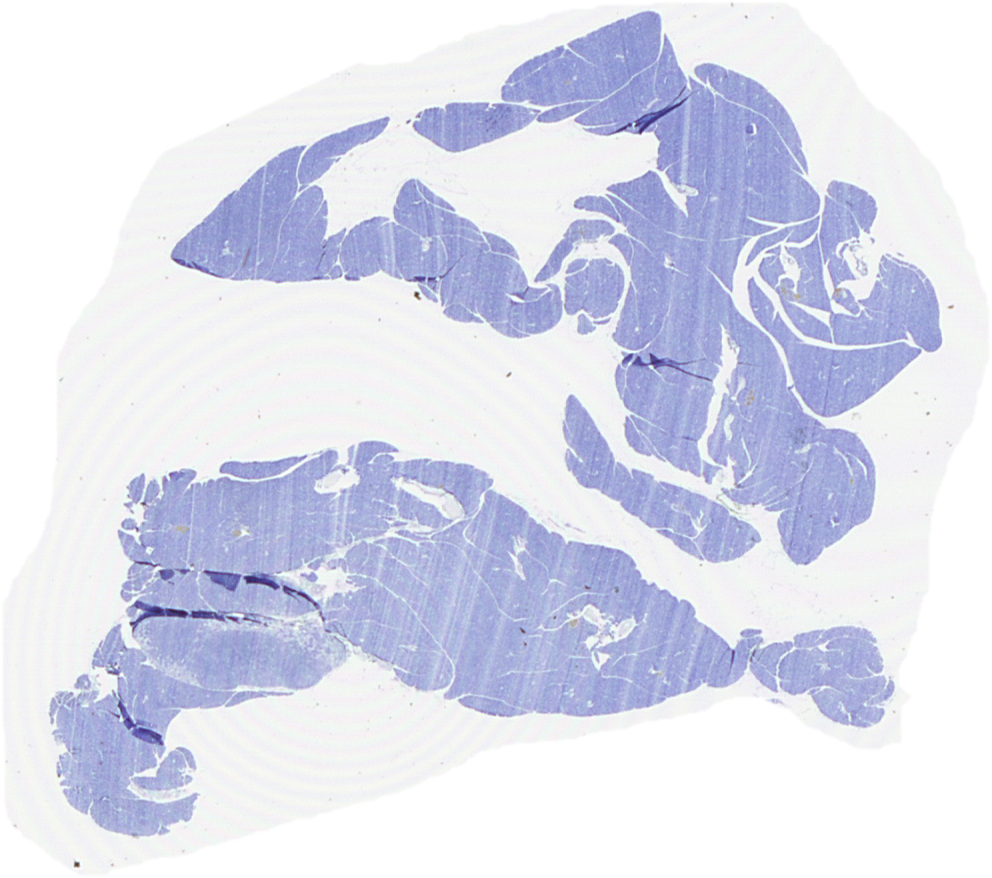

Supplement: Figure 7—source data 1. — The zip file contains all the 2400-dpi scanned images of insulin-immunostained pancreas sections used for quantitation of insulin-positive cell area. Insulin is stained as brown, and the whole section is counterstained as blue with hematoxylin. Folders are named after genotypes (WT or P-AdnTg+) and subfolders after time points (week 0, 2, 5, or 10) post initial dimerizer administration. Related to Figure 7A. DOI: http://dx.doi.org/10.7554/eLife.03851.031 [file elife03851s002.zip › Figure 7-source data 1/P-AdnTg+/Week 5/d55-1s-D3 a.jpg]

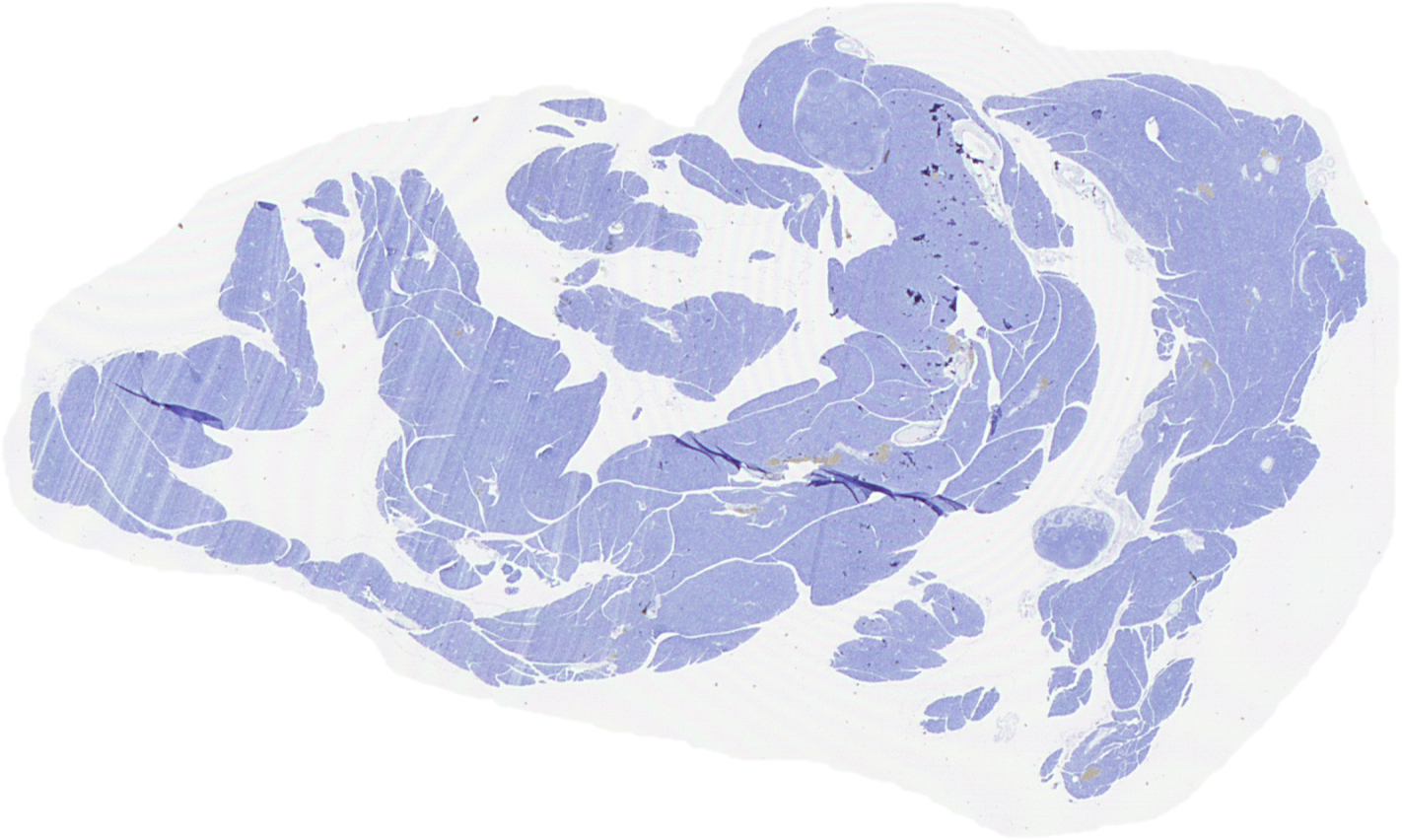

Supplement: Figure 7—source data 1. — The zip file contains all the 2400-dpi scanned images of insulin-immunostained pancreas sections used for quantitation of insulin-positive cell area. Insulin is stained as brown, and the whole section is counterstained as blue with hematoxylin. Folders are named after genotypes (WT or P-AdnTg+) and subfolders after time points (week 0, 2, 5, or 10) post initial dimerizer administration. Related to Figure 7A. DOI: http://dx.doi.org/10.7554/eLife.03851.031 [file elife03851s002.zip › Figure 7-source data 1/P-AdnTg+/Week 5/d55-1s-D3 b.jpg]

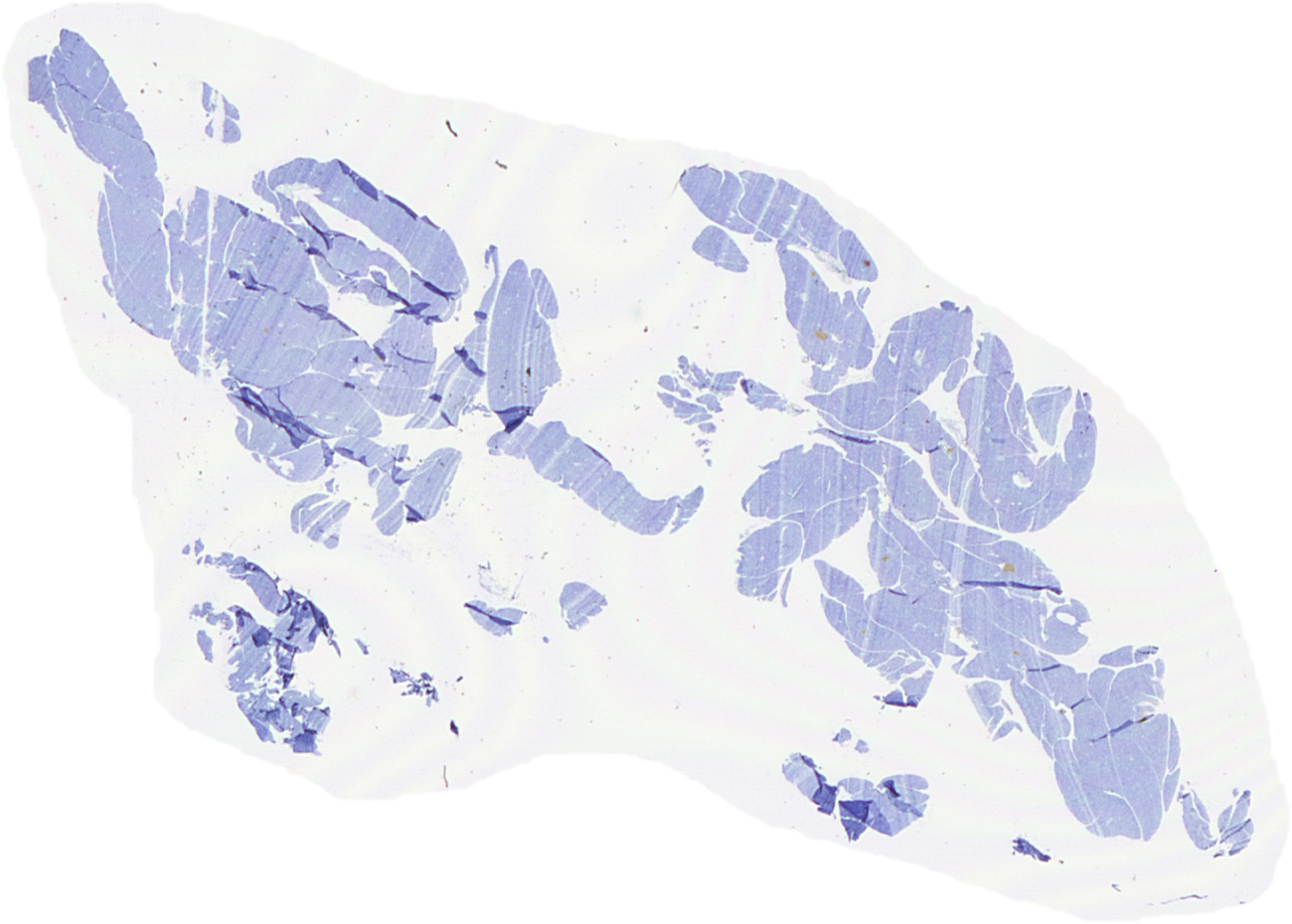

Supplement: Figure 7—source data 1. — The zip file contains all the 2400-dpi scanned images of insulin-immunostained pancreas sections used for quantitation of insulin-positive cell area. Insulin is stained as brown, and the whole section is counterstained as blue with hematoxylin. Folders are named after genotypes (WT or P-AdnTg+) and subfolders after time points (week 0, 2, 5, or 10) post initial dimerizer administration. Related to Figure 7A. DOI: http://dx.doi.org/10.7554/eLife.03851.031 [file elife03851s002.zip › Figure 7-source data 1/P-AdnTg+/Week 5/d55-1s-D3 c.jpg]

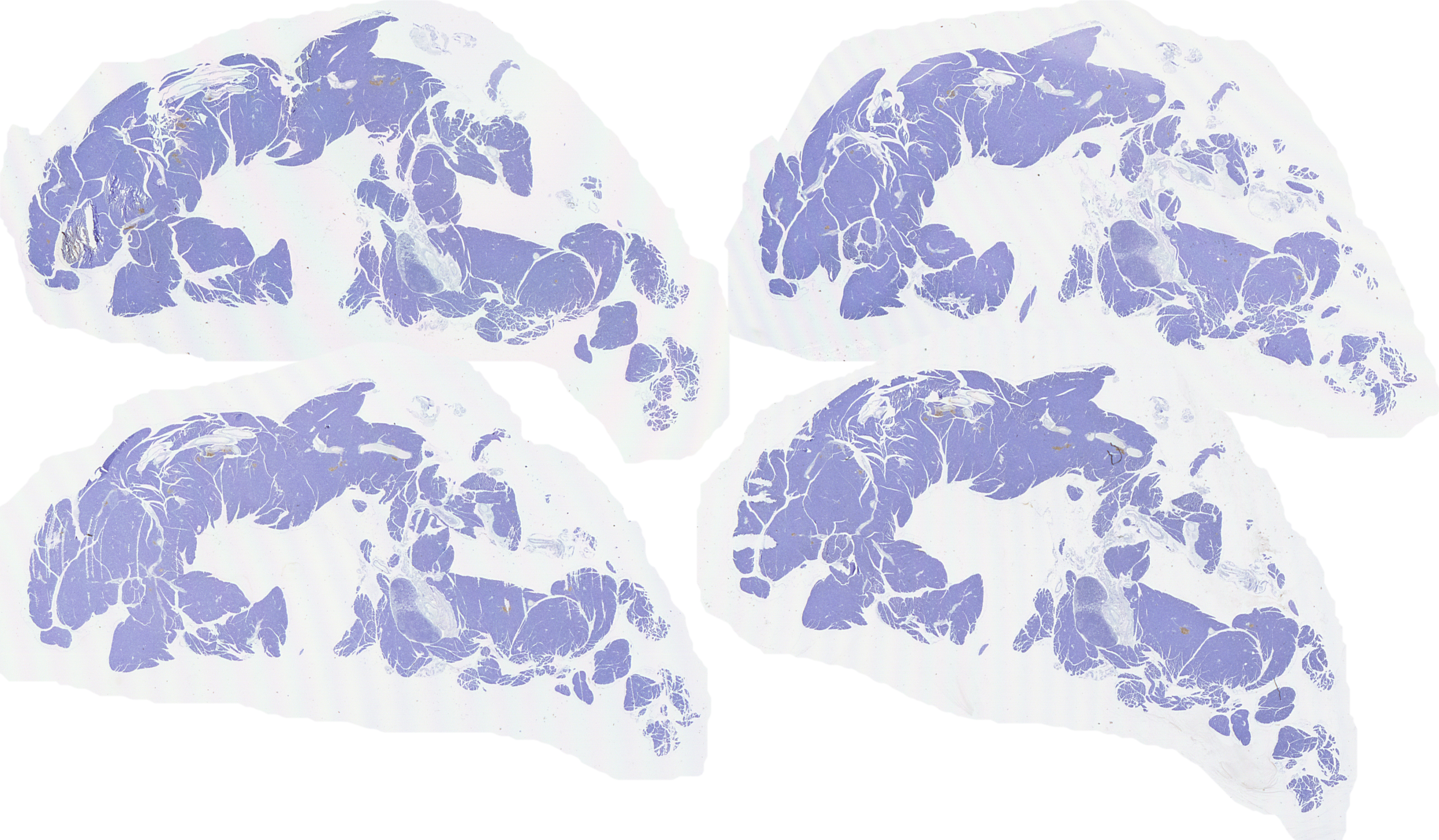

Supplement: Figure 7—source data 1. — The zip file contains all the 2400-dpi scanned images of insulin-immunostained pancreas sections used for quantitation of insulin-positive cell area. Insulin is stained as brown, and the whole section is counterstained as blue with hematoxylin. Folders are named after genotypes (WT or P-AdnTg+) and subfolders after time points (week 0, 2, 5, or 10) post initial dimerizer administration. Related to Figure 7A. DOI: http://dx.doi.org/10.7554/eLife.03851.031 [file elife03851s002.zip › Figure 7-source data 1/P-AdnTg+/Week 5/d67-3s a.jpg]

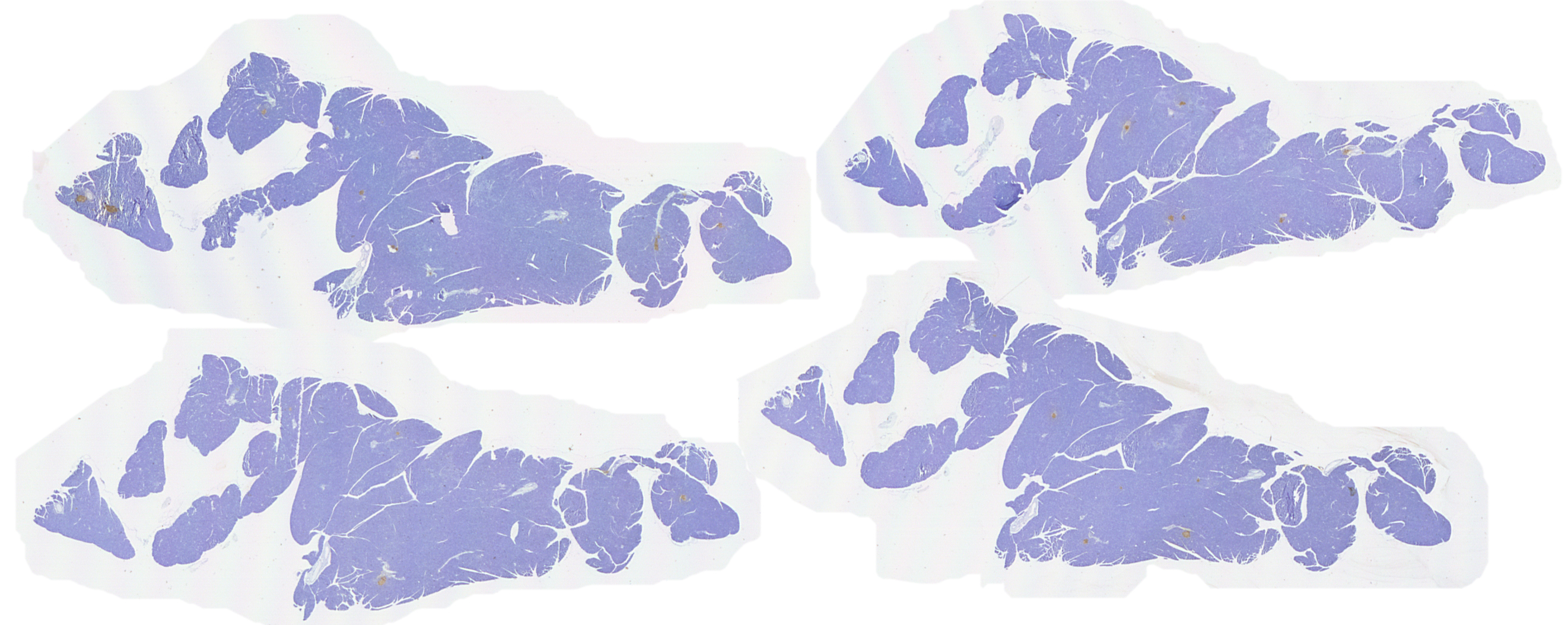

Supplement: Figure 7—source data 1. — The zip file contains all the 2400-dpi scanned images of insulin-immunostained pancreas sections used for quantitation of insulin-positive cell area. Insulin is stained as brown, and the whole section is counterstained as blue with hematoxylin. Folders are named after genotypes (WT or P-AdnTg+) and subfolders after time points (week 0, 2, 5, or 10) post initial dimerizer administration. Related to Figure 7A. DOI: http://dx.doi.org/10.7554/eLife.03851.031 [file elife03851s002.zip › Figure 7-source data 1/P-AdnTg+/Week 5/d67-3s b.jpg]

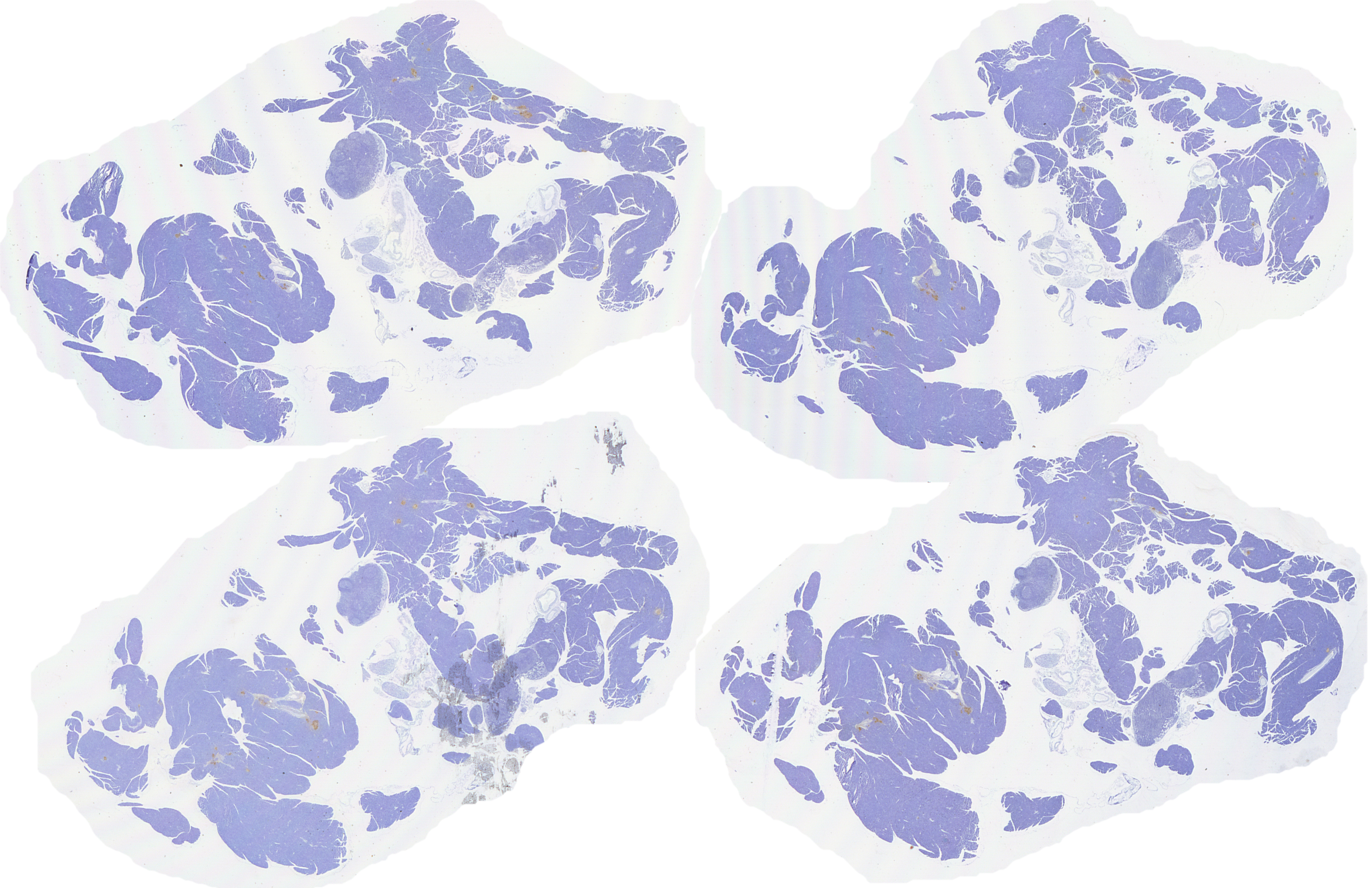

Supplement: Figure 7—source data 1. — The zip file contains all the 2400-dpi scanned images of insulin-immunostained pancreas sections used for quantitation of insulin-positive cell area. Insulin is stained as brown, and the whole section is counterstained as blue with hematoxylin. Folders are named after genotypes (WT or P-AdnTg+) and subfolders after time points (week 0, 2, 5, or 10) post initial dimerizer administration. Related to Figure 7A. DOI: http://dx.doi.org/10.7554/eLife.03851.031 [file elife03851s002.zip › Figure 7-source data 1/P-AdnTg+/Week 5/d67-3s c.jpg]

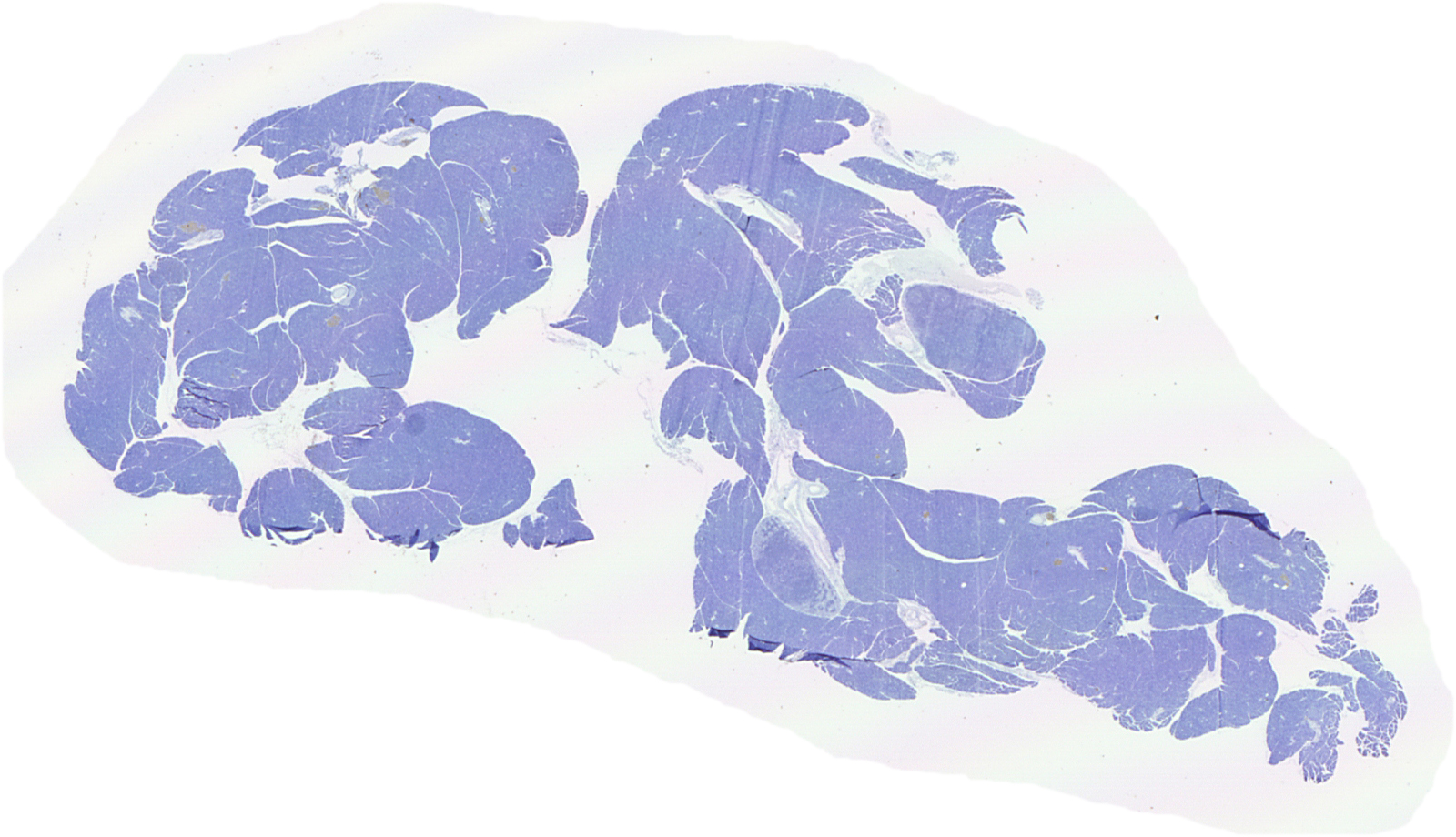

Supplement: Figure 7—source data 1. — The zip file contains all the 2400-dpi scanned images of insulin-immunostained pancreas sections used for quantitation of insulin-positive cell area. Insulin is stained as brown, and the whole section is counterstained as blue with hematoxylin. Folders are named after genotypes (WT or P-AdnTg+) and subfolders after time points (week 0, 2, 5, or 10) post initial dimerizer administration. Related to Figure 7A. DOI: http://dx.doi.org/10.7554/eLife.03851.031 [file elife03851s002.zip › Figure 7-source data 1/P-AdnTg+/Week 5/d67-3s-A8 a.jpg]

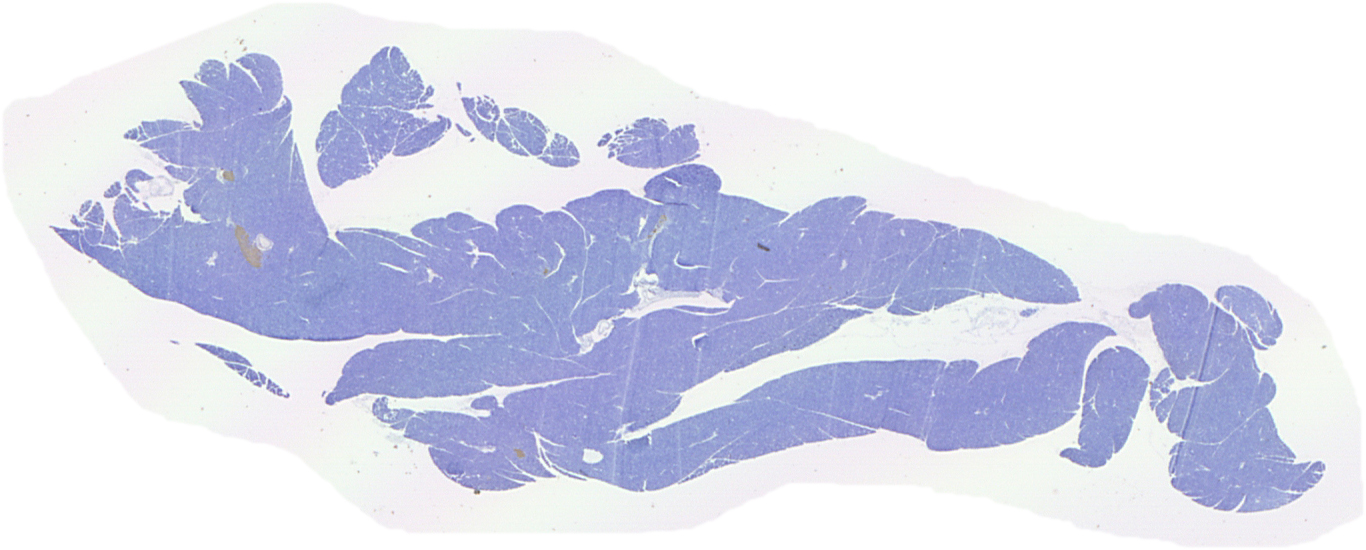

Supplement: Figure 7—source data 1. — The zip file contains all the 2400-dpi scanned images of insulin-immunostained pancreas sections used for quantitation of insulin-positive cell area. Insulin is stained as brown, and the whole section is counterstained as blue with hematoxylin. Folders are named after genotypes (WT or P-AdnTg+) and subfolders after time points (week 0, 2, 5, or 10) post initial dimerizer administration. Related to Figure 7A. DOI: http://dx.doi.org/10.7554/eLife.03851.031 [file elife03851s002.zip › Figure 7-source data 1/P-AdnTg+/Week 5/d67-3s-A8 b.jpg]

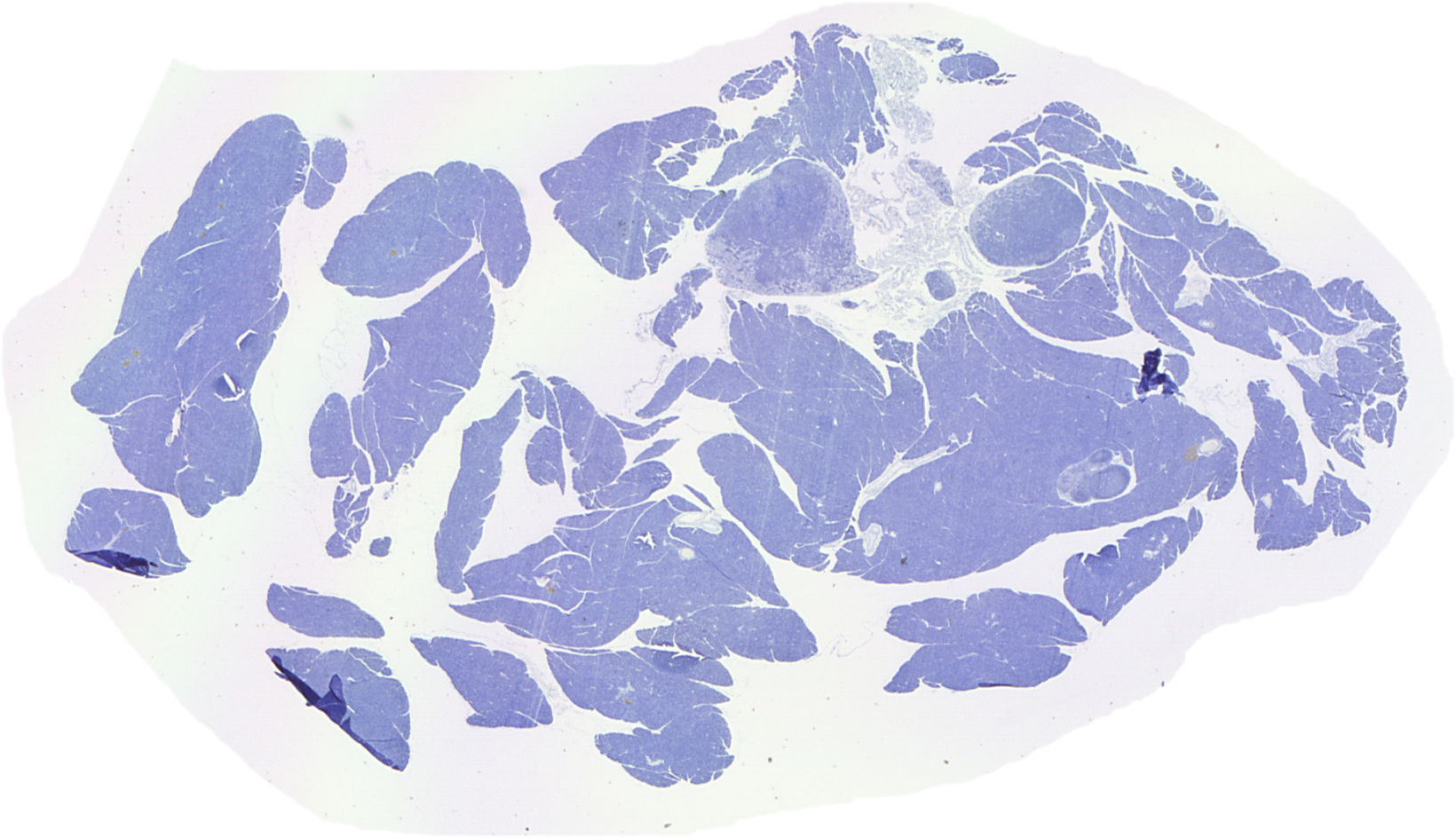

Supplement: Figure 7—source data 1. — The zip file contains all the 2400-dpi scanned images of insulin-immunostained pancreas sections used for quantitation of insulin-positive cell area. Insulin is stained as brown, and the whole section is counterstained as blue with hematoxylin. Folders are named after genotypes (WT or P-AdnTg+) and subfolders after time points (week 0, 2, 5, or 10) post initial dimerizer administration. Related to Figure 7A. DOI: http://dx.doi.org/10.7554/eLife.03851.031 [file elife03851s002.zip › Figure 7-source data 1/P-AdnTg+/Week 5/d67-3s-A8 c.jpg]

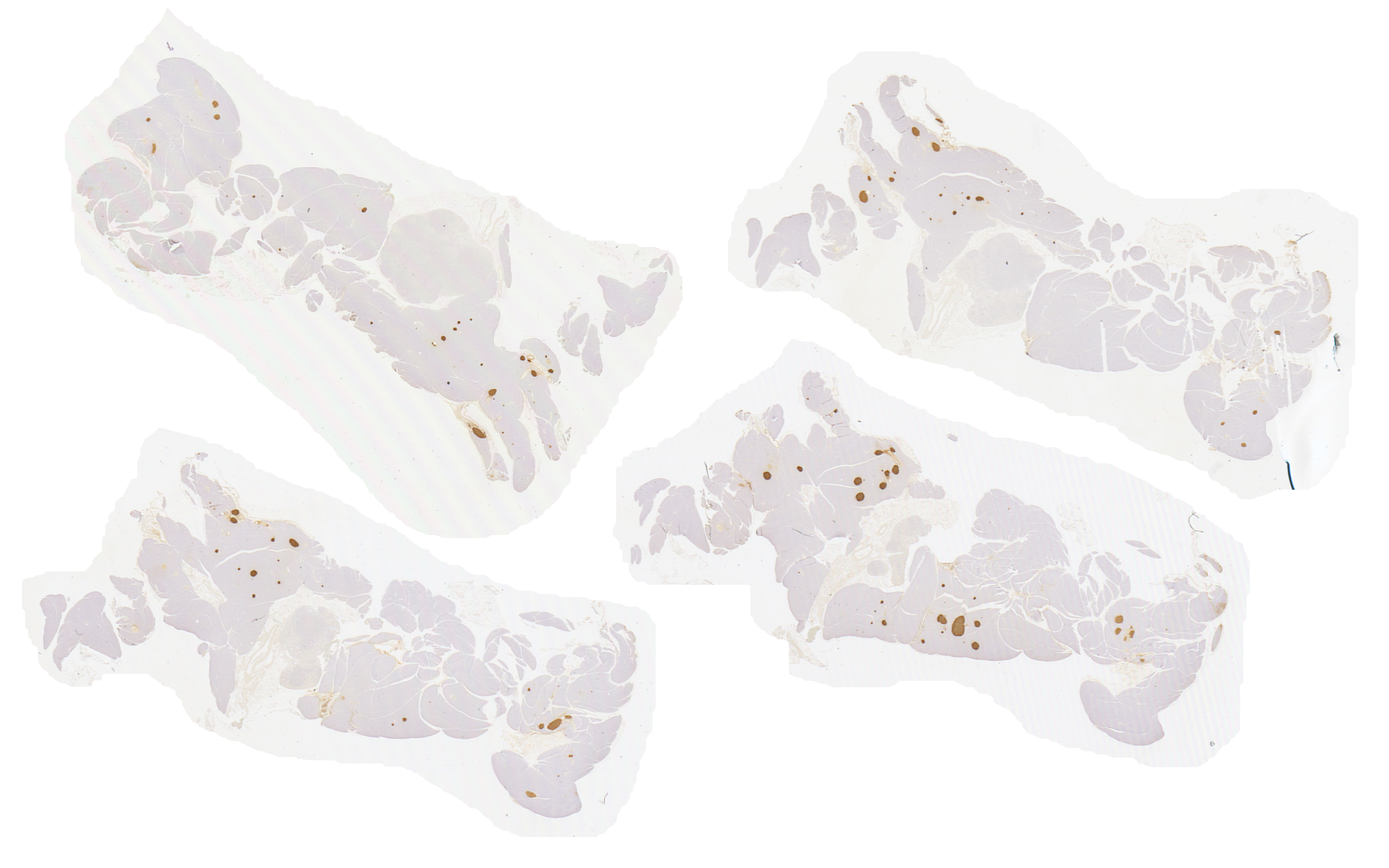

Supplement: Figure 7—source data 1. — The zip file contains all the 2400-dpi scanned images of insulin-immunostained pancreas sections used for quantitation of insulin-positive cell area. Insulin is stained as brown, and the whole section is counterstained as blue with hematoxylin. Folders are named after genotypes (WT or P-AdnTg+) and subfolders after time points (week 0, 2, 5, or 10) post initial dimerizer administration. Related to Figure 7A. DOI: http://dx.doi.org/10.7554/eLife.03851.031 [file elife03851s002.zip › Figure 7-source data 1/WT/Week 0/PA51 a.jpg]

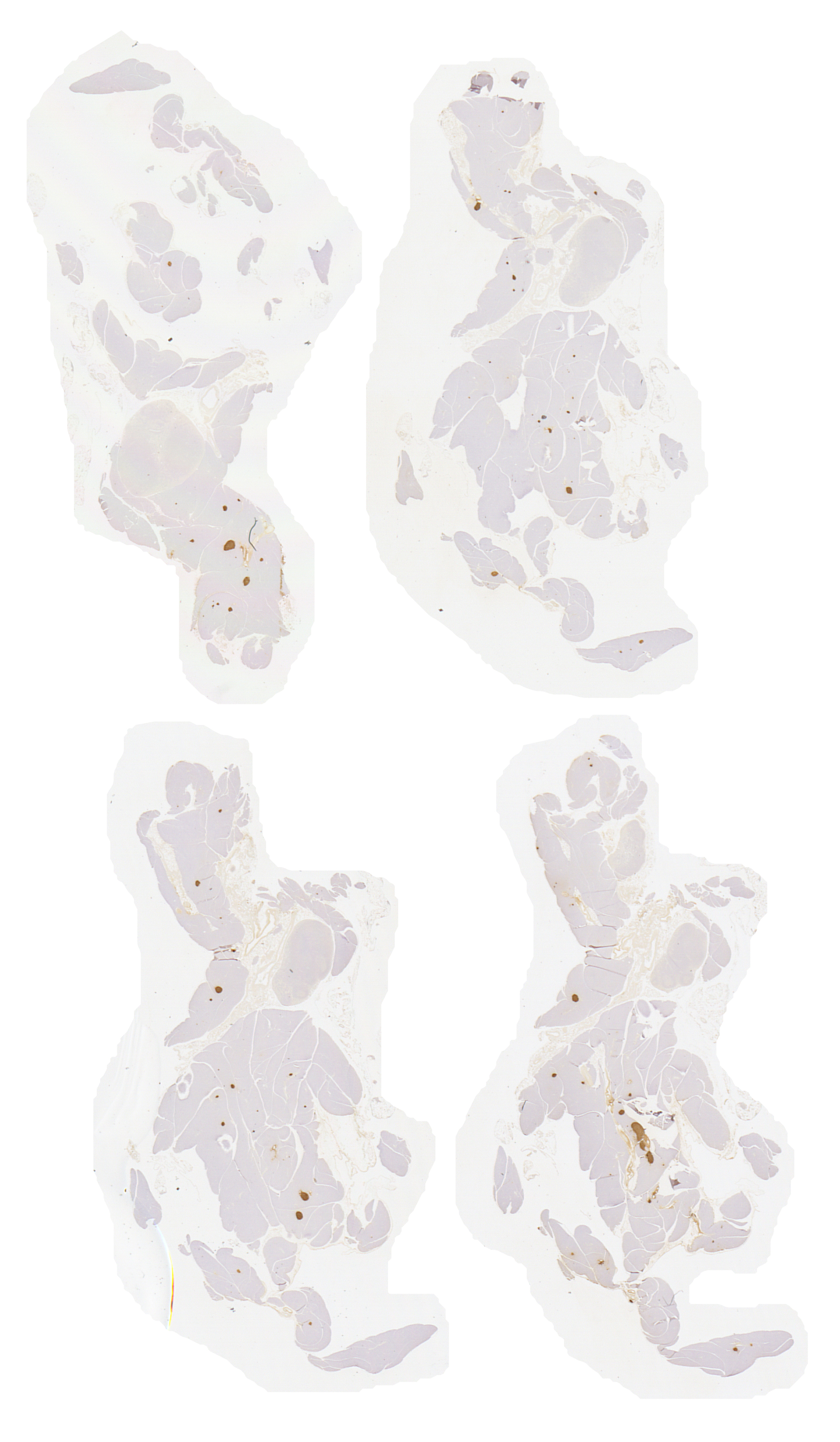

Supplement: Figure 7—source data 1. — The zip file contains all the 2400-dpi scanned images of insulin-immunostained pancreas sections used for quantitation of insulin-positive cell area. Insulin is stained as brown, and the whole section is counterstained as blue with hematoxylin. Folders are named after genotypes (WT or P-AdnTg+) and subfolders after time points (week 0, 2, 5, or 10) post initial dimerizer administration. Related to Figure 7A. DOI: http://dx.doi.org/10.7554/eLife.03851.031 [file elife03851s002.zip › Figure 7-source data 1/WT/Week 0/PA51 b.jpg]

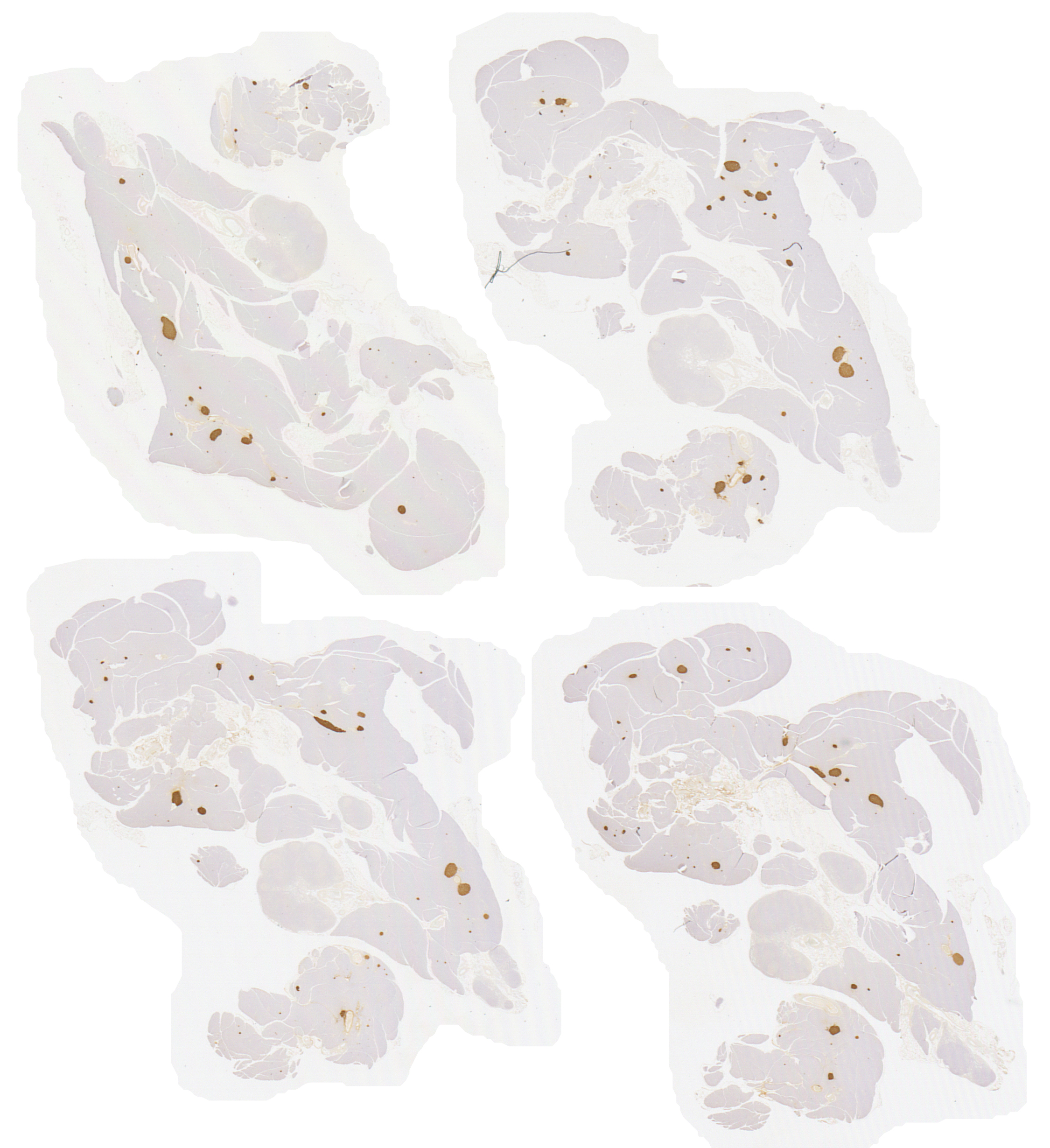

Supplement: Figure 7—source data 1. — The zip file contains all the 2400-dpi scanned images of insulin-immunostained pancreas sections used for quantitation of insulin-positive cell area. Insulin is stained as brown, and the whole section is counterstained as blue with hematoxylin. Folders are named after genotypes (WT or P-AdnTg+) and subfolders after time points (week 0, 2, 5, or 10) post initial dimerizer administration. Related to Figure 7A. DOI: http://dx.doi.org/10.7554/eLife.03851.031 [file elife03851s002.zip › Figure 7-source data 1/WT/Week 0/PA51 c.jpg]

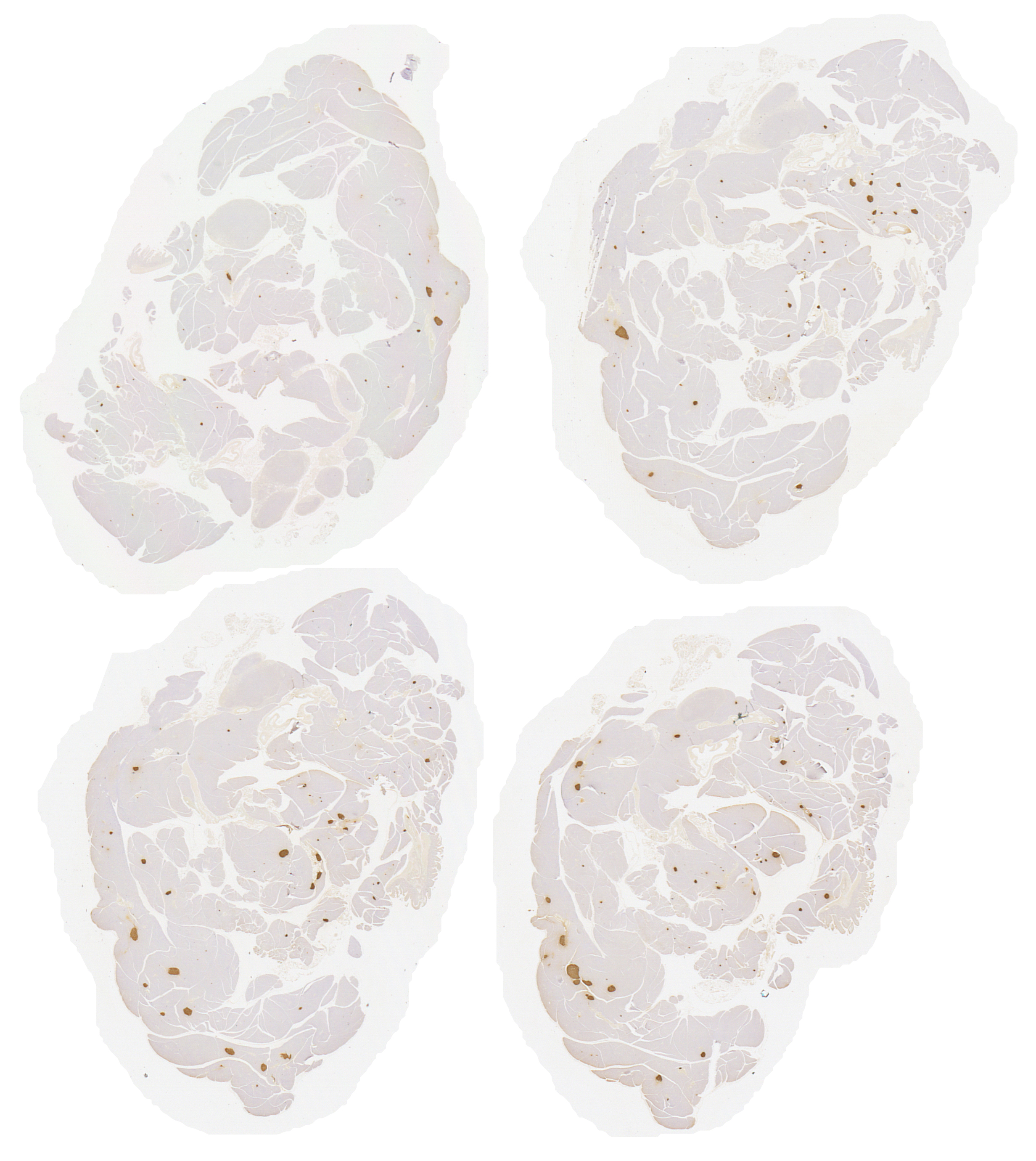

Supplement: Figure 7—source data 1. — The zip file contains all the 2400-dpi scanned images of insulin-immunostained pancreas sections used for quantitation of insulin-positive cell area. Insulin is stained as brown, and the whole section is counterstained as blue with hematoxylin. Folders are named after genotypes (WT or P-AdnTg+) and subfolders after time points (week 0, 2, 5, or 10) post initial dimerizer administration. Related to Figure 7A. DOI: http://dx.doi.org/10.7554/eLife.03851.031 [file elife03851s002.zip › Figure 7-source data 1/WT/Week 0/PA51 d.jpg]

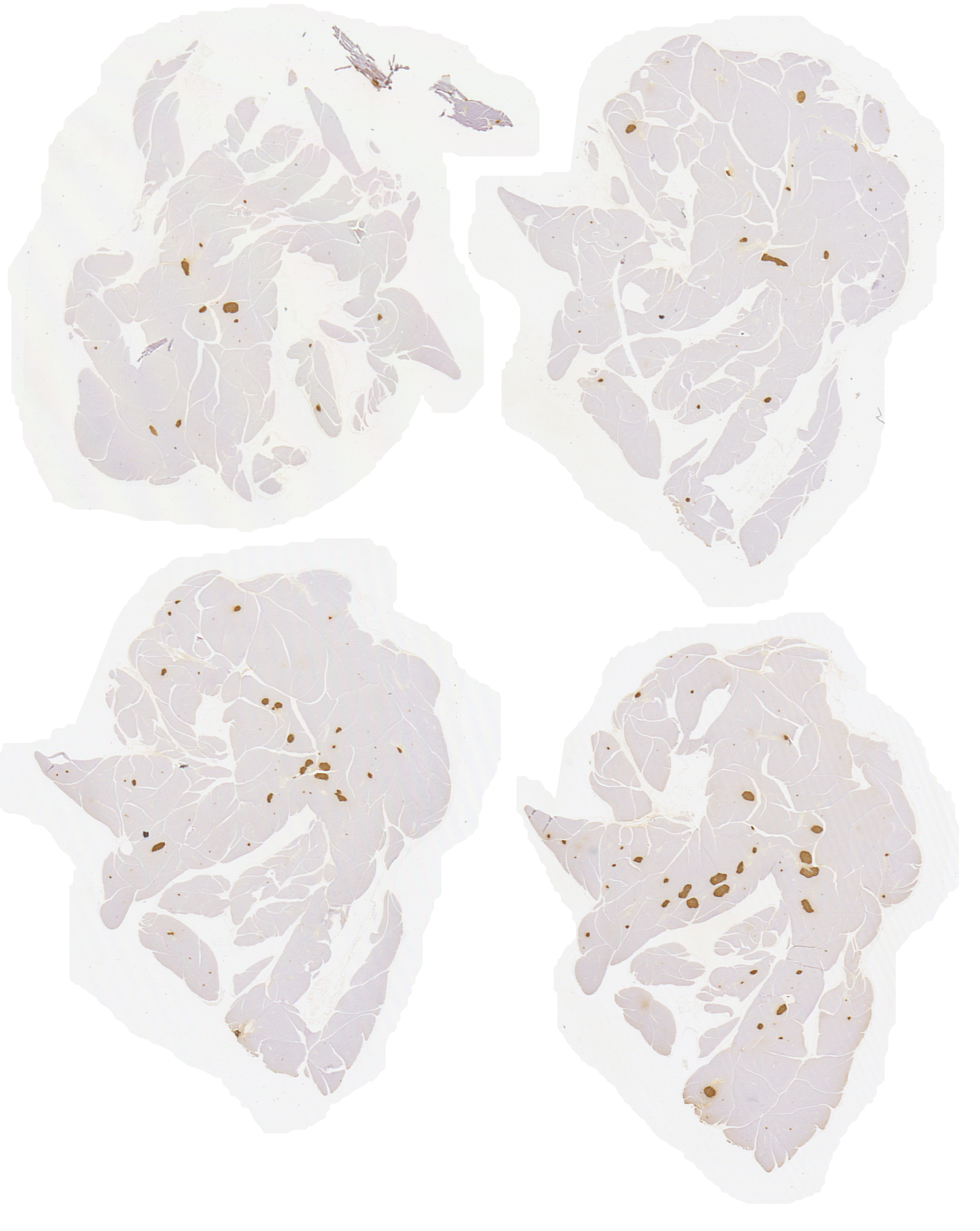

Supplement: Figure 7—source data 1. — The zip file contains all the 2400-dpi scanned images of insulin-immunostained pancreas sections used for quantitation of insulin-positive cell area. Insulin is stained as brown, and the whole section is counterstained as blue with hematoxylin. Folders are named after genotypes (WT or P-AdnTg+) and subfolders after time points (week 0, 2, 5, or 10) post initial dimerizer administration. Related to Figure 7A. DOI: http://dx.doi.org/10.7554/eLife.03851.031 [file elife03851s002.zip › Figure 7-source data 1/WT/Week 0/PA51 e.jpg]

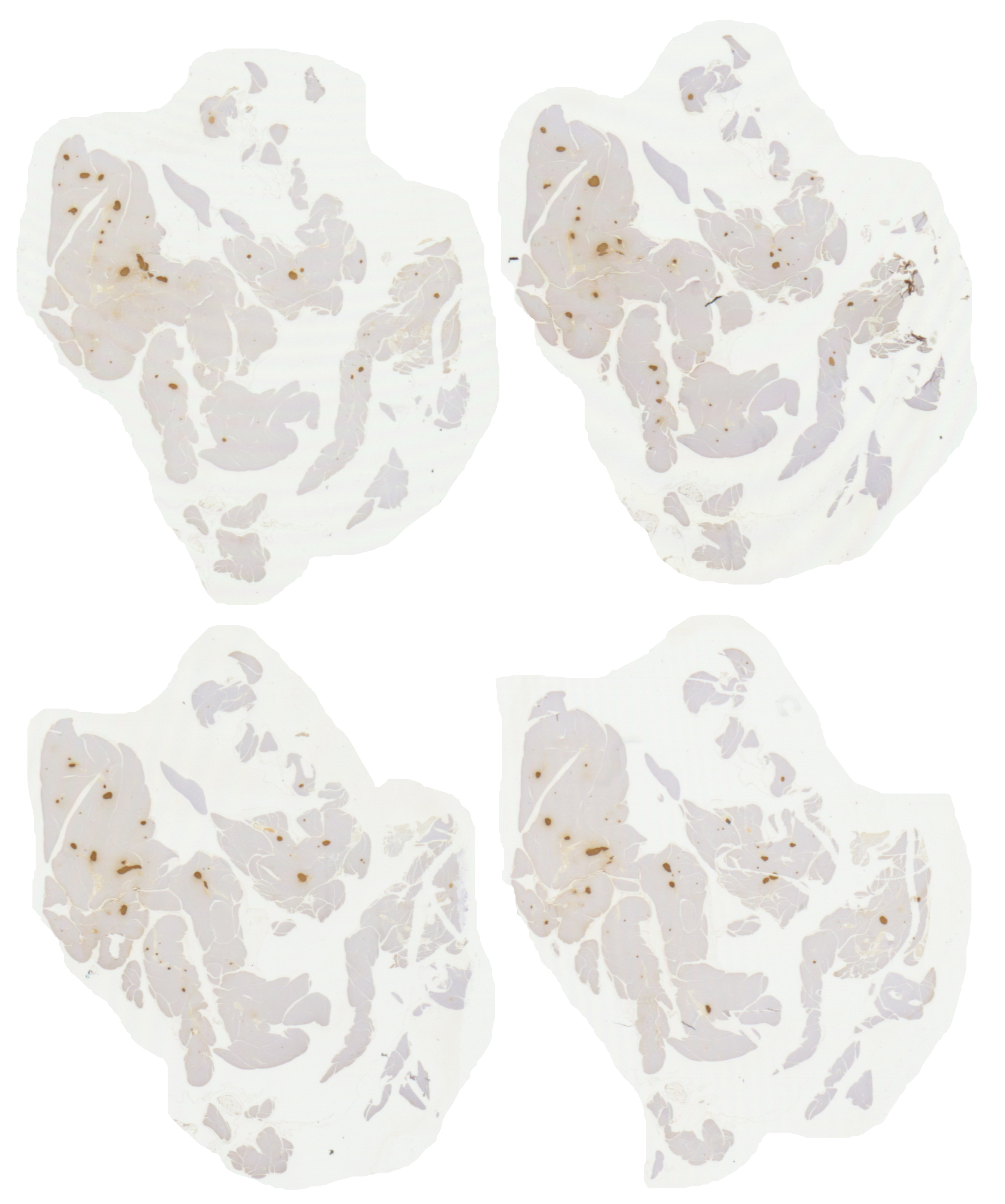

Supplement: Figure 7—source data 1. — The zip file contains all the 2400-dpi scanned images of insulin-immunostained pancreas sections used for quantitation of insulin-positive cell area. Insulin is stained as brown, and the whole section is counterstained as blue with hematoxylin. Folders are named after genotypes (WT or P-AdnTg+) and subfolders after time points (week 0, 2, 5, or 10) post initial dimerizer administration. Related to Figure 7A. DOI: http://dx.doi.org/10.7554/eLife.03851.031 [file elife03851s002.zip › Figure 7-source data 1/WT/Week 10/PA41-1s a.jpg]

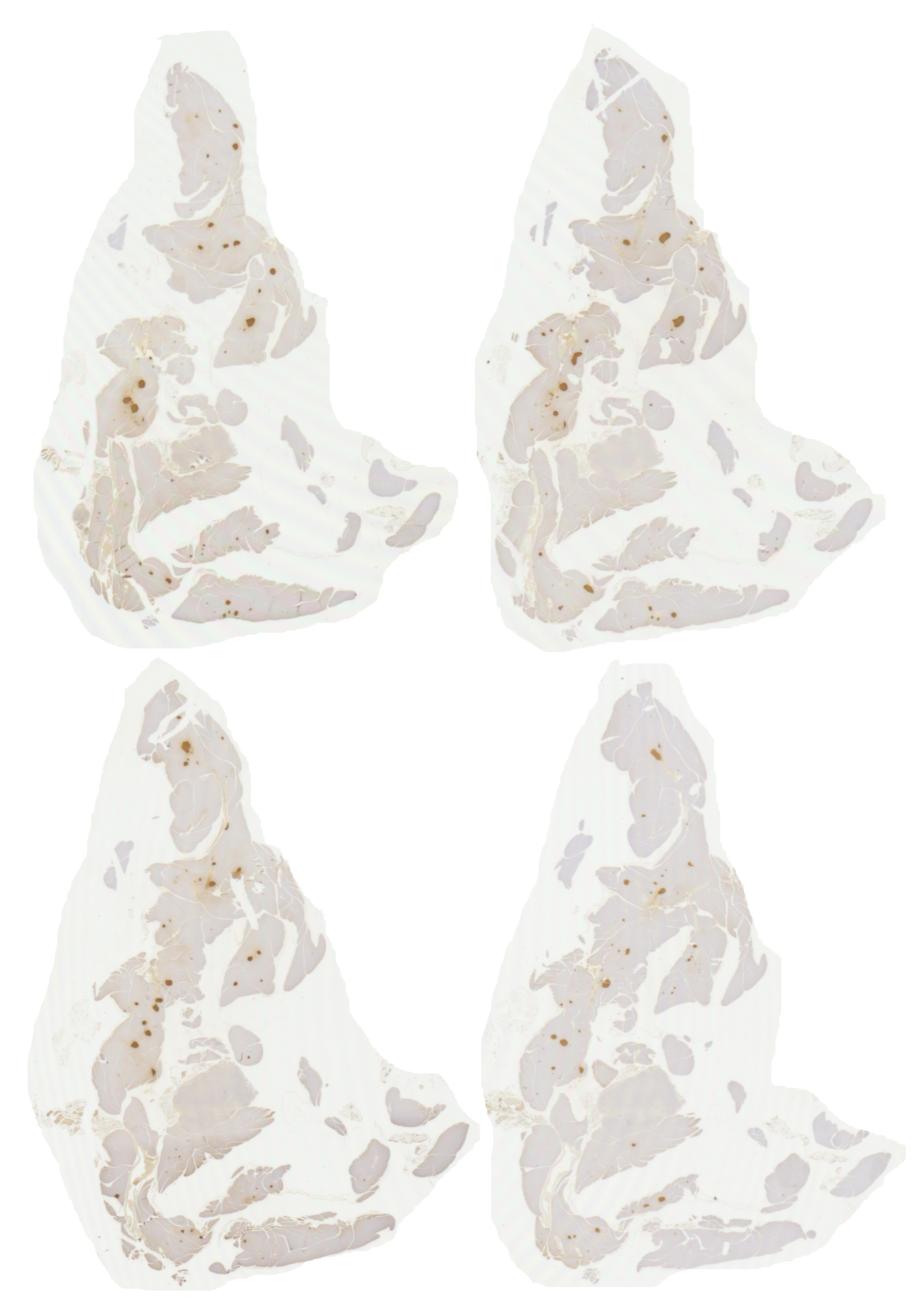

Supplement: Figure 7—source data 1. — The zip file contains all the 2400-dpi scanned images of insulin-immunostained pancreas sections used for quantitation of insulin-positive cell area. Insulin is stained as brown, and the whole section is counterstained as blue with hematoxylin. Folders are named after genotypes (WT or P-AdnTg+) and subfolders after time points (week 0, 2, 5, or 10) post initial dimerizer administration. Related to Figure 7A. DOI: http://dx.doi.org/10.7554/eLife.03851.031 [file elife03851s002.zip › Figure 7-source data 1/WT/Week 10/PA41-1s b.jpg]

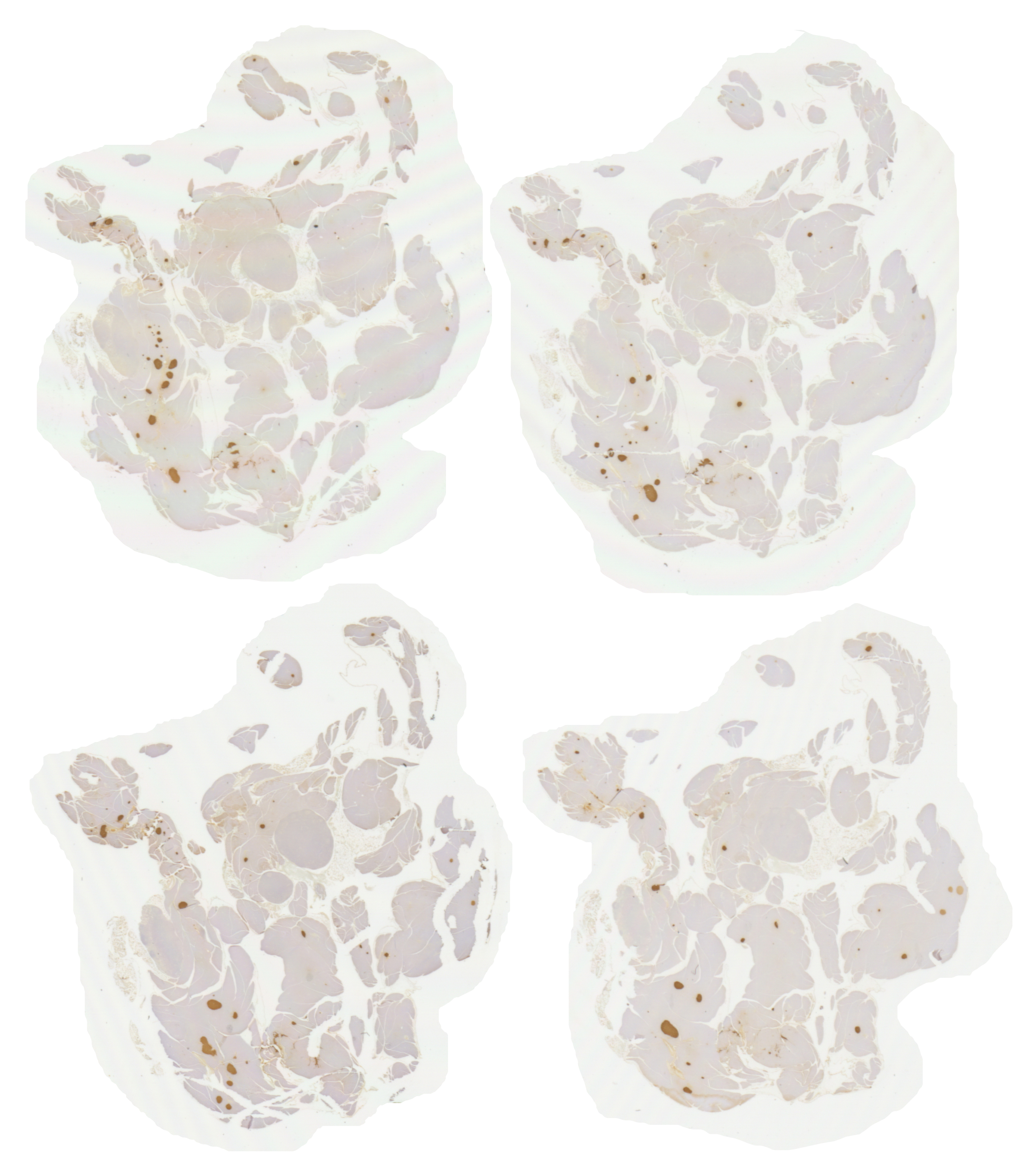

Supplement: Figure 7—source data 1. — The zip file contains all the 2400-dpi scanned images of insulin-immunostained pancreas sections used for quantitation of insulin-positive cell area. Insulin is stained as brown, and the whole section is counterstained as blue with hematoxylin. Folders are named after genotypes (WT or P-AdnTg+) and subfolders after time points (week 0, 2, 5, or 10) post initial dimerizer administration. Related to Figure 7A. DOI: http://dx.doi.org/10.7554/eLife.03851.031 [file elife03851s002.zip › Figure 7-source data 1/WT/Week 10/PA41-1s c.jpg]

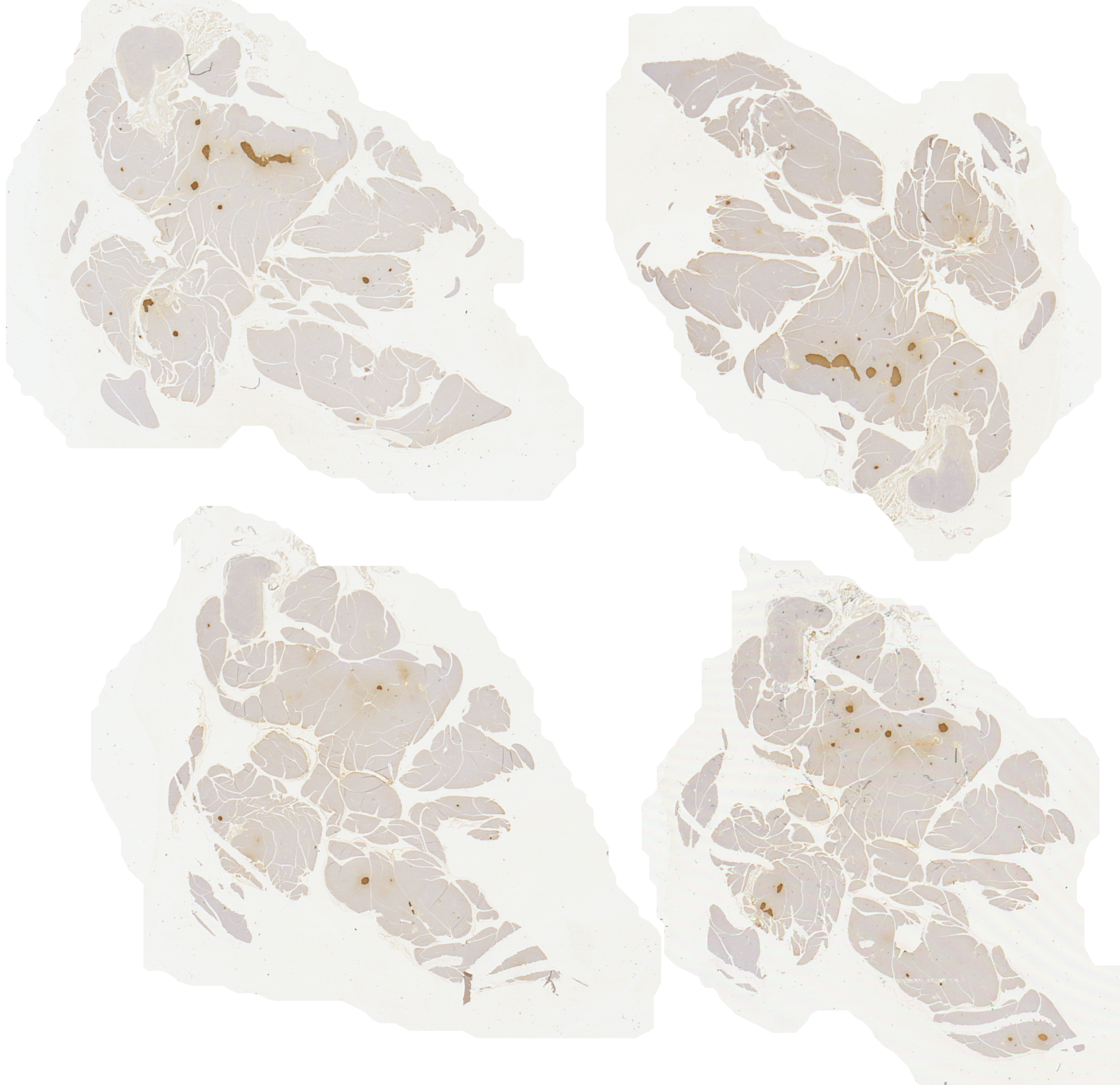

Supplement: Figure 7—source data 1. — The zip file contains all the 2400-dpi scanned images of insulin-immunostained pancreas sections used for quantitation of insulin-positive cell area. Insulin is stained as brown, and the whole section is counterstained as blue with hematoxylin. Folders are named after genotypes (WT or P-AdnTg+) and subfolders after time points (week 0, 2, 5, or 10) post initial dimerizer administration. Related to Figure 7A. DOI: http://dx.doi.org/10.7554/eLife.03851.031 [file elife03851s002.zip › Figure 7-source data 1/WT/Week 2/RLuc19 a.jpg]

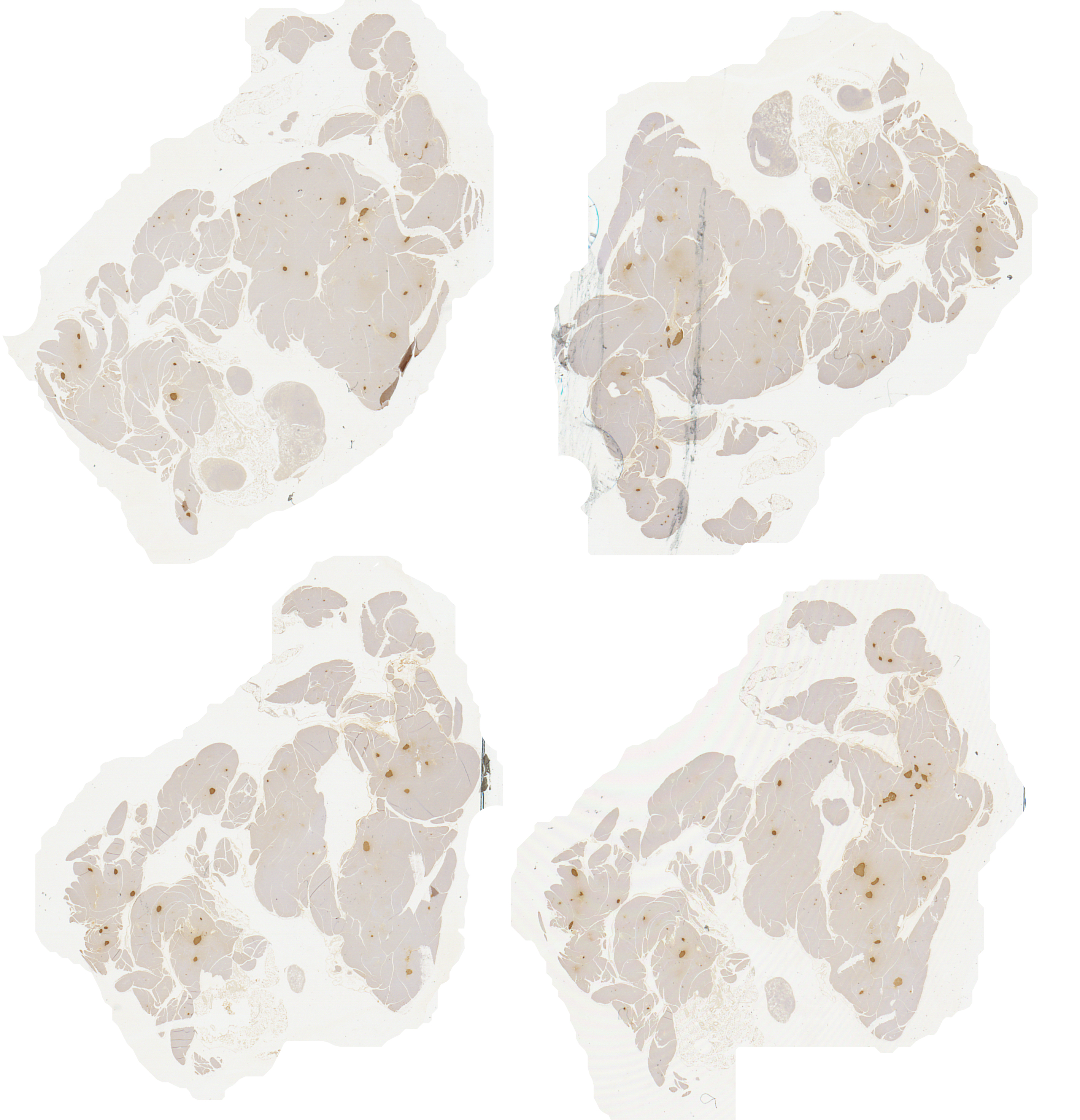

Supplement: Figure 7—source data 1. — The zip file contains all the 2400-dpi scanned images of insulin-immunostained pancreas sections used for quantitation of insulin-positive cell area. Insulin is stained as brown, and the whole section is counterstained as blue with hematoxylin. Folders are named after genotypes (WT or P-AdnTg+) and subfolders after time points (week 0, 2, 5, or 10) post initial dimerizer administration. Related to Figure 7A. DOI: http://dx.doi.org/10.7554/eLife.03851.031 [file elife03851s002.zip › Figure 7-source data 1/WT/Week 2/RLuc19 b.jpg]

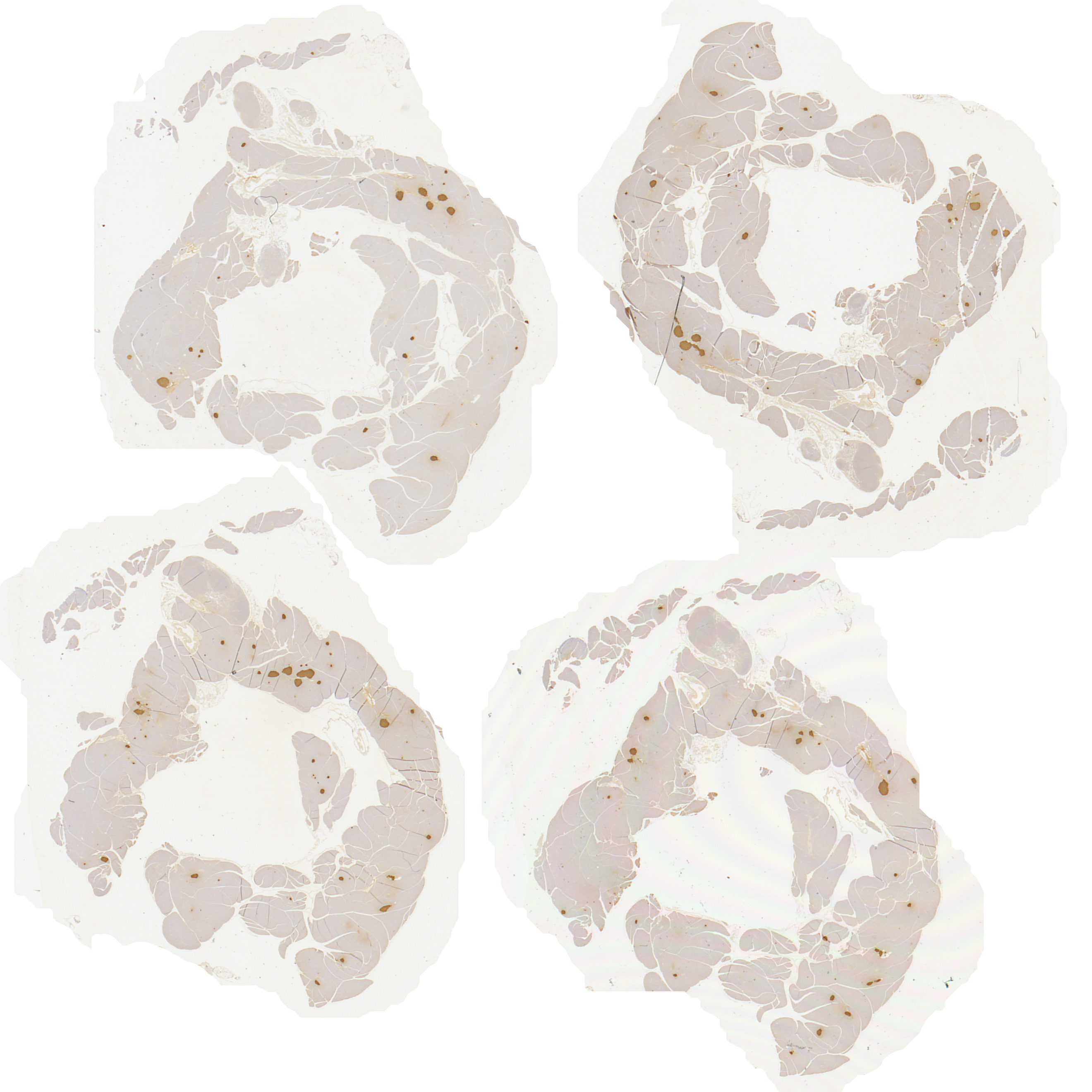

Supplement: Figure 7—source data 1. — The zip file contains all the 2400-dpi scanned images of insulin-immunostained pancreas sections used for quantitation of insulin-positive cell area. Insulin is stained as brown, and the whole section is counterstained as blue with hematoxylin. Folders are named after genotypes (WT or P-AdnTg+) and subfolders after time points (week 0, 2, 5, or 10) post initial dimerizer administration. Related to Figure 7A. DOI: http://dx.doi.org/10.7554/eLife.03851.031 [file elife03851s002.zip › Figure 7-source data 1/WT/Week 2/RLuc19 c.jpg]

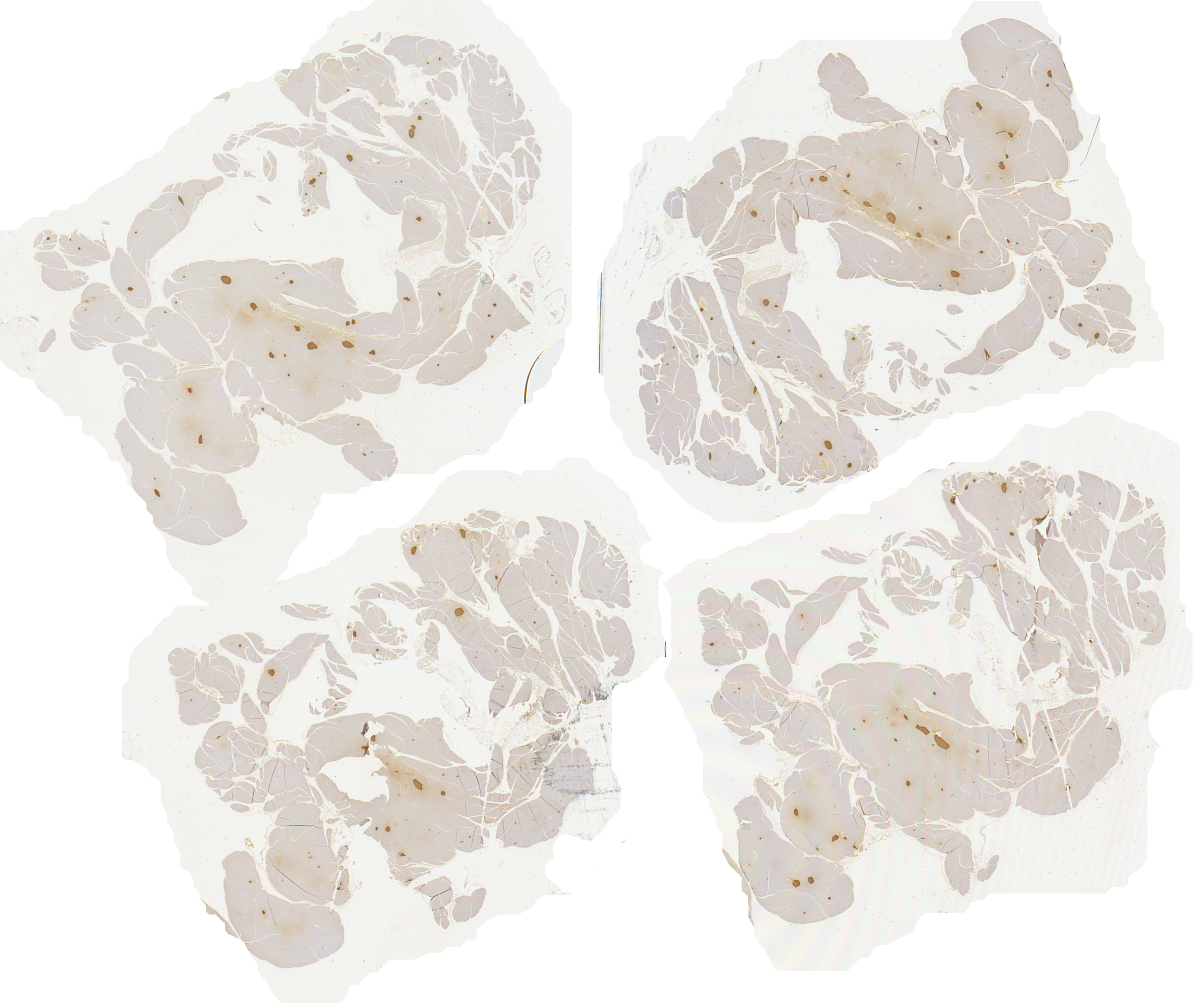

Supplement: Figure 7—source data 1. — The zip file contains all the 2400-dpi scanned images of insulin-immunostained pancreas sections used for quantitation of insulin-positive cell area. Insulin is stained as brown, and the whole section is counterstained as blue with hematoxylin. Folders are named after genotypes (WT or P-AdnTg+) and subfolders after time points (week 0, 2, 5, or 10) post initial dimerizer administration. Related to Figure 7A. DOI: http://dx.doi.org/10.7554/eLife.03851.031 [file elife03851s002.zip › Figure 7-source data 1/WT/Week 2/RLuc19 d.jpg]

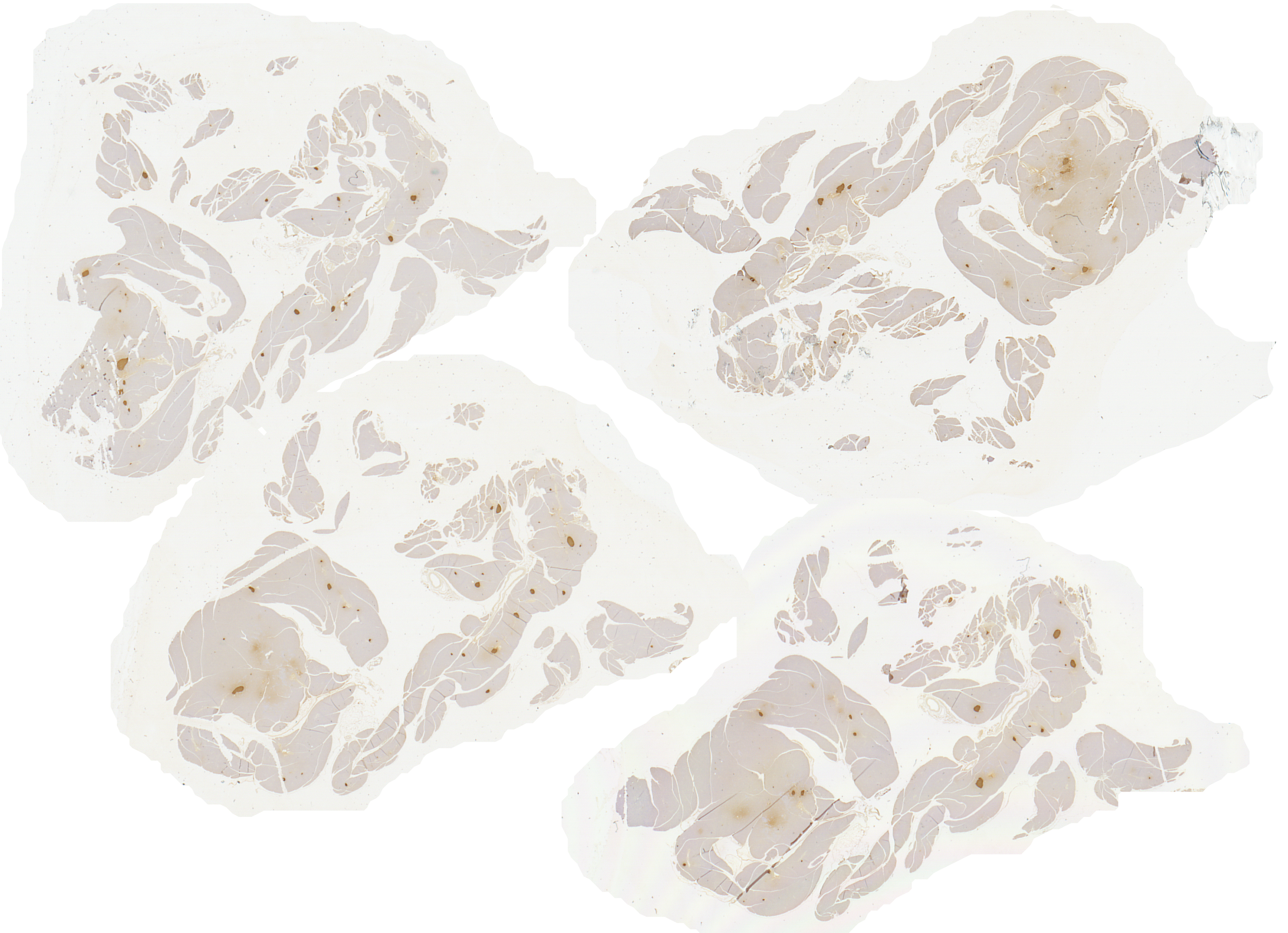

Supplement: Figure 7—source data 1. — The zip file contains all the 2400-dpi scanned images of insulin-immunostained pancreas sections used for quantitation of insulin-positive cell area. Insulin is stained as brown, and the whole section is counterstained as blue with hematoxylin. Folders are named after genotypes (WT or P-AdnTg+) and subfolders after time points (week 0, 2, 5, or 10) post initial dimerizer administration. Related to Figure 7A. DOI: http://dx.doi.org/10.7554/eLife.03851.031 [file elife03851s002.zip › Figure 7-source data 1/WT/Week 2/RLuc19 e.jpg]

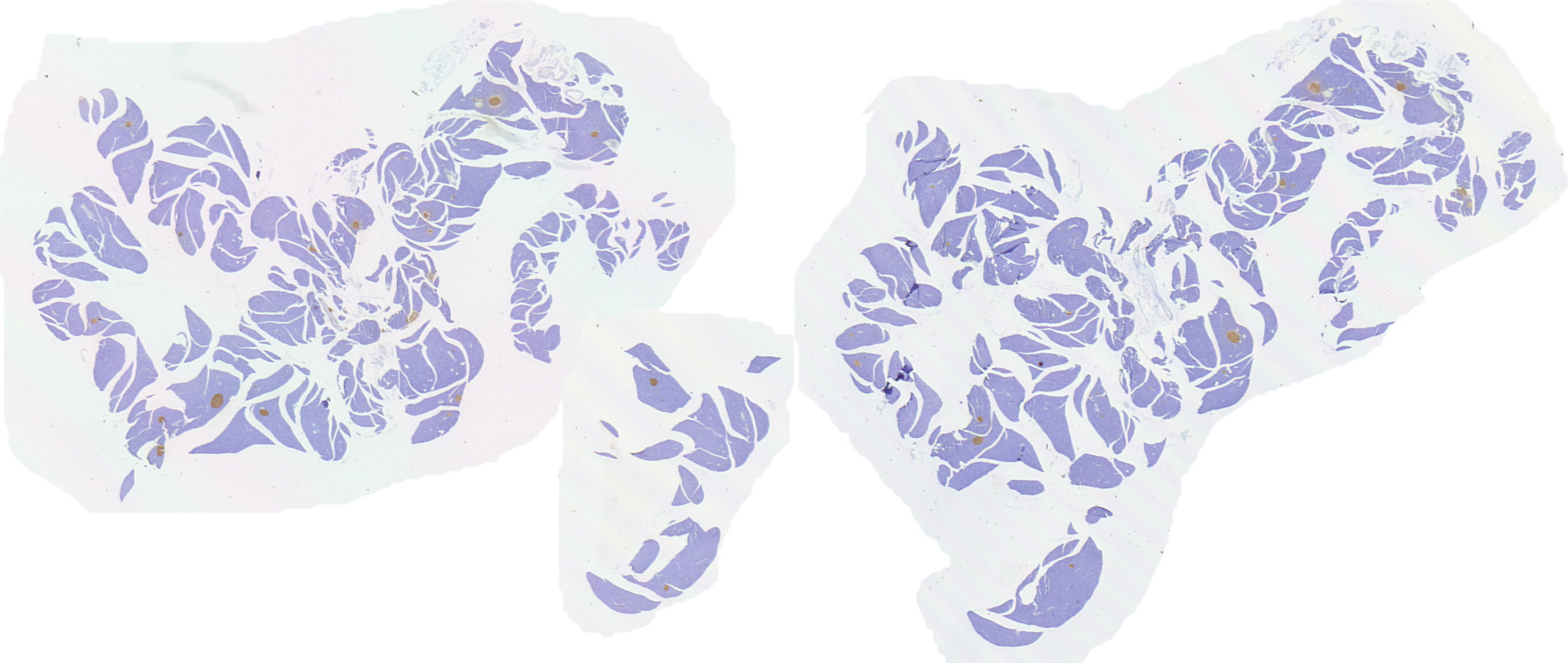

Supplement: Figure 7—source data 1. — The zip file contains all the 2400-dpi scanned images of insulin-immunostained pancreas sections used for quantitation of insulin-positive cell area. Insulin is stained as brown, and the whole section is counterstained as blue with hematoxylin. Folders are named after genotypes (WT or P-AdnTg+) and subfolders after time points (week 0, 2, 5, or 10) post initial dimerizer administration. Related to Figure 7A. DOI: http://dx.doi.org/10.7554/eLife.03851.031 [file elife03851s002.zip › Figure 7-source data 1/WT/Week 5/PA44-1s a.jpg]

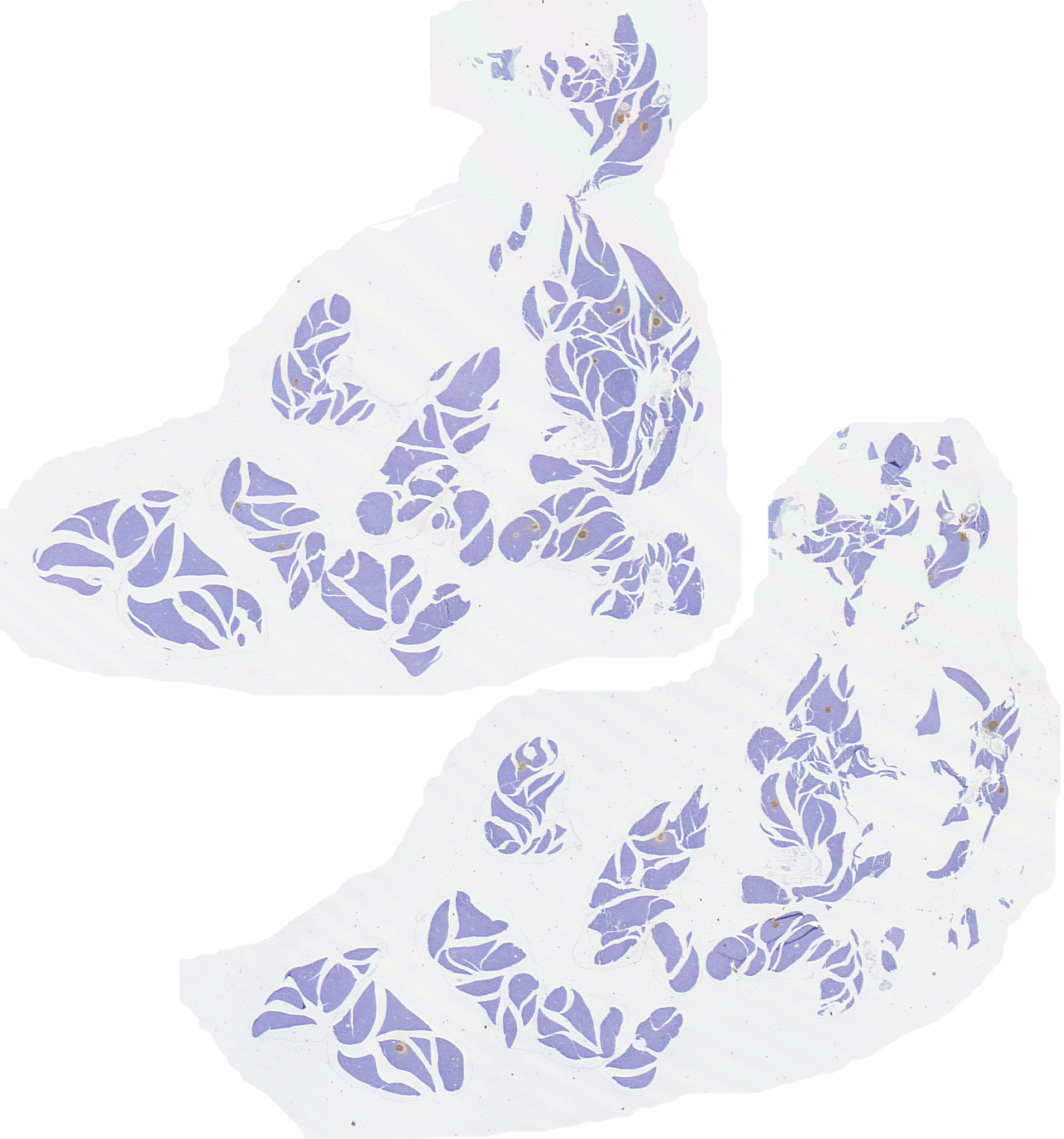

Supplement: Figure 7—source data 1. — The zip file contains all the 2400-dpi scanned images of insulin-immunostained pancreas sections used for quantitation of insulin-positive cell area. Insulin is stained as brown, and the whole section is counterstained as blue with hematoxylin. Folders are named after genotypes (WT or P-AdnTg+) and subfolders after time points (week 0, 2, 5, or 10) post initial dimerizer administration. Related to Figure 7A. DOI: http://dx.doi.org/10.7554/eLife.03851.031 [file elife03851s002.zip › Figure 7-source data 1/WT/Week 5/PA44-1s b.jpg]

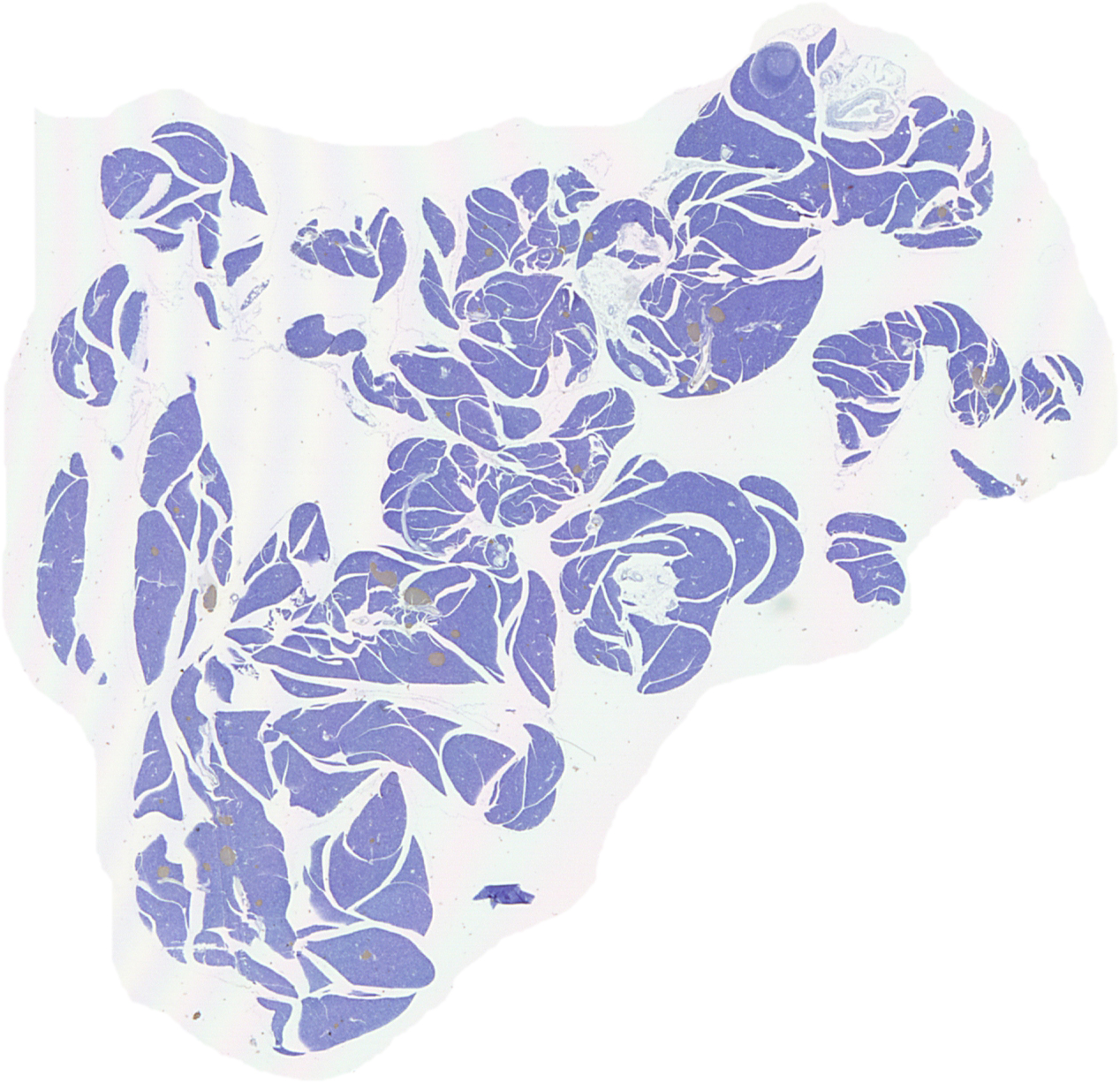

Supplement: Figure 7—source data 1. — The zip file contains all the 2400-dpi scanned images of insulin-immunostained pancreas sections used for quantitation of insulin-positive cell area. Insulin is stained as brown, and the whole section is counterstained as blue with hematoxylin. Folders are named after genotypes (WT or P-AdnTg+) and subfolders after time points (week 0, 2, 5, or 10) post initial dimerizer administration. Related to Figure 7A. DOI: http://dx.doi.org/10.7554/eLife.03851.031 [file elife03851s002.zip › Figure 7-source data 1/WT/Week 5/PA44-1s-B1 a.jpg]

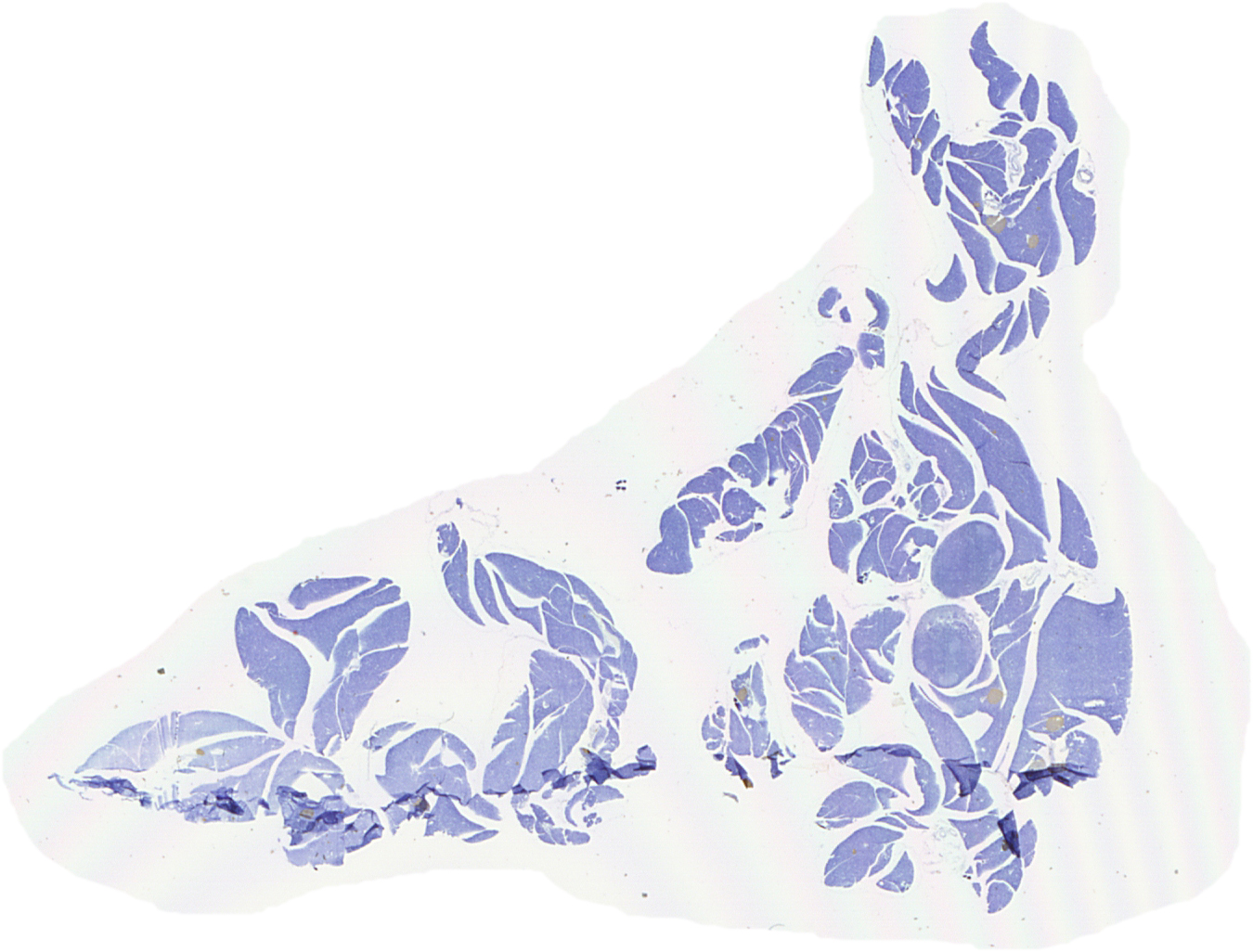

Supplement: Figure 7—source data 1. — The zip file contains all the 2400-dpi scanned images of insulin-immunostained pancreas sections used for quantitation of insulin-positive cell area. Insulin is stained as brown, and the whole section is counterstained as blue with hematoxylin. Folders are named after genotypes (WT or P-AdnTg+) and subfolders after time points (week 0, 2, 5, or 10) post initial dimerizer administration. Related to Figure 7A. DOI: http://dx.doi.org/10.7554/eLife.03851.031 [file elife03851s002.zip › Figure 7-source data 1/WT/Week 5/PA44-1s-B1 b.jpg]

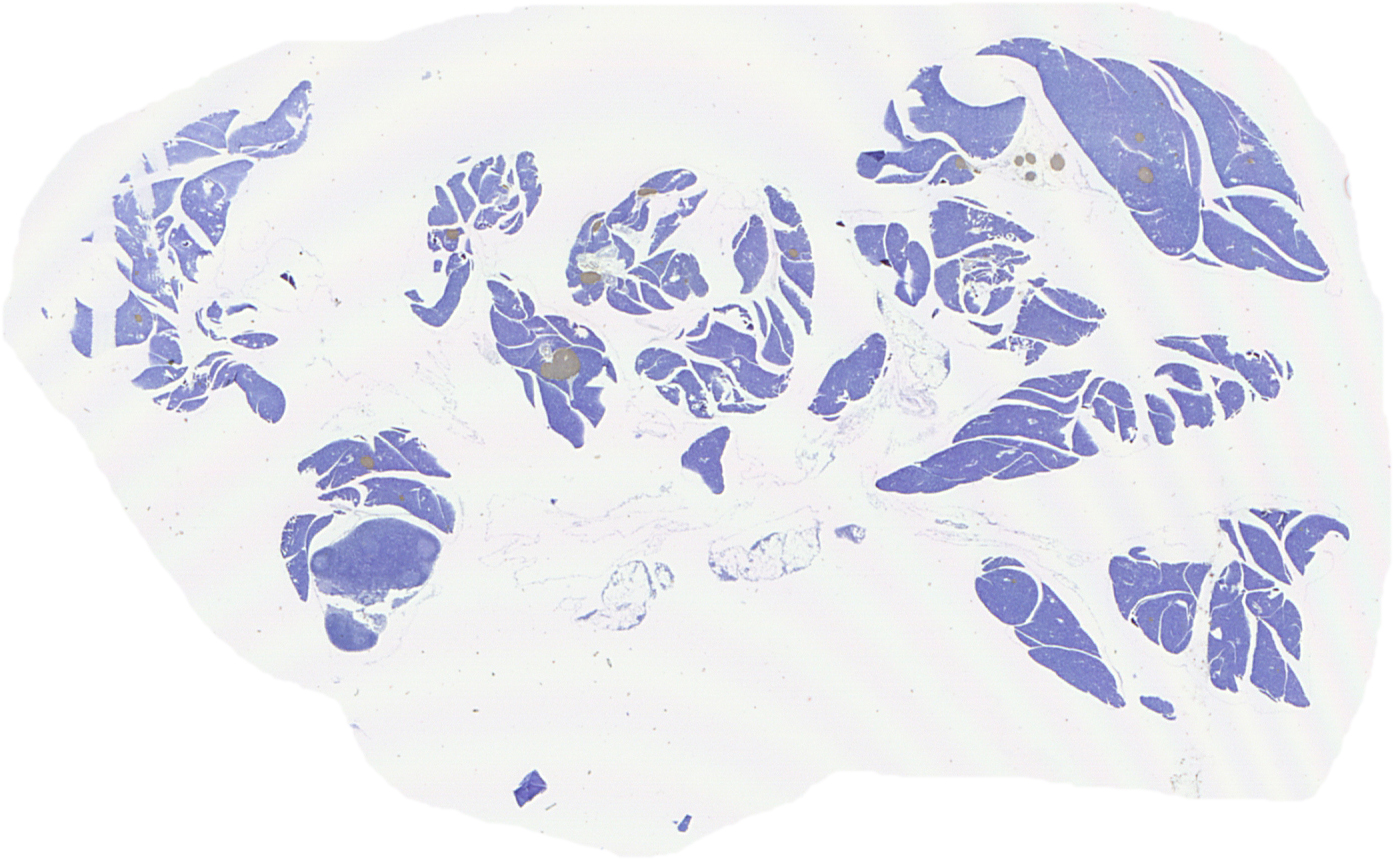

Supplement: Figure 7—source data 1. — The zip file contains all the 2400-dpi scanned images of insulin-immunostained pancreas sections used for quantitation of insulin-positive cell area. Insulin is stained as brown, and the whole section is counterstained as blue with hematoxylin. Folders are named after genotypes (WT or P-AdnTg+) and subfolders after time points (week 0, 2, 5, or 10) post initial dimerizer administration. Related to Figure 7A. DOI: http://dx.doi.org/10.7554/eLife.03851.031 [file elife03851s002.zip › Figure 7-source data 1/WT/Week 5/PA44-1s-B1 c.jpg]

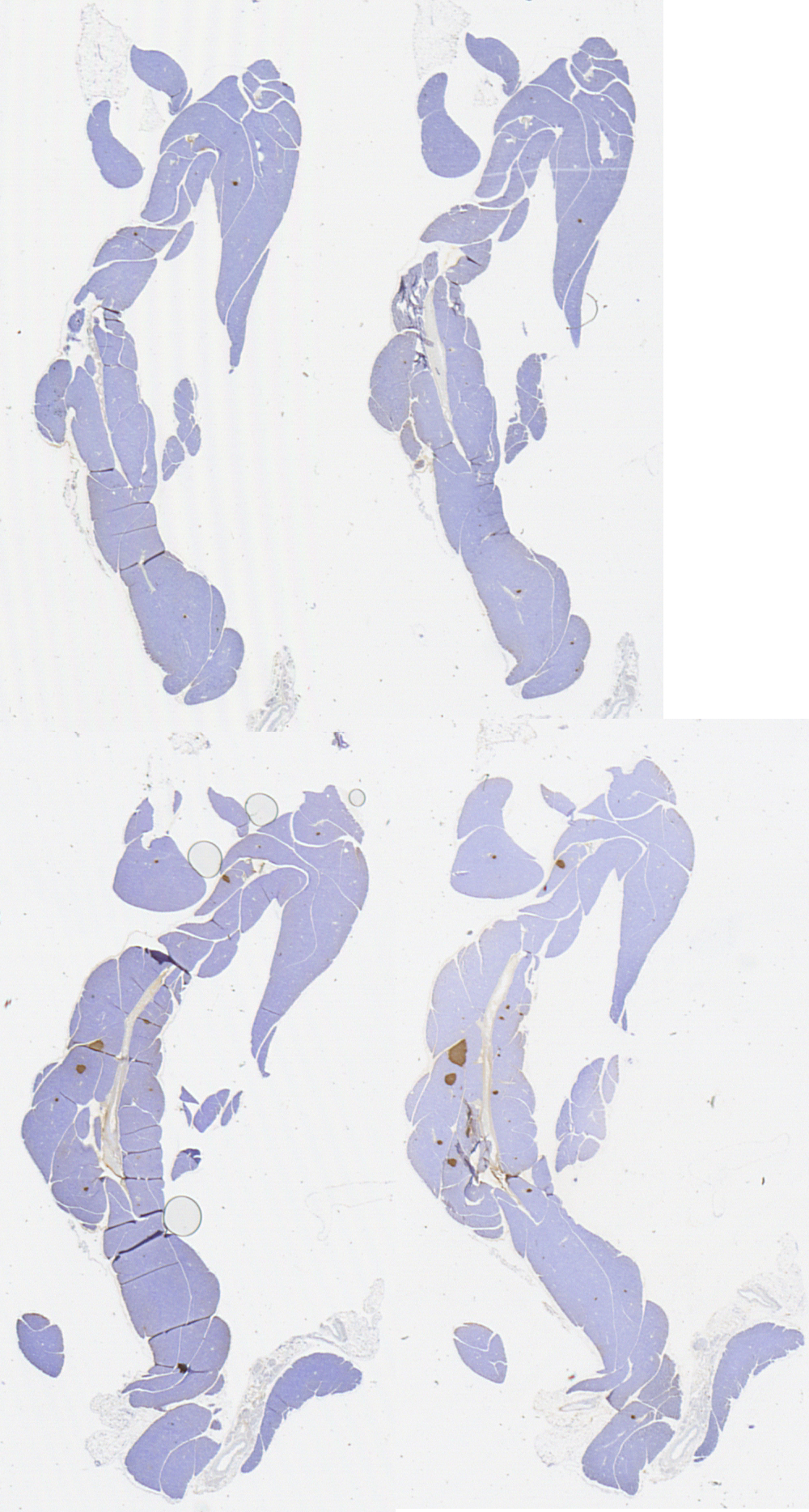

Supplement: Figure 7—source data 1. — The zip file contains all the 2400-dpi scanned images of insulin-immunostained pancreas sections used for quantitation of insulin-positive cell area. Insulin is stained as brown, and the whole section is counterstained as blue with hematoxylin. Folders are named after genotypes (WT or P-AdnTg+) and subfolders after time points (week 0, 2, 5, or 10) post initial dimerizer administration. Related to Figure 7A. DOI: http://dx.doi.org/10.7554/eLife.03851.031 [file elife03851s002.zip › Figure 7-source data 1/WT/Week 5/PA69 a.jpg]

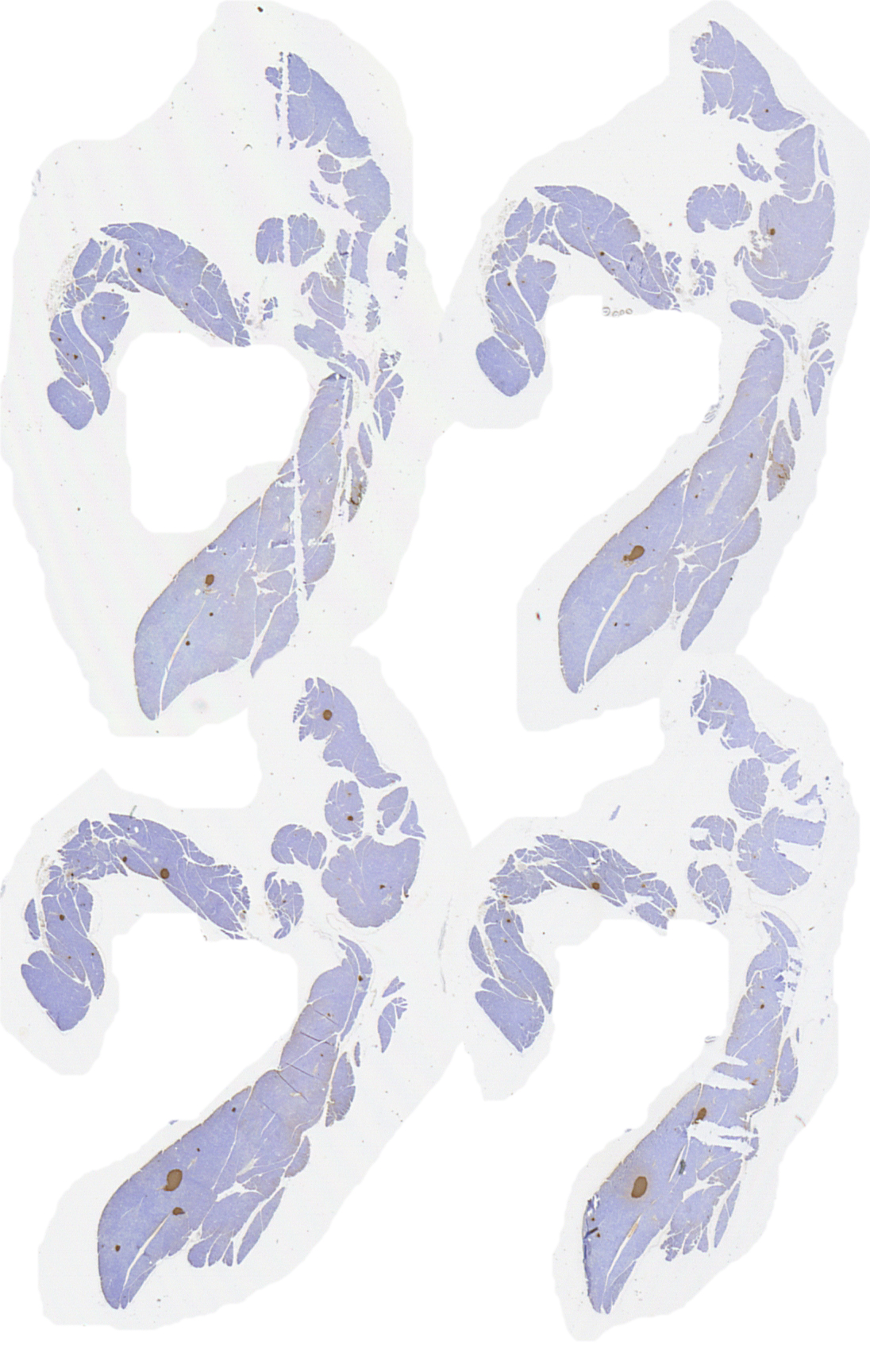

Supplement: Figure 7—source data 1. — The zip file contains all the 2400-dpi scanned images of insulin-immunostained pancreas sections used for quantitation of insulin-positive cell area. Insulin is stained as brown, and the whole section is counterstained as blue with hematoxylin. Folders are named after genotypes (WT or P-AdnTg+) and subfolders after time points (week 0, 2, 5, or 10) post initial dimerizer administration. Related to Figure 7A. DOI: http://dx.doi.org/10.7554/eLife.03851.031 [file elife03851s002.zip › Figure 7-source data 1/WT/Week 5/PA69 b.jpg]

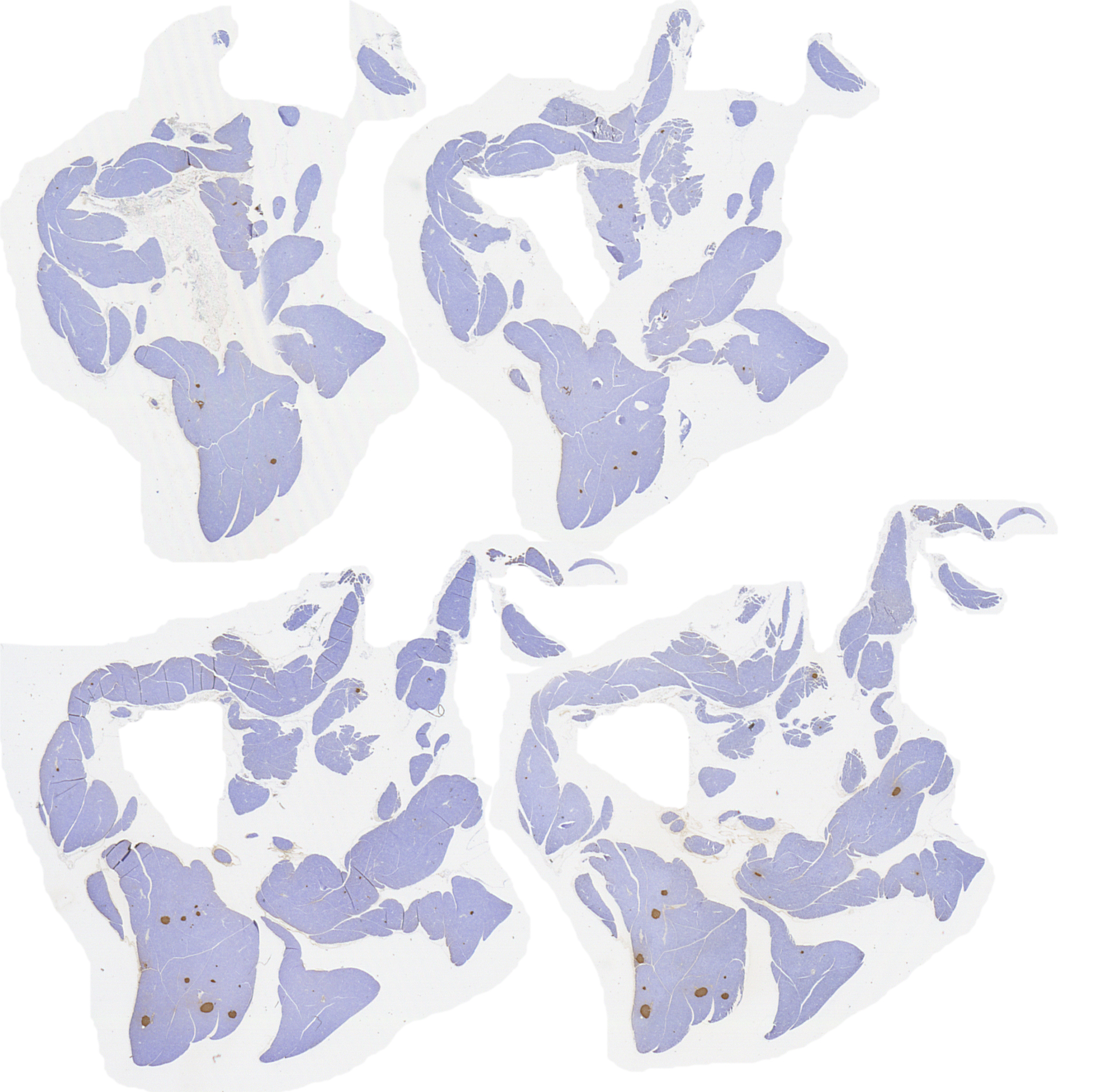

Supplement: Figure 7—source data 1. — The zip file contains all the 2400-dpi scanned images of insulin-immunostained pancreas sections used for quantitation of insulin-positive cell area. Insulin is stained as brown, and the whole section is counterstained as blue with hematoxylin. Folders are named after genotypes (WT or P-AdnTg+) and subfolders after time points (week 0, 2, 5, or 10) post initial dimerizer administration. Related to Figure 7A. DOI: http://dx.doi.org/10.7554/eLife.03851.031 [file elife03851s002.zip › Figure 7-source data 1/WT/Week 5/PA69 c.jpg]

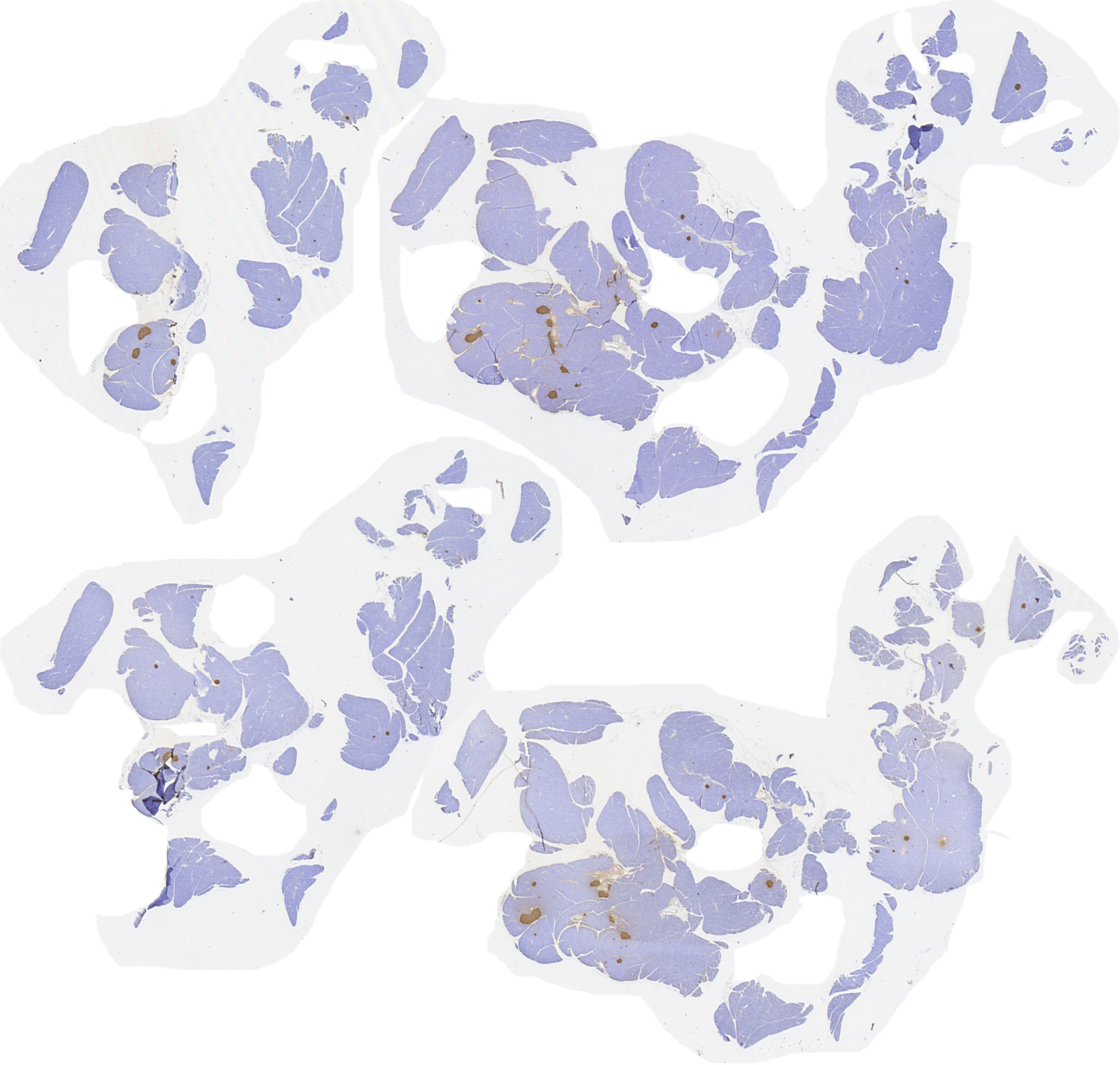

Supplement: Figure 7—source data 1. — The zip file contains all the 2400-dpi scanned images of insulin-immunostained pancreas sections used for quantitation of insulin-positive cell area. Insulin is stained as brown, and the whole section is counterstained as blue with hematoxylin. Folders are named after genotypes (WT or P-AdnTg+) and subfolders after time points (week 0, 2, 5, or 10) post initial dimerizer administration. Related to Figure 7A. DOI: http://dx.doi.org/10.7554/eLife.03851.031 [file elife03851s002.zip › Figure 7-source data 1/WT/Week 5/PA69 d.jpg]
