# Supplementary material for: Adiponectin is essential for lipid homeostasis and survival under insulin deficiency and promotes β-cell regeneration
Source: eLife. 2014 Oct 23;3:e03851. doi: 10.7554/eLife.03851 (PMC4228265; doi:10.7554/eLife.03851)
Supplement: Supplementary file 1. — Primers for RT-qPCR. DOI: http://dx.doi.org/10.7554/eLife.03851.037 [file elife03851s003.pdf]

Supplementary file 1. Primers for RT-qPCR

Gene	Forward Primer	Reverse Primer
18S rRNA	5'-GGAGAGGGAGCCTGAGAAACG-3'	5'-CGCTCCCAAGATCCAACTACG-3'
Abcg5	5'-CTGCTCGCCTACGTGCTACACGTCC-3'	5'-CTATACCAAGCAGCACAAGTGTTAGAAATTCTC-3'
Abcg8	5'-TGCTCATCTCCCTCCACCAGCCTC-3'	5'-GCCTCAGCTTTCCACAGAAAGTCATCAA-3'
Acaca	5'-GAGCTGACTGACGGCCAGATCCAG-3'	5'-ATCACAGAGCGGACGCCATCTTCCT-3'
Acacb	5'-CTGAAGGGGACCTGGCAGAGTGGAT-3'	5'-GCAGGGTCAGCGGGGACAGC-3'
Acox1	5'-GGGCACGGCTATTCTCACAGCAG-3'	5'-ATACGCTGGCTCGGCAGGTCATT-3'
Acss2	5'-GTCAAACATCTGGGGCGGGC-3'	5'-CTGGTGTACAAGATGAAGAGTGGGTCCTC-3'
ChREBP	5'-AACTGGAAGTTCTGGGTGTTCAGCA-3'	5'-GAAGTGCTGAGTTGGCGAAGGG-3'
Cpt1a	5'-CTTTGGGCCGGTTGCTGATG-3'	5'-CATGGCTTGTCTCAAGTGCTTCCC-3'
Dgat2	5'-TGCGCCATGGAGCTGATCTG-3'	5'-GCTCCAGCTTGGGGACAGTGATG-3'
Fabp1	5'-AGGCAATAGGTCTGCCCGAGG-3'	5'-GCCCAATGTCATGGTATTGGTGATT-3'
Fasn	5'-ATAAAGCAGTTTCTTGATGTGGAACACAGC-3'	5'-CCCGTCACACACCTGGGAGAG-3'
FoxO1	5'-GCGACAGCAACAGCTCGGC-3'	5'-TCTCCGGGGTGATTTTCCGC-3'
G6pc	5'-GAGACCGGACCAGGAAGTCCC-3'	5'-AGCCACAGCAATGCCTGACAAG-3'
Gpam	5'-CGAGATTGCTATCTCAAGGTGAGCCAG-3'	5'-TTGGTCTCTTTGAAAACCCCGATG-3'
Hnf4a	5'-GCATGGCCAAGATTGACAACCTG-3'	5'-CTCGGGAGTGGCTGCCTGC-3'
Igfbp1	5'-ACCTCAAGAAATGGAAGGAGCCCTG-3'	5'-GGGATTTTCTTTCCACTCCATGGGTAG-3'
Insig-1	5'-ATCAACCACGCCAGTGCCAATTAG-3'	5'-TGTGAGGCTTTTCCGGAACACC-3'
Insig-2	5'-GTGTTCGTGGGTATAAATCACGCCAG-3'	5'-CAGCAATAACTTTGCATTCATACATTGCC-3'
Lpl	5'-CCGGAGAGACTCAGAAAAAGGTCATC-3'	5'-ACCCACTTTCAAACACCCAAACAAG-3'
Nr1h2	5'-GCGTCCACCATTGAGATCATGTTG-3'	5'-GGGAAGCGGAGCTGGTCCTG-3'
Nr1h3	5'-ACCGGGAAGACTTTGCCAAAGC-3'	5'-ACATCAGTGGGTCGTGGGGG-3'
Pck1	5'-ACCGCAGGACGCGGAACC-3'	5'-CGTGCATGATGATCTTGCCCTTG-3'
Pcx	5'-GCAGAGGAGTTTGAGGTTGAGCTGG-3'	5'-ATGGAAGTGCATCTCCTTCATGGC-3'
Pklr	5'-TGTGCCACACAGATGCTGGAGAG-3'	5'-GGTCGGTAGCGAGACAGAAGCTGAG-3'
Ppara	5'-ACAAAGACGGGATGCTGATCGC-3'	5'-AAGGCCAGGCCGATCTCCAC-3'
Scd1	5'-GCTGGTGATGTTCCAGAGGAGGTACTAC-3'	5'-TAGTTGTGGAAGCCCTCGCCC-3'
Srebf1	5'-CGTGCAGGCAGGTCCCC-3'	5'-CGCAAGACAGCAGATTTATTCAGCTTT-3'
Srebp-1a	5'-GGGGAACTTTTCCTTAACGTGGGC-3'	5'-GATGAGCTGGAGCATGTCTTCGATG-3'
Gapdh	5′-AACTTTGGCATTGTGGAAGG-3′	5′-ACACATTGGGGGTAGGAACA-3′
